# Macrocyclic Donor‐Acceptor Dyads Composed of Oligothiophene Half‐Cycles and Perylene Bisimides

**DOI:** 10.1002/chem.202200355

**Published:** 2022-04-12

**Authors:** Kevin Bold, Matthias Stolte, Kazutaka Shoyama, Ana‐Maria Krause, Alexander Schmiedel, Marco Holzapfel, Christoph Lambert, Frank Würthner

**Affiliations:** ^1^ Institut für Organische Chemie Universität Würzburg Am Hubland 97074 Würzburg Germany; ^2^ Center for Nanosystems Chemistry (CNC) Universität Würzburg Theodor-Boveri-Weg 97074 Würzburg Germany

**Keywords:** donor-acceptor dyad, macrocycle, oligothiophene, perylene bisimide, photoinduced electron transfer

## Abstract

A series of donor‐acceptor (D−A) macrocyclic dyads consisting of an electron‐poor perylene bisimide (PBI) π‐scaffold bridged with electron‐rich α‐oligothiophenes bearing four, five, six and seven thiophene units between the two phenyl‐imide substituents has been synthesized and characterized by steady‐state UV/Vis absorption and fluorescence spectroscopy, cyclic and differential pulse voltammetry as well as transient absorption spectroscopy. Tying the oligothiophene strands in a conformationally fixed macrocyclic arrangement leads to a more rigid π‐scaffold with vibronic fine structure in the respective absorption spectra. Electrochemical analysis disclosed charged state properties in solution which are strongly dependent on the degree of rigidification within the individual macrocycle. Investigation of the excited state dynamics revealed an oligothiophene bridge size‐dependent fast charge transfer process for the macrocyclic dyads upon PBI subunit excitation.

## Graphical abstract

**The synthesis and properties** of perylene bisimide dyes bridged by α‐oligothiophenes bearing four, five, six and seven thiophene units is described. Next to panchromatic light absorption in the UV/Vis spectrum, the compounds show donor bridge size‐dependent electronic properties and excited state dynamics.

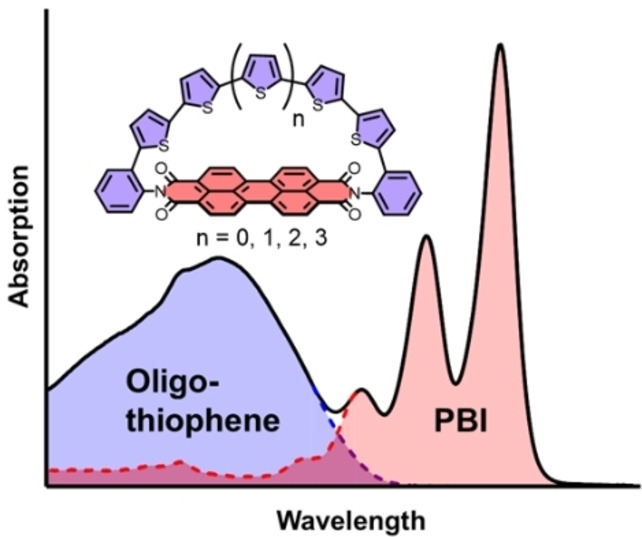

## Introduction

Ensuing from the fascination of chemists towards π‐conjugated macrocycles, great synthetic and scientific progress has been made in the last two decades. After early attempts of Vögtle and co‐workers in the 1990 s^[1]^ to synthesize [*n*]cycloparaphenylenes ([*n*]CPPs), it was the groups of Jasti and Bertozzi who reported the synthesis of the first nanohoops in 2008 utilizing a remarkably similar strategy.^[2]^ This was followed by two more decisive synthetic cornerstones in this research field by the groups of Itami^[3]^ and Yamago.^[4]^ Interestingly, much earlier back in 2000 Bäuerle and co‐workers already reported the first synthesis of another class of conjugated macrocycles, namely cyclo[*n*]oligothiophenes (C[*n*]Ts).[^5^, ^6a^, ^6b^, ^6c^] Further development of these early synthetic successes lead to giant C[*n*]Ts comprising up to 35 thiophene rings in excellent yields.^[7]^ Since then, a variety of versatile and efficient synthetic protocols were developed to bend initially planar π‐conjugated chains into macrocyclic arrangements.^[8]^

**Figure 1:**
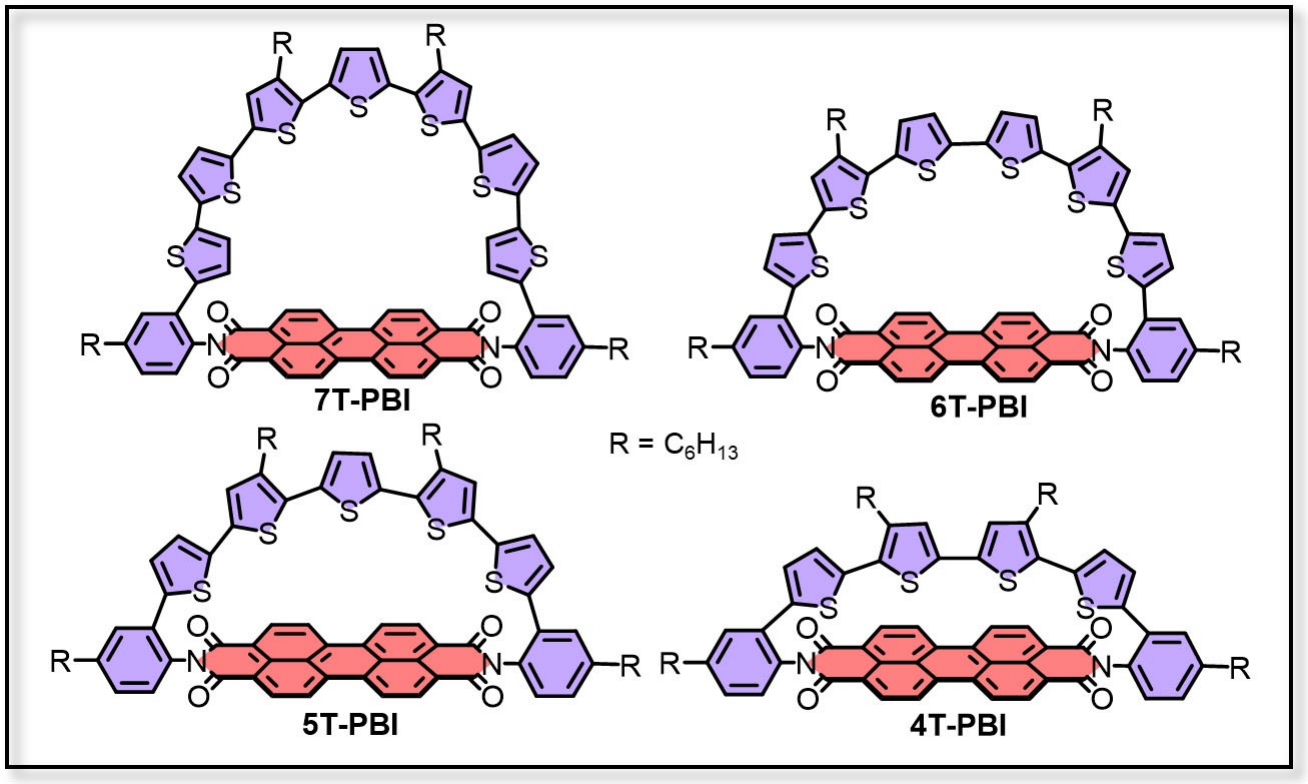
Molecular structures discussed in this work.

The implementation of donor‐acceptor (D−A) motifs into π‐conjugated macrocycles received great attention in recent years.[^9a^, ^9b^, ^9c^, ^9d^, ^9e^, ^9f^, ^9g^] Numerous structures were developed to alter the electrooptical properties by adjusting the respective HOMO and LUMO levels of D and A. This electronic fine‐tuning is achieved by careful selection of subunit structures comprising different electron accepting and donating capabilities, respectively. The bithiophene bay‐bridged perylene bisimide (PBI) macrocycles of Nuckolls and co‐workers^[10]^ display one great example for such a tailored D−A system. Also non‐cyclic covalent donor‐acceptor linkages[^11a^, ^11b^, ^11c^] of, for example, PBI‐oligothiophene dyads were described within the past two decades.^[12]^ Installation of thiophene moieties in the PBI's bay position leads to a bathochromic shift of the absorption compared to the unsubstituted PBI as well as fluorescence quenching due to charge transfer (CT) processes.^[13]^ However, the introduction of oligothiophenes at the imide position more or less ensures electronic decoupling of both segments from each other due to nodal planes in the HOMO and LUMO of the PBI at the nitrogen atom.[^14a^, ^14b^] Therefore, the absorption properties of such dyads are often constituted of a sum of the respective subunit spectra. Furthermore, these dyads often show panchromic light absorption and an efficient photoinduced electron transfer (PET) leading to the fast formation of a charge separated state which often quenches the fluorescence of initially highly emissive individual chromophores. This fast tailored charge separation by concomitantly decelerated or impeded charge recombination could offer interesting prospects towards the understanding and general design of organic photovoltaic devices.^[15]^ By exploiting the advances of macrocyclic π‐systems and imide linked PBI‐oligothiophene dyads research we envisioned to combine both fields (Figure 1) and enlarge the toolbox of a substance class introduced by our group in a recent report.^[16]^

**Scheme 1:**
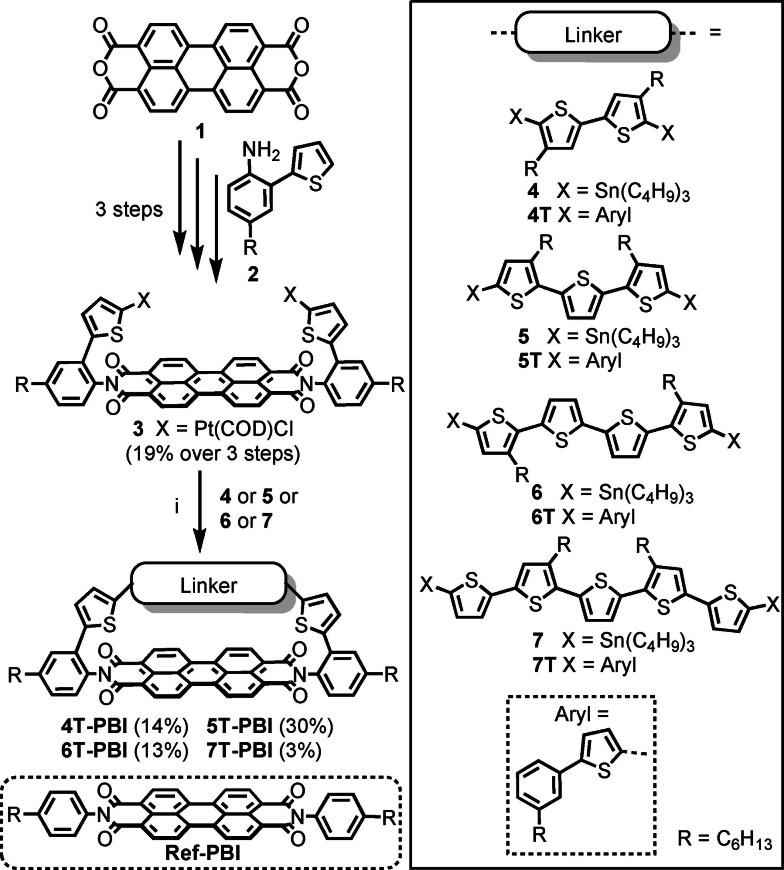
Synthesis of the macrocyclic architectures **4T‐PBI**, **5T‐PBI**,^[16]^ **6T‐PBI** and **7T‐PBI**. Reagents and conditions: i) toluene, 75 °C, overnight, then dppf, CH_2_Cl_2_, r.t, 6 h, then *m*‐xylene, 120 °C, overnight. COD=1,5‐cyclooctadiene. dppf=1,1’‐bis(diphenylphosphino)ferrocene.

Besides this previously reported macrocycle **5T**‐**PBI** (Scheme 1) we synthesized three novel differently sized oligothiophene semicircles whose ends are connected by an accepting PBI moiety. As demonstrated for a series of PBIs with a different number of thiophene donor units in bay position,^[17]^ changing the semicircle size in the strained macrocyclic scaffolds could potentially also fine tune the exited state dynamics. By changing the oligothiophene chain length in those dyads we also intended to influence the macrocyclic geometry and therefore especially highlight strain induced structural changes.

## Results and Discussion

### Synthesis and structural characterization

The synthesis started with the platinated thiophene‐PBI precursor **3** comprising the accepting PBI moiety (red) and two of the donating thiophene units (blue) which was obtained after three consecutive synthetic steps which were recently reported by our group.^[16]^ By combining this precursor with the stannylated α‐oligothiophene bridge structures bearing two (**4**) to five (**7**) thiophene subunits in a three step Pt‐mediated cross coupling reaction, macrocycles **4T**‐**PBI**, **5T**‐**PBI**,^[16]^ **6T**‐**PBI** and **7T**‐**PBI** could be synthesized in moderate yields of 14 %, 30 %, 13 % and 3 % (Scheme 1) after thorough purification via flash column‐ and gel permeation chromatography.

**Figure 2:**
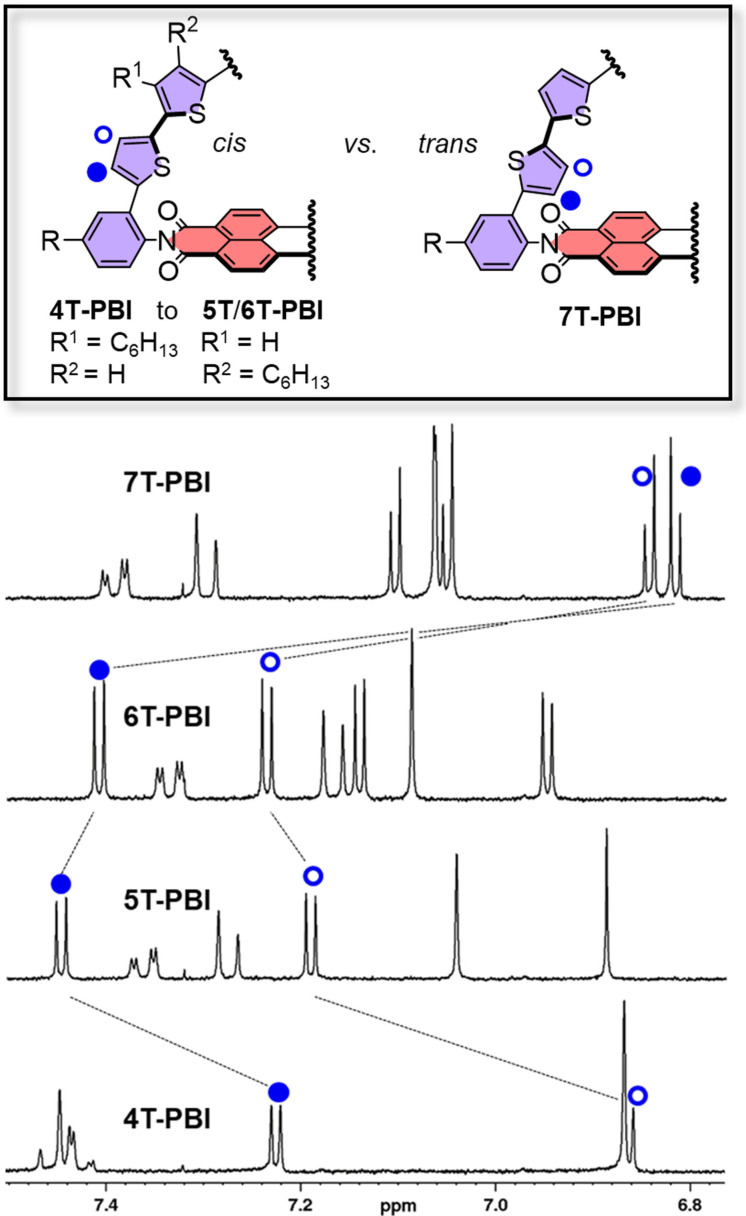
Excerpt of the ^1^H NMR spectra of **4T**‐**PBI**, **5T**‐**PBI**,^[16]^ **6T**‐**PBI** and **7T**‐**PBI** (from bottom to top) in CD_2_Cl_2_ at room temperature.

To overcome competing polymerization, the stannylated oligothiophenes **4**–**7** were added dropwise over 15 h to a solution of **3** in toluene at 75 °C. In this way, *pseudo* high‐dilution conditions favor the intramolecular complex formation over intermolecular side reactions. We utilized toluene in step i (Scheme 1) to apply elevated temperatures and exploit the templating effect of this solvent, as previously shown by our group for the synthesis of PBI cyclophanes.^[18]^ More detailed information about the synthetic procedures as well as a possible reaction mechanism for the macrocyclization cascade reactions can be found in the Supporting Information. The stannylated α‐oligothiophene backbones **4**–**7** and the reference compounds **4T**‐**7T** ^[19]^ could readily be synthesized following literature known Pd‐catalyzed cross coupling protocols.^[20]^ Aliphatic hexyl chains at the phenyl imide substituents and thiophene bridge of the final macrocyclic architectures **4T**‐**PBI** to **7T**‐**PBI** ensure sufficient solubility even in nonpolar cyclohexane. All new target molecules **4T**‐**PBI**, **6T**‐**PBI** and **7T**‐**PBI** were structurally characterized by nuclear magnetic resonance (NMR, (Figure 2)) as well as optical spectroscopy (UV/Vis absorption, fluorescence) and high‐resolution mass spectrometry (HRMS).

**Figure 3:**
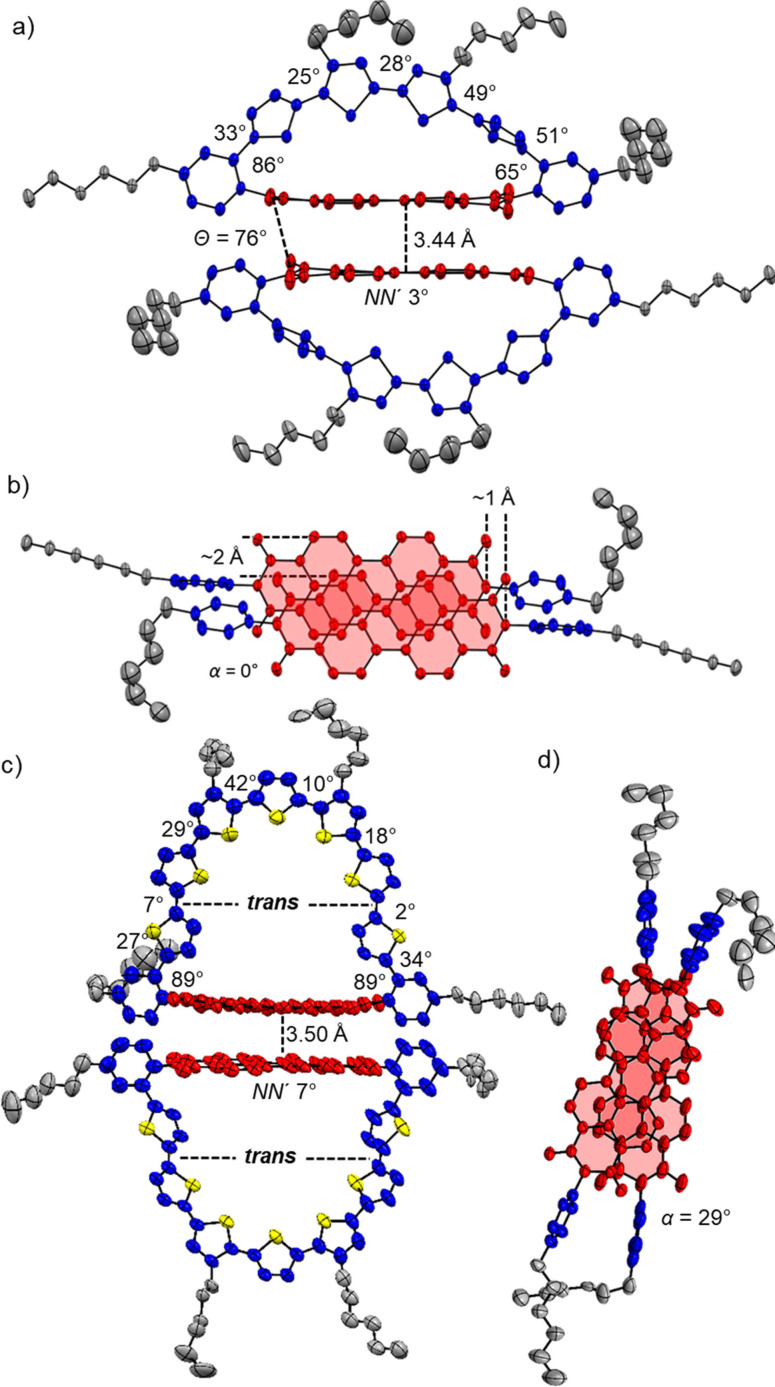
Crystal structure of a)‐b) **4T**‐**PBI** and c)‐d) **7T**‐**PBI** with ellipsoids set to 50 % and 15 % probability, respectively.^[21]^ a)/c) Side view onto the dimer structures of **4T**‐**PBI** and **7T**‐**PBI**. b)/d) Top view onto the π‐stacked chromophore arrangement of **4T**‐**PBI** and **7T**‐**PBI** within the dimer while omitting the thiophene bridge. The hydrogen atoms, solvent molecules as well as the disorder of the alkyl chains are omitted for clarity.

In comparison to the corresponding reference oligothiophene compounds **4T**‐**7T** ^[19]^ where rotamer mixtures prevail in solution, the oligothiophene donor chains within the macrocyclic architectures **4T**‐**PBI**, **5T**‐**PBI** ^[16]^ and **6T**‐**PBI** adopt a more static all‐*syn* conformation, as already proven by the crystal structure of the previously reported double oligothiophene‐bridged dyad **(5T)_2_**‐**PBI**.^[16]^ This structural rigidification as well as additional structural features can be investigated by ^1^H NMR spectroscopy (Figure 2). The doublet proton signals of the thiophene closest to the phenyl moiety for **4T**‐**PBI** experience an increased separation also in comparison to those of the linear oligothiophene counterpart **4T** due to a particularly strong upfield shift of the proton localized atop the PBI's aromatic shielding cone (Figure S4). This behaviour becomes less pronounced upon incorporation of additional thiophene units in the macrocyclic donor bridges (**5T**‐**PBI** and **6T**‐**PBI**, Figure S5‐S6), indicating less strain^[7]^ and enlarged distances to the PBI. Interestingly, both doublet proton signals for **7T**‐**PBI** undergo a very strong shielding in comparison to the other macrocycles (Figure 2). This corroborates a *trans*‐conformation of these two outer thiophenes towards their neighbour thiophene unit (see below) where both protons are pointing directly into the PBI shielding zone. This observation is confirmed by ROESY NMR spectroscopy, which shows cross signals of one highfield shifted doublet to one PBI proton signal (Figure S8). On the other hand, the chemical shift of the PBI proton signals do not change to a great extend within the series and also compared to **Ref**‐**PBI** (Figure S9). In order to fully unravel the structural features of our new macrocyclic architectures, single crystals of the smallest macrocycle were grown by slow vapour diffusion of *n*‐hexane in a dibromomethane solution of **4T**‐**PBI**. The molecule crystallizes in a *P* $\bar{1}$ space group via the self‐assembly of a close π‐stacked PBI dimer unit where the intermolecular distance between the two PBI planes is 3.44 Å with only marginal longitudinal (∼1 Å) and lateral (∼2 Å) displacement (Figure 3a‐b). A similar supramolecular assembly in low polar solvents could not be observed based on concentration‐ and temperature‐dependent UV/Vis studies within the accessible solvent polarity and concentration range. While the individual thiophene units are tilted to a very high degree (Figure 3a; 25°–51°) with respect to each other in order to accommodate for the induced overall macrocyclic strain of 19.6 kJ mol^−1^ (according to DFT calculations, see Table S3), the PBI displays only a marginal bending of 3° along the *N*,*N*'‐axis in the solid state.

Strikingly, the angles that both imide phenyl units comprise with the PBI segment (86° and 65°) are non‐equivalent due to the arrangement of the molecules in the crystal. The attached hexyl chain on one side is extremely tilted which strongly affects the respective phenyl segment. This one sided twist is merely present in the solid state, whereas based on the ^1^H NMR spectrum (Figure 2) in solution the hexyl chains as well as the oligothiophene backbone do not show any evidence of a symmetry‐breaking effect of the bridge architecture as only three proton signals of the thiophene bridge are observed (Figure S4). However, the fact that one thiophene unit is almost stacked on top of the PBI π‐plane seems to display the situation more realistically in solution where a strong shielding of the thiophene proton doublets in ^1^H NMR compared to the other macrocycles **5T**‐**PBI** and **6T**‐**PBI** was observed (Figure 2). Single crystals of the largest macrocycle could be obtained by slow vapour diffusion of methanol in a chlorobenzene solution of **7T**‐**PBI**. This crystal structure likewise reveals a stacked dimer structure, however with a rotationally displaced arrangement of the two PBI (α=29°, Figure 3c–d). Furthermore, the thiophene moiety next to the phenyl unit is rotated into a *trans* like arrangement to the neighbouring thiophenes as already noted in solution by the strong upfield shift of both thiophene doublets in the ^1^H NMR of about 0.5 ppm (compared to **6T**‐**PBI**, Figure 2), due to a different chemical environment as for the other protons in the bridge (see discussion above). This *trans‐*twist of two thiophenes leads to a significant structural relaxation as the calculated strain energies for **7T**‐**PBI** (22.5 kJ mol^−1^) is reduced by almost one third in comparison to a theoretical all‐*syn* analogue **7T**‐**PBI**‐**T** (30.6 kJ mol^−1^, for details see Supporting Information). It should be noted at this point that the PBI subunit contortion is highly overestimated by the DFT calculation especially for **4T**‐**PBI** (Figure S17). The induced macrocyclic strain is most likely almost exclusively compensated by the respective oligothiophene bridges in all macrocycles as the PBI proton signals remain mainly unaffected.

### Redox properties

**Figure 4:**
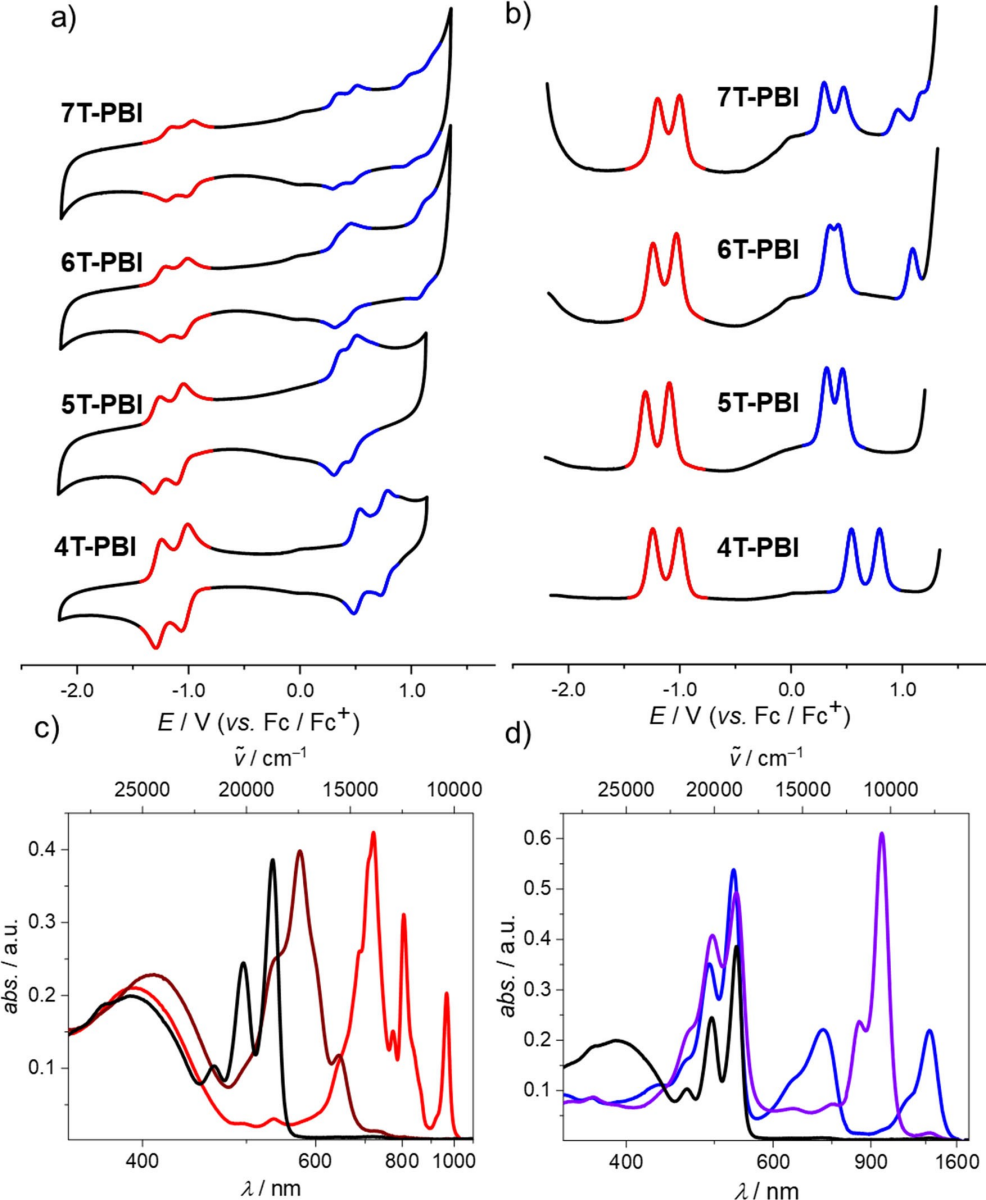
a) CV and b) DPV measurements of **4T**‐**PBI**, **5T**‐**PBI** ^[16]^, **6T**‐**PBI** and **7T**‐**PBI** (from bottom to top) as well as c) UV/Vis/NIR absorption spectra of **4T**‐**PBI** (black line) upon electrochemical reduction to **4T**‐**PBI** ^.−^ (red line), **4T**‐**PBI** ^2**<**M‐>^ (maroon line) and d) electrochemical oxidation to **4T** ^.**+**^‐**PBI** (blue line) and **4T^2+^**‐**PBI** (violet line). All measurements were carried out in CH_2_Cl_2_ solutions with Bu_4_NPF_6_ at room temperature (*c*_0_=10^−4^ M).

The complete series of oligothiophene‐bridged macrocycles as well as most of the reference compounds were electrochemically characterized by cyclic voltammetry (CV), differential pulse voltammetry (DPV) as well as spectroelectrochemistry (SEC, Figure S10‐S13) in CH_2_Cl_2_ with tetrabutylammonium hexafluorophosphate (Bu_4_NPF_6_) as supporting electrolyte. In the CV (Figure 4a) reversibility of all involved redox processes can be monitored. Two reversible reduction waves corresponding to the anionic and dianionic PBI chromophore at around −1.00 V (*E*_red,1_) and −1.20 V (*E*_red,2_) were detected for all macrocycles (Table 1), which are in very good agreement to **Ref**‐**PBI** (−0.99 V/−1.19 V).^[16]^ In contrast, the oxidation events differ greatly within the series. Thus, whilst the first one‐electron oxidation process is high for the smallest macrocycle **4T**‐**PBI** (*E*_ox,1_=+0.54 V, Table 1) the oxidation of the other macrocycles **5T**‐**PBI** ^[16]^ up to **7T**‐**PBI** can be accomplished at remarkably lower oxidation potentials of only *E*_ox,1_=+0.32 V to +0.38 V. Furthermore, in these rigid macrocyclic bridges the required energies for cation formation are disparately higher in the case of **4T**‐**PBI** (0.54 V) and **6T**‐**PBI** (*E*_ox,1_= 0.38 V) compared to linear analogues **4T** (*E*_ox,1_=+0.33 V) and **6T** (*E*_ox,1_=+0.16 V). Due to the special aliphatic decoration and the concomitant cation destabilization in the linear oligothiophenes **5T** (*E*_ox,1_=+0.41 V) and **7T** (*E*_ox,1_=+0.24 V), which was described previously^[19]^ the mentioned potential difference is non‐existent for **5T**‐**PBI** (*E*_ox,1_=+0.34 V) in all‐*cis* configuration and less pronounced for **7T**‐**PBI** (*E*_ox,1_= +0.32 V).

**Table 1:** Electrochemical properties of macrocycles **4T**‐**PBI**, **5T**‐**PBI**,^[16]^ **6T**‐**PBI** and **7T**‐**PBI** in CH_2_Cl_2_ at room temperature (*c*_0_=10^−4^ M).^[a]^

	**4T‐PBI**	**5T‐PBI** ^[16]^	**6T‐PBI**	**7T‐PBI**
*E*_ox,1_ [V]	+0.54	+0.34	+0.38	+0.32
*E*_ox,2_ [V]	+0.80	+0.48	+0.46	+0.50
*E*_ox,3_ [V]	–	–	+1.12	+0.98
*E*_ox,4_ [V]	–	–	–	+1.19
*E*_ox,2_−*E*_ox,1_ [V]	0.26	0.14	0.08	0.18
*E*_red,1_ [V]	−0.99	−1.07	−1.00	−0.98
*E*_red,2_ [V]	−1.24	−1.29	−1.21	−1.18

[a] Bu_4_NPF_6_ was used as electrolyte and all half‐wave potentials are referenced against the ferrocenium/ferrocene (Fc^+^/Fc) redox couple.

Even greater differences within the macrocyclic series can be observed by comparing the second oxidation potentials of **4T**‐**PBI** (*E*_ox,2_= 0.80 V) with the other macrocycles **5T**‐**PBI** ^[16]^ to **7T**‐**PBI** (*E*_ox,2_=+0.46 V to +0.50 V). Here, especially the potential difference (*E*_ox,2_–*E*_ox,1_, Table 1) between the cationic and dicationic states shall be highlighted, which is best viewed in the DPV spectra with a higher resolution of the individual waves (Figure 4b). This potential difference is largest in **4T**‐**PBI** (0.26 V) and smallest in **6T**‐**PBI** (0.08 V) indicating a highly stabilized dicationic state in the latter case.^[22]^ Presumably owing to the aforementioned *trans* orientation of two thiophenes in **7T**‐**PBI** the potential difference almost resembles the one observed for the free **7T** with 0.19 V.^[19]^ Notably, **6T**‐**PBI** and **7T**‐**PBI** show more than two reversible oxidation processes within the CH_2_Cl_2_ solvent window which is in accordance with observations made for the linear oligothiophenes **6T** and **7T**, where also the formation of tri‐and tetracationic species could be observed.^[19]^

**Figure 5:**
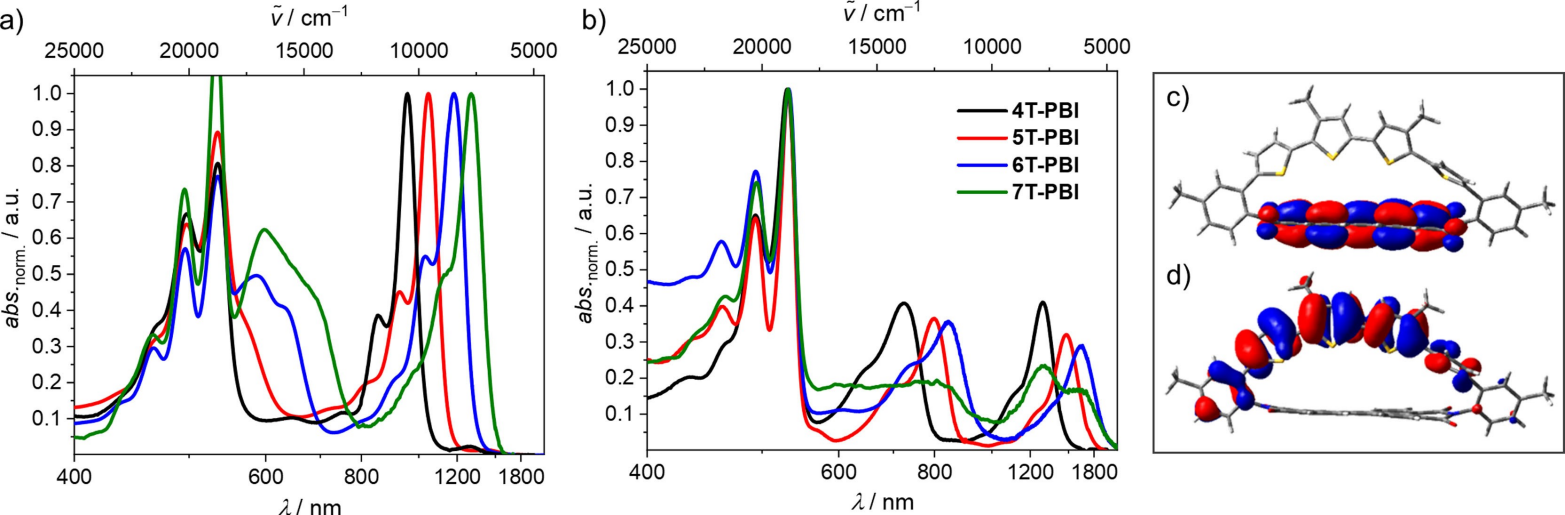
Normalized UV/Vis/NIR absorption spectra of the a) cations and b) dications of **4T**‐**PBI** (black), **5T**‐**PBI** ^[16]^ (red), **6T**‐**PBI** (blue) and **7T**‐**PBI** (green), respectively. All measurements were carried out in CH_2_Cl_2_ solutions with Bu_4_NPF_6_ at room temperature (*c*_0_=10^−4^ M). c) LUMO and d) HOMO of **4T**‐**PBI** based on the molecular geometry received from X‐ray analysis. The hexyl chains were omitted for clarity. The quantum mechanics calculations were carried out on the level of B3LYP density functional with the 6–31G(d) basis set as implemented in Gaussian 16.

In order to unravel the electronic nature of the macrocycles upon electrochemical reduction and oxidation, SEC measurements of the series of macrocycles were conducted in CH_2_Cl_2_ (Figure 4c, d/ Figure 5a, b). Reduction of **4T**‐**PBI** (Figure 4c, black line) revealed typical spectral signatures of a PBI radical anion (PBI^.−^, red line) comprising three clearly distinguishable bands at 720 nm, 804 nm and 964 nm corresponding to **4T**‐**PBI** ^.−^ (red line) which decrease upon further reduction and give rise to a new absorption band at 573 nm belonging to the dianion **4T**‐**PBI^2^** ^−^ (maroon line).^[23]^ Spectral shapes of anion and dianion do not change to a great extent within the series of macrocycles because of the decoupled states between D and A as well as the almost identical reduction potentials observed in the CV (Figure S10a, b to Figure S13a, b). However, single oxidation of **4T**‐**PBI** leads to the simultaneous formation of two absorption bands at 723 nm and 1285 nm, indicating the formation of the cationic species **4T** ^.**+**^‐**PBI** (Figure 4d, blue line). The lack of one thiophene moiety in the bridge causes a hypsochromic shift of 72 nm (1252 cm^−1^) and 195 nm (1025 cm^−1^) for both maxima, respectively, in comparison to **5T**‐**PBI** oxidation (Figure 5a). As expected, within the macrocycle series up to **6T**‐**PBI** these cation species spectra undergo a broadening as well as a bathochromic shift to 840 nm and 1633 nm, respectively (Figure 5a). Further oxidation to the dicationic species **4T^2+^**‐**PBI** (purple line) produces a strong absorption band at 953 nm (Figure 4d) which is shifted bathochromically for **5T^2+^**‐**PBI** ^[16]^ (Figure 5b). The appearance of one single peak in the dicationic state for **4T^2+^**‐**PBI** and **5T^2+^**‐**PBI** indicates the formation of a bipolaron (spinless dication) species causing minimal structural distortion of the bridge.^[24]^ Interestingly, in the cases of **6T^2+^**‐**PBI** and **7T^2+^**‐**PBI** the rise of a second band at 585 and 598 nm with a higher intensity with regard to the low energy band for the latter species can be monitored, respectively (Figure 5b, Figure S14). Presumably in these cases, a growing percentage of a polaron pair configuration with more structural contortion is formed as already described for a larger cyclic oligothiophene of ten subunits by Bäuerle and co‐workers.^[24]^ The polaron‐pair state indicates localization of the charges in separated distorted segments of the sufficiently long oligothiophene π‐system.

The localization and shapes of the lowest unoccupied and highest occupied molecular orbitals (LUMO/HOMO) of the neutral **4T**‐**PBI** (Figure 5c/d) suggest as expected very little electronic communication between the electron‐rich oligothiophene chain and electron‐poor PBI acceptor segment. This idea is supported for all macrocycles by time‐dependent density functional theory (TDDFT) calculations (Table S4), where the oscillator strengths for HOMO‐LUMO transitions were determined to be negligible, which agrees well with the observed UV/Vis absorption spectra (see below), that show no indication for any charge transfer (CT) band. Optimized molecular geometries in the gas phase and respective HOMOs and LUMOs for **4T**‐**PBI** to **7T**‐**PBI** can be found in the Supporting Information (Figure S18), which display all the same behaviour as discussed for **4T**‐**PBI**.

### Steady‐state optical properties

**Figure 6:**
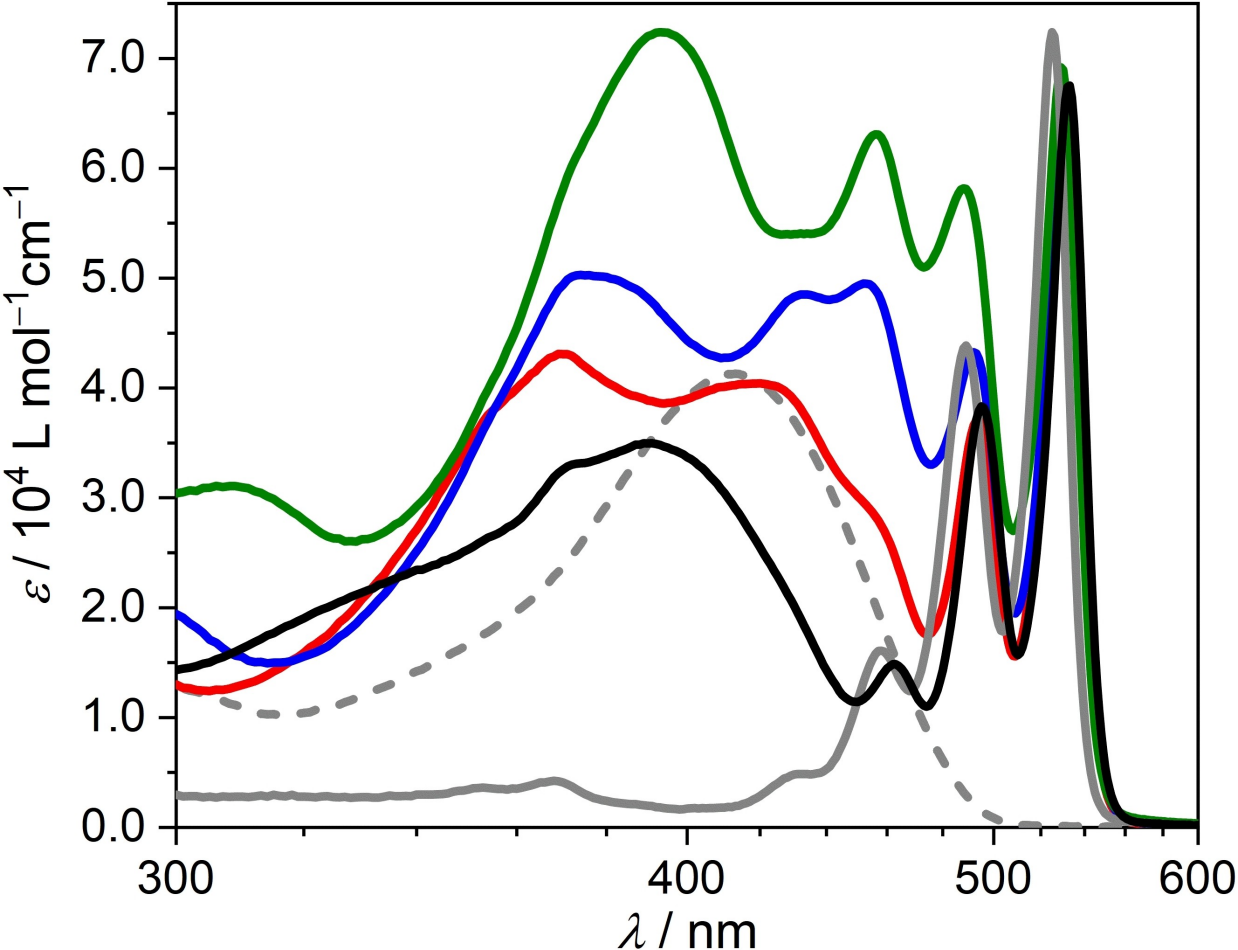
UV/Vis spectra of **4T**‐**PBI** (black), **5T**‐**PBI** (red),^[16]^ **6T**‐**PBI** (blue), **7T**‐**PBI** (green), **Ref**‐**PBI** (solid grey) and **4T** (dashed grey)^[19]^ in CH_2_Cl_2_ at room temperature (*c*_0_=10^−5^ M).

UV/Vis spectroscopic investigations of the macrocycles **4T**‐**PBI** to **7T**‐**PBI** and **Ref**‐**PBI** ^[16]^ were performed in CH_2_Cl_2_ (Figure 6). In comparison to the **Ref**‐**PBI** with an absorption maximum (*λ*_abs,max_) at 526 nm, all absorption maxima of the macrocycles exhibit a slight bathochromic shift of the PBI's S_0_‐S_1_ absorption band (529‐533 nm, Table 2) by about 3 nm to 7 nm (107 cm^−1^ to 249 cm^−1^) as well as a pronounced additional absorption band below 450 nm that can be attributed to their oligothiophene bridges. In comparison to the structureless π‐π* transition of their respective linear counterparts **4T**‐**7T**,^[19]^ rigidification of the oligothiophene bridge within the macrocyclic arrangements leads to structured absorption spectra of **4T**‐**PBI** to **7T**‐**PBI** below 450 nm, which partially superimposes with the PBI's S_0_‐S_1_ transition (Figure 6). Unlike other PBI‐oligothiophene dyad systems[^14a^, ^14b^] the UV/Vis spectra of **4T**‐**PBI** to **7T**‐**PBI** are not simply a summation of the reference donor and acceptor spectrum. Depending on the donor bridge size, different dihedral angles and cisoid/transoid arrangements between the thiophene units lead to distinct conjugation lengths in each oligothiophene bridge. Concomitantly, partial breaking of conjugation may cause band splitting in the absorption spectra which is most pronounced in case of **6T**‐**PBI**. With increasing oligothiophene length the corresponding lowest energy absorption band of the oligothiophene bridges undergo a bathochromic shift from 390 nm (**4T**‐**PBI**) up to approximately 455 nm (**7T**‐**PBI**). Thus, the degree of spectral overlap with the PBI's S_0_‐S_1_ transition becomes more and more pronounced. While the spectral regions of oligothiophene and PBI absorption in the case of **4T**‐**PBI** are clearly distinguishable from each other, the absorption bands of the oligothiophene subunit in **7T**‐**PBI** overlap strongly with the vibronic A_0‐1_ and A_0‐2_ transitions of the PBI. Accordingly, despite of the still pronounced A_0‐0_ band of PBI the lowest energy optically excited state may be located at the oligothiophene subunit for the longest bridge, in particular if we take the increased structural relaxation of oligothiophenes in their excited state into account.^[25]^

**Table 2:** Photophysical properties of macrocycles **4T**‐**PBI**, **5T**‐**PBI**,^[16]^ **6T**‐**PBI** and **7T**‐**PBI** in solution (CH_2_Cl_2_ and cyclohexane) at room temperature.

	**4T‐PBI**	**5T‐PBI** ^[16]^	**6T‐PBI**	**7T‐PBI**
*λ*_abs,max_^[a]^ [nm]	533	531	531	529
*ϵ*_max_^[a]^ [10^3^ M^−1^ cm^−1^]	67.5	64.8	67.2	69.2
*λ*_abs,max_^[b]^ [nm]	522	519	519	564
*λ*_em,max_^[b,c]^ [nm]	530	523	533	527
*λ*_em,max_^[b,d]^ [nm]	530	523	527	527
$\Delta {\tilde{\nu }}_{\mathrm{Stokes}}$ ^[b]^ [cm^−1^]	218	329	220	221
*Φ*_fl_^[b], [e]^ [%]	<1.0	<1.0	<1.0	<1.0

[a] CH_2_Cl_2_, *c*_0_=10^−5^ M. [b] Cyclohexane, *c*_0_=10^−7^ M. [c] *λ*_ex_=350 nm. [d] *λ*_ex_=480 nm. [e] The fluorescence quantum yields of the PBI subunit were measured relative to *N,N*′‐bis(2,6‐diisopropylphenyl)‐1,6,7,12‐tetraphenoxy‐perylene‐3,4 : 9,10‐bis(dicarboximide) as a reference^[27]^ at four different excitation wavelengths in the spectral region of the PBI subunit.

As has often been observed for donor‐acceptor dyads, no fluorescence could be detected in solvents with higher polarity such as CH_2_Cl_2_ or CHCl_3_, whereas **Ref**‐**PBI** exhibits a quantum yield (*Φ*_fl_) close to unity.^[26]^ The electron donating oligothiophene subunit almost quantitatively quenches the PBI emission via photoinduced electron transfer (PET) resulting in *Φ*_fl_ values of less than 1 % (Table 2) within the macrocycle series even in low polar cyclohexane. Also in cyclohexane, structured absorption spectra of the macrocycles (Figure S15, black solid lines) comprise features from both, the oligothiophene donor bridge at lower and the acceptor PBI at higher wavelengths. As the macrocyclic architectures **4T**‐**PBI** to **7T**‐**PBI** are composed of spectrally distinguishable oligothiophene and PBI subunits, we investigated the emission features of these systems upon excitation at two different wavelengths (*λ*_ex_) at 350 nm and 480 nm. While for *λ*_ex_ = 480 nm primarily the PBI acceptor is excited, at 350 nm mainly the oligothiophene donor is excited. For a full comparison, the emission spectra of non‐cyclic reference compounds **4T**‐**7T** can be found in the Supporting Information (Figure S16). Upon almost exclusive excitation of the oligothiophene unit of the macrocycles **4T**‐**PBI** to **6T**‐**PBI** at 350 nm (Figure S15, maroon solid lines), Förster Resonance Energy Transfer (FRET) from donor to acceptor moiety occurs and predominantly PBI emission can be detected, as indicated by the characteristic vibronic emission profile of the PBI. Thus, these fluorescence spectra almost resemble those recorded upon selective excitation of the PBI at 480 nm (Figure S15, red solid lines). In contrast, upon oligothiophene excitation of the longer analogue in **7T**‐**PBI** the FRET proceeds back and forth between donor and acceptor unit which may be due to an energetically low lying oligothiophene S_1_ state of the oligothiophene (see above) and thus a superimposed emission of donor and acceptor can be monitored. This observation is confirmed by excitation at *λ*_ex_= 480 nm which leads to an emission spectrum, where both subunits seem to contribute.

### Time‐resolved transient absorption

**Figure 7:**
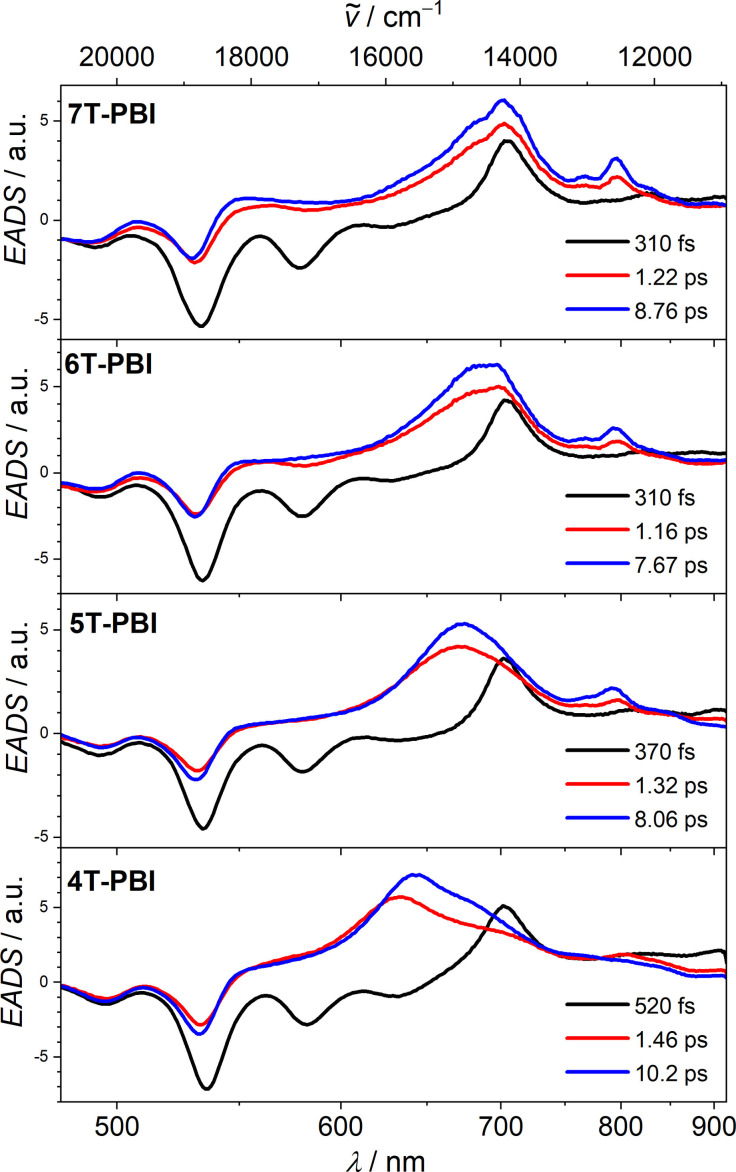
EADS and respective lifetimes from a global fit analysis of the transient spectra of macrocycles **4T**‐**PBI**, **5T**‐**PBI**,^[16]^ **6T**‐**PBI** and **7T**‐**PBI** obtained by excitation at 530 nm in CH_2_Cl_2_ (*c*_0_=10^−4^ M).

For a better understanding of the fluorescence quenching process in the four macrocycles **4T**‐**PBI** to **7T**‐**PBI**, transient absorption (TA) spectroscopy was performed in CH_2_Cl_2_ solutions at 530 nm excitation with ca. 30 fs pulses which populate the S_1_ state of the PBI moiety.^[28]^ TA spectra were obtained by probing the excited states by a delayed white light continuum between 490 nm and 910 nm. The thereby obtained transient spectra (see Figure S19‐S21) were deconvoluted by a global analysis employing a consecutive kinetic model which yielded evolution associated difference spectra (EADS). These EADS are associated with specific lifetimes and are presented for **4T**‐**PBI** to **7T**‐**PBI** in Figure 7. For all macrocycles the first EADS (black spectrum) shows a negative signal at 530 nm which is caused by a ground state bleaching (GSB) of the PBI chromophore. Stimulated emission (SE) of the PBI is observed as a negative signal at 580 nm and also overlapping with the GSB at 530 nm (Figure 7).

The three dyads with more extended oligothiophene chains (**5T**‐**PBI** to **7T**‐**PBI**) exhibit very similar EADS and photoinduced dynamics that will be discussed here in more detail for **7T**‐**PBI**. Thus, the first EADS of **7T**‐**PBI** has a lifetime of 0.3 ps that refers to the S_1_ state of the PBI. It is followed by a second EADS (red spectrum) where the SE is replaced by an excited state absorption (ESA) which extends from ca. 500 nm to 850 nm with maxima at 700 nm and 800 nm. This EADS is very similar to that of PBI radical anion,^[29]^ (also compare blue line in Figure S13a), and proves the formation of a charge separated state (CSS). The lifetime of the foregoing EADS (0.3 ps) thus is limited by the charge separation (CS) process with *k*_CS_=(0.3 ps)^−1^. The following blue EADS is very similar to the red EADS. Therefore, the former is interpreted to be a hot CSS which transforms by molecular and solvent relaxation processes to the cold CSS with *k*=(1.2 ps)^−1^. The lifetime of the cold CSS (8.8 ps) then refers to charge recombination with *k*_CR_=(8.8 ps)^−1^ to the ground state since no other components were found in the analysis of the TA spectra. While the spectral shape of the EADS of the singlet PBI state is the same for all four macrocycles, the rate of CS gradually decreases from (0.3 ps)^−1^ to (0.5 ps)^−1^ within that series. Nevertheless, these CS processes are among the fastest for PBI‐oligothiophene dyads known in literature.[^14a^, ^16^, ^30a^, ^30b^] A TA control experiment with a 1 : 1 mixture of linear **4T** ^[19]^ and **Ref**‐**PBI** ^[16]^ under the same conditions as utilized for the macrocycles reveals exclusive formation of the PBI's S_1_ state with a lifetime of 3.5 ns (Figure S25) which underlines that the fast charge separation process is due to the covalent linkage of the two chromophores in **4T**‐**PBI** to **7T**‐**PBI**.

In a more detailed analysis we note that the spectral shape of the blue EADS changes gradually from **7T**‐**PBI** to **5T**‐**PBI** (Figure 7), that is, the maximum at 700 nm blue‐shifts to 640 nm and a small band at 800 nm decreases in intensity. This is interpreted as a gradual decrease of the extent of charge separation from **7T**‐**PBI** to **5T**‐**PBI**, which is confirmed by a comparison of the respective “cold” EADS with the sum of anion and cation spectra obtained from SEC in Figure S27. Thus, while for **7T**‐**PBI** comparison with aforementioned SEC spectra suggest full CS, for the other dyads the degree of CS is reduced. Most notably, for the smallest oligothiophene bridged **4T**‐**PBI** we interpret the spectra as being due to the formation of a CT state with only partial negative charge (δ^−^) at the PBI and partial positive charge (δ^+^) at the thiophene bridge. Interestingly, the lifetime of the CT state of **4T**‐**PBI** (10.2 ps) is not much different from that of the full CSS of **7T**‐**PBI** (8.8 ps). This shows that in the present cases the spectral signatures of the CS/CT states are much more sensitive to the extent of charge distribution than the kinetics.

Whilst the detection of the PBI radical anion is straightforward due to their intense and characteristic bands in the visible and NIR region,^[31]^ the detection of the corresponding radical cations of oligothiophenes are more challenging. To prove the positive charge residing on the oligothiophene bridge we probed the excited states of **5T**‐**PBI** in the NIR upon excitation at 400 nm (Figure S23, Figure S24b). A broad band between 1200–1450 nm is in very good agreement with SEC measurements of the radical cation species (Figure S26). Thus, while the TA spectra visible range indicates partial negative charge on the PBI moiety (Figure S22, Figure S24a) the NIR part of the TA spectra prove a full positive charge located at the oligothiophene subunit. In other words, the centre of a dipole moment difference vector representing the charge transfer is located nearer to the oligothiophene. In that way, TA spectroscopy in comparison with SEC can give rather detailed insight into the charge distribution of such CT states. In the UV/Vis region, excitation at 400 nm reveals similar EADS spectra for **5T**‐**PBI** (Figure S25a) in comparison to an excitation of the same compound at 530 nm.^[16]^ Here, presumably due to the extremely close proximity of donor and acceptor an unresolvable fast energy transfer from the oligothiophene bridge to the PBI chromophore in the lower femtosecond region occurs. Thus, independent of oligothiophene or PBI excitation wavelength (400 nm or 530 nm) the TA spectra remain similar.

## Conclusion

In this study we synthesized a series of novel macrocyclic perylene bisimide dyads comprising quarter‐, quinque‐, sexi‐ and septithiophene bridges that connect both imide positions. The final macrocyclization reaction was realized by applying a three step Pt‐catalyzed cross coupling procedure. These unique molecular architectures containing a planar PBI acceptor and tether‐like donor moieties, which are electronically independent due to their connectivity, were compared to their corresponding linear reference subunit systems revealing differences regarding absorption, emission and electrochemical properties. X‐ray analysis in line with NMR spectroscopy provided valuable structural insights into the smallest and largest macrocycle showing large torsional angles among the thiophene rings and a dimer arrangement with π‐π‐stacked PBI subunits in the solid state. Due to a highly efficient photoinduced electron transfer process these donor‐acceptor systems are almost non‐fluorescent even in low polar solvents such as cyclohexane. According to the spectral signatures in the excited state investigated by transient absorption spectroscopy, the larger oligothiophene bridge sizes support full charge separation into oligothiophene radical cations and PBI radical anions whilst a charge transfer state with incomplete charge separation is observed for the dyad with the smallest quarterthiophene bridge.

## Conflict of interest

The authors declare no conflict of interest.

1

## Supporting information

As a service to our authors and readers, this journal provides supporting information supplied by the authors. Such materials are peer reviewed and may be re‐organized for online delivery, but are not copy‐edited or typeset. Technical support issues arising from supporting information (other than missing files) should be addressed to the authors.

Supporting Information

## Data Availability

The data that support the findings of this study are available from the corresponding author upon reasonable request.
