# Supplementary material for: Macrocyclic Donor‐Acceptor Dyads Composed of Oligothiophene Half‐Cycles and Perylene Bisimides
Source: Chemistry. 2022 Apr 12;28(30):e202200355. doi: 10.1002/chem.202200355 (PMC9323445; doi:10.1002/chem.202200355)
Supplement: Supplementary file 1 — Supporting Information [file CHEM-28-0-s001.pdf]

Chemistry–A European Journal

Supporting Information

Macrocyclic Donor-Acceptor Dyads Composed of Oligothiophene Half-Cycles and Perylene Bisimides

Kevin Bold, Matthias Stolte, Kazutaka Shoyama, Ana-Maria Krause, Alexander Schmiedel, Marco Holzapfel, Christoph Lambert,* and Frank Würthner*

Table of Contents

Experimental Section S3

Synthetic Procedure..S8

Possible Reaction Mechanism for the Macrocyclization..............................S11

Single Crystal X-ray Analysis S13

^{1}H NMR Spectra Comparison S16

Spectroelectrochemistry...S19

Optical Properties in Cyclohexane S23

DFT and TDDFT calculations...S24

Transient Absorption Spectra...S26

NMR Spectra...S30

Mass Spectra S33

Cartesian Coordinates Received from DFT Calculations S36

Supporting References S47

Experimental Section

General methods

All reactions were performed in standard glass equipment. All used chemicals were purchased from commercial suppliers (*abcr/carbolution chemicals*, *Acros Organics*, *Alfa Asear*, *Merck*, *Sigma Aldrich*, *TCI* and *VWR*) and applied without further purification. The synthesis of stannylated oligothiophenes **4**[S1], **5**[S2] and **6**[S3] is literature known. The preparation of compound **3** and **5T-PBI**[S4] as well as reference oligothiophenes **4T**-**7T** and stannylated compound **7**[S5] were reported recently.

THF, CH_2Cl_2, and toluene were purified and dried with the commercial purification system PureSolv MD from *Innovative Technology*. The solvent *m*-xylene was dried over molecular sieves prior to use.

Flash column chromatography was performed on a PuriFLash XS-420 from *Interchim* using columns of the size 0025. The purification by gel permeation chromatography was performed on a *Shimadzu* (LC-20AD Prominence Pump, SPD-MA20A Prominence Diode Array Detector) with two preparative columns (*Japan Analytical Industries Co*., *Ltd)*. Ethanol stabilised $CHCl_3$ (Chromasolv®, *Sigma Aldrich*) was used as eluent.

MALDI-TOF mass spectra were measured with an ultrafleXtreme from *Bruker Daltonics GmbH* using *trans*-2-[3-(4-*tert*-butylphenyl)-2-methyl-2-propenylidene]malono-nitrile (DCTB) as a matrix material.

^{1}H and ^{13}C NMR spectra were recorded on a *Bruker* Avance III HD 400 MHz using deuterated solvents. ^{13}C NMR spectra are broad band proton decoupled. Chemical shifts (δ) are listed in parts per million (ppm) and are reported relative to tetramethyl silane (TMS). Coupling constants (J) are stated in Hertz (Hz). The spectra are referenced internally to residual proton solvent resonances or natural abundance carbon resonances. Multiplicities are reported as s = singlet, brs = broad singlet, d = doublet, dd = doublet of doublets, t = triplet, dt = doublet of triplets, q = quartet, quin = quintet, sex = sextet, m = multiplet with the chemical shift in the center of the signal.

UV/Vis absorption spectra were recorded with solutions of the compounds in cuvettes (SUPRASIL®, *Hellma® Analytics*) on a Jasco V-670 or V-770 spectrometer and fluorescence spectra on a FLS980-D2D2-ST fluorescence spectrometer (*Edinburgh Instruments*) and were corrected against the photomultiplier sensitivity and the lamp intensity.

Spectroelectrochemical experiments were performed on a Cary 5000 UV/Vis/NIR spectrometer from *Agilent* in combination with a sample compartment consisting of a custom-made cylindrical PTFE cell with a sapphire window and an adjustable three in one electrode (6 mm platinum disc working electrode, 1 mm platinum counter and Ag/AgCl leak free reference electrode) in reflection mode. The optical path was adjusted to 100 µm with a micrometer screw. Potentials were applied with a reference potentiostat PAR 283 from *Princeton Applied Research.* Upon applying a new potential to the solution an equilibration time of 20 seconds between each measurement was employed.

The transient absorption spectrometer setup for the excitation at 530 nm is based on a femtosecond laser "Solstice" from *Newport-Spectra Physics* with a fundamental wavelength of 800 nm which provides 100 fs long pulses with a repetition rate of 1 kHz. This laser source was used to pump a NOPA to generate the excitation pulses at 530 nm with a pulse length <50 fs. The FWHM-bandwidth of the excitation pulse was 8.5 nm and the pulse energy was set to 20 nJ. Wire grid polarizers were used to set the pump pulse polarization to 54.7° in relation to the horizontal polarized white light continuum to achieve magic angle conditions. Another part of the laser beam was guided to a TOPAS-C from *Light-Conversion* to obtain a wavelength from 1000 nm, which was used to generate the probing white light continuum within a sapphire crystal. To achieve the probe range from 455 nm to 915 nm a dielectrically coated quartz glass short pass filter with 950 nm, thickness 3 mm, from *Edmund-Optics* were used. The sample was dissolved in spectroscopic grade CH_2Cl_2 from ACROS organics and the solution was filled in a quartz glass cuvette with an optical path length of 2 mm. The optical density at the excitation wavelength was set to 0.34 for **4T-PBI**, 0.50 for **5T-PBI**[S4], 0.32 for **6T-PBI** and 0.30 for **7T-PBI**. The IRF was 60-100 fs as

measured for stimulated Raman signals of the solvent. Further details on this spectrometer setup are provided in ref. [S6].

For the transient absorption spectrometer setup excitation at 400 nm the same laser source was used to pump a TOPAS to generate the excitation pulses at 400 nm with a pulse length <150 fs. The FWHM-bandwidth of the excitation pulse was 1.7 nm and the pulse energy was set to 20 nJ. Wire grid polarizers were used to set the pump pulse polarization to 54.7° in relation to the horizontal polarized white light continuum to achieve magic angle conditions. Another part of the laser beam was guided to a TOPAS-C from *Light-Conversion* to obtain a wavelength from 800 nm, which was used to generate the probing white light continuum within a 15 mm sapphire crystal. To achieve the probe range from 771 nm to 1501 nm a coloured glass filter with 850 nm from *Edmund-Optics* were used. The sample was dissolved in spectroscopic grade CH_2Cl_2 from ACROS organics and the solution was filled in a quartz glass cuvette with an optical path length of 0.2 mm. The optical density at the excitation wavelength was set to 0.34 for **5T-PBI**. The IRF was >50 fs as measured for stimulated Raman signals of the solvent.

Cyclic and differential pulse voltammetry experiments were carried out with a *BASi* Epsilon potentiostat connected to a microcell apparatus from *rhd instruments* involving a 1.6 mL sample container, a platinum counter- and pseudo-reference electrode as well as a glassy carbon working electrode. The compounds were dissolved in CH_2Cl_2 and Bu_4NPF_6 was added as supporting electrolyte.

Single crystal X-ray diffraction data of **4T-PBI** were collected at 100 K on a Bruker D8 Quest Kappa Diffractometer using CuK_α-radiation (λ = 1.54178 Å) from an Incoatec IµS microsource with Montel multi layered mirror with a Photon II CPAD detector. The structure was solved using direct methods, expanded with Fourier techniques and refined with the SHELX software package.[S7] All non-hydrogen atoms were refined anisotropically. Hydrogen atoms were included in the structure factor calculation on geometrically idealized positions. The compound **4T-PBI** crystallizes as red plates that scatter very weakly. Multiple attempts were made to grow better diffracting crystals. Crystals that are better suitable for single X-Ray diffraction studies were grown from

dibromomethane/*n*-hexane. However, the data yielded problems due to weak diffraction and disorder in the atom position of the side-chains and of the solvent. A large amount of disorder in the side chains and in the solvent was observed which made it difficult to model the chains and the solvent. Therefore restrains were applied to the atoms in the disorder. Alerts originated from the large amount of disorder in the side chains and in the solvent.

Single crystal X-ray diffraction data of **7T-PBI** were collected at the P11 beamline at DESY. The diffraction data were collected by a single 360° scan ϕ sweep at 100 K. The diffraction data were indexed, integrated, and scaled using the XDS program package.[S8] The structures were solved using SHELXT,[S9] expanded with Fourier techniques and refined using the SHELX software package.[S10] Hydrogen atoms were assigned at idealized positions and were included in the calculation of structure factors. All non-hydrogen atoms in the main residue were refined anisotropically. The diffraction data had a resolution of ca. 1.5 Å (defined by the resolution shell with intensity $I > 2\sigma$). For refinement diffraction data until 1.1 Å were used. In the crystal structure, the four crystallographic isomers found in the asymmetric unit cell were restrained by the SAME command of SHELX due to low resolution of diffraction data. All the alkyl substituents had heavy disorder and were modelled with constraints and restraints using standard SHELX commands RIGU, DELU, ISOR, SADI, DFIX, DANG, EADP, FLAT, CHIV, and SIMU. A similar set of commands were used to model solvents found in solvent accessible voids.

The diffraction of this crystal was not satisfactory for normal criteria of small molecule crystallography. This is due to the large molecular structure and thus large unit cell similar to those of macromolecular crystals. Nevertheless, diffraction data were good enough to elucidate the packing motif of this molecule and therefore solved with a resolution of ca. 1.6 Å. Diffraction data down to 1.1 Å were used for refinement and this treatment caused some A-level Alerts in the Checkcif routine. Due to cutoff of diffractions below 1.1 Å the data / parameter ratio needed to as low as 5.62. The weighted R factor (wR_2) was high (0.47) due to inclusion of weakly diffracting data between 1.6 and 1.1 Å. Due to the low data / parameter ratio and disorder of solvent

molecules (chlorobenzene and methanol) the shift/error during the last step of refinement was as large as 0.65.

DFT and TD-DFT calculations were performed by Gaussian 16[S11] using B3LYP/6-31G(d) level of theory.

Synthetic Procedure

Typical procedure for macrocyclization reactions

A stirring solution of Pt-complex **3** (50.0 mg, 32.3 μmol, 1.0 eq.) in degassed toluene (40 mL) was stirred and stannylated oligothiophene compounds (35.5 μmol, 1.1 eq.) in degassed toluene (1.0 mL) were added dropwise *via* a syringe pump over 15 h. Then the reaction mixture was stirred overnight at 75 °C. The solvent was removed *in vacuo* and the crude residue was washed with *n*-hexane. The crude product was redissolved in degassed CH_2Cl_2 (40 mL) and 1,1'-bis(diphenylphosphino)ferrocene (39.4 mg, 71.0 μmol, 2.2 eq.) was added. The solution was stirred for 6 h at room temperature. The solvent was removed *in vacuo* and the residue was redissolved in degassed *m*-xylene (40 mL) and stirred overnight at 120 °C. The solvent was removed under reduced pressure and the crude product was purified *via* flash column chromatography (CH_2Cl_2/*n*-hexane = 1:1 to 1:0) and gel permeation chromatography ($CHCl_3$) to give the desired compounds.

4T-PBI

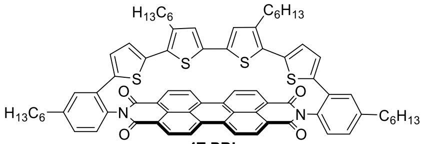

4T-PBI

Yield: 5.29 mg, 4.39 μmol, 14%, red solid. **^{1}H NMR** (400 MHz, CD_2Cl_2): δ/ppm = 8.68 (d, ^3J = 8.2 Hz, 4H), 8.64 (d, ^3J = 7.9 Hz, 4H), 7.69 (d, ^4J = 1.5 Hz, 2H), 7.51 (d, ^3J = 8.0 Hz, 2H), 7.47 (dd, ^3J = 8.0 Hz, ^4J = 1.9 Hz, 2H), 7.25 (d, ^3J = 3.8 Hz, 2H), 6.88 (d, ^3J = 3.8 Hz, 2H), 6.88 (s, 2H), 2.85 (t, ^3J = 7.7 Hz, 4H), 2.48 (t, ^3J = 7.7 Hz, 4H), 1.83 (quin, ^3J = 7.3 Hz, 4H), 1.22-1.51 (m, 28H), 0.98 (t, ^3J = 7.1 Hz, 6H), 0.87 (t, ^3J = 6.8 Hz, 6H). **^{13}C NMR** (101 MHz, CD_2Cl_2): δ/ppm = 164.3, 145.1, 142.0, 139.3, 135.8, 135.4, 132.2, 131.8, 130.1, 129.7, 129.2, 128.3, 127.0, 126.9, 126.8, 124.0, 132.8, 36.2, 32.2, 31.9, 31.8, 30.8, 29.6, 29.4, 29.2, 23.0, 22.9, 14.3, 14.2. **HRMS** (MALDI-TOF, positive mode, DCTB in $CHCl_3$): *m/z* calculated for $C_{76}H_{72}N_2O_4S_4$ $[M]^+$: 1204.4375, found: 1204.4369. **UV/Vis** λ_{max} (ε_{max}): CH_2Cl_2: 533 nm (67.5 × 10^3 L mol^{-1} cm^{-1}). **Fluorescence** λ_{max} (λ_{ex}): Cyclohexane: 530 nm (480 nm), 530 nm (400 nm). **PLQY**: Φ_{fl} = <1%. **R$_f$**: 0.22 using CH_2Cl_2 as eluent.

6T-PBI

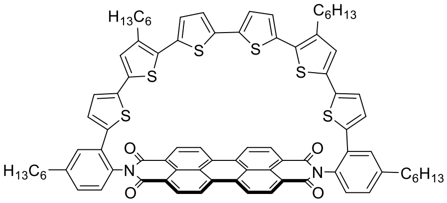

6T-PBI

Yield: 5.85 mg, 4.27 μmol, 13%, orange solid. **^{1}H NMR** (400 MHz, CD_2Cl_2): δ/ppm = 8.79 (d, ^3J = 8.21 Hz, 4H), 8.74 (d, ^3J = 7.9 Hz, 4H), 7.74 (d, ^4J = 1.9 Hz, 2H), 7.41 (d, ^3J = 4.0 Hz, 2H), 7.34 (dd, ^3J = 8.0 Hz, ^4J = 1.9 Hz, 2H), 7.23 (d, ^3J = 3.9 Hz, 2H), 7.17 (d, ^3J = 8.0 Hz, 2H), 7.14 (d, ^3J = 3.8 Hz, 2H), 7.08 (s, 2H), 6.94 (d, ^3J = 3.8 Hz, 2H), 2.80 (t, ^3J = 7.9 Hz, 4H), 2.70 (t, ^3J = 7.9 Hz, 4H), 1.78 (quin, ^3J = 7.2 Hz, 4H), 1.60 (quin, ^3J = 7.2 Hz, 4H),1.45-1.25 (m, 24H), 0.94 (t, ^3J = 7.1 Hz,

6H), 0.86 (t, ^3J = 7.1 Hz, 6H). **^{13}C NMR** (101 MHz, CD_2Cl_2): δ/ppm = 164.3, 145.0, 141.4, 138.8, 138.1, 136.3, 135.8, 135.5, 135.0, 133.3, 132.3, 130.4, 130.2, 130.1, 129.7, 129.5, 129.4, 127.9, 127.4, 127.3, 126.3, 124.1, 124.1, 124.0, 123.7, 36.1, 32.2, 32.0, 31.7, 30.6, 29.6, 29.5, 23.1, 23.0, 14.3, 14.2. **HRMS** (MALDI-TOF, positive mode, DCTB in $CHCl_3$): *m/z* calculated for $C_{84}H_{76}N_2O_4S_6$ $[M]^+$: 1368.4129, found: 1368.4124. **UV/Vis** λ_{max} (ε_{max}): CH_2Cl_2: 531 nm (67.2×10^3 L mol^{-1} cm^{-1}). **Fluorescence** λ_{max} (λ_{ex}): Cyclohexane: 527 nm (480 nm), 533 nm (400 nm). **PLQY**: Φ_{fl} = <1%. **R$_f$**: 0.51 using CH_2Cl_2 as eluent.

7T-PBI

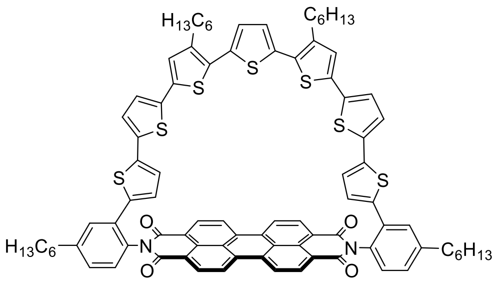

Yield: 1.49 mg, 1.03 μmol, 3%, orange solid. **^{1}H NMR** (600 MHz, CD_2Cl_2): δ/ppm = 8.75 (d, ^3J = 8.4 Hz, 4H), 8.71 (d, ^3J = 7.9 Hz, 4H), 7.54 (d, ^4J = 1.8 Hz, 2H), 7.39 (dd, ^3J = 8.0 Hz, ^4J = 1.9 Hz, 2H), 7.29 (d, ^3J = 8.0 Hz, 1H), 7.10 (d, ^3J = 3.7 Hz, 2H), 7.07 (s, 2H),), 7.06 (d, ^3J = 3.7 Hz, 2H), 7.05 (s, 2H), 6.85 (d, ^3J = 3.8 Hz, 2H), 6.82 (d, ^3J = 3.8 Hz, 2H), 2.80 (t, ^3J = 7.9 Hz, 4H), 2.79 (t, ^3J = 7.9 Hz, 4H), 1.79 (quin, ^3J = 7.6 Hz, 4H), 1.69 (quin, ^3J = 7.6 Hz, 4H), 1.45-1.35 (m, 24H), 0.95 (t, ^3J = 7.4 Hz, 6H), 0.87 (t, ^3J = 7.4 Hz, 6H). **^{13}C NMR** (150 MHz, CD_2Cl_2): δ/ppm = 164.1, 145.2, 140.9, 139.9, 137.4, 136.7, 136.1, 136.0, 135.6, 135.0, 133.1, 132.3, 131.1, 131.0, 130.4, 130.3, 129.9, 129.4, 127.3, 126.2, 126.1, 125.7, 125.2, 125.1, 123.9, 123.7, 123.6, 36.1, 32.2, 32.1, 31.7, 30.5, 30.1, 29.8, 29.6, 23.1, 23.0, 14.3, 14.2. **HRMS** (MALDI-TOF, positive mode, DCTB in $CHCl_3$): *m/z* calculated for $C_{88}H_{78}N_2O_4S_7$ $[M]^+$: 1450.4007, found: 1450.4001. **UV/Vis** λ_{max} (ε_{max}): CH_2Cl_2: 529 nm (69.2×10^3 L mol^{-1} cm^{-1}). **Fluorescence** λ_{max} (λ_{ex}): Cyclohexane: 527 nm (480 nm), 527 nm (400 nm). **PLQY**: Φ_{fl} = <1%. **R$_f$**: 0.30 using CH_2Cl_2 as eluent.

Possible Reaction Mechanism for the Macrocyclization

A possible reaction mechanism starting with Pt-complex **3** for the final macrocyclization reaction is shown exemplarily for **4T-PBI** below (Figure S1). All proposed reaction intermediates (**IN1**-**IN4**) were not isolated.

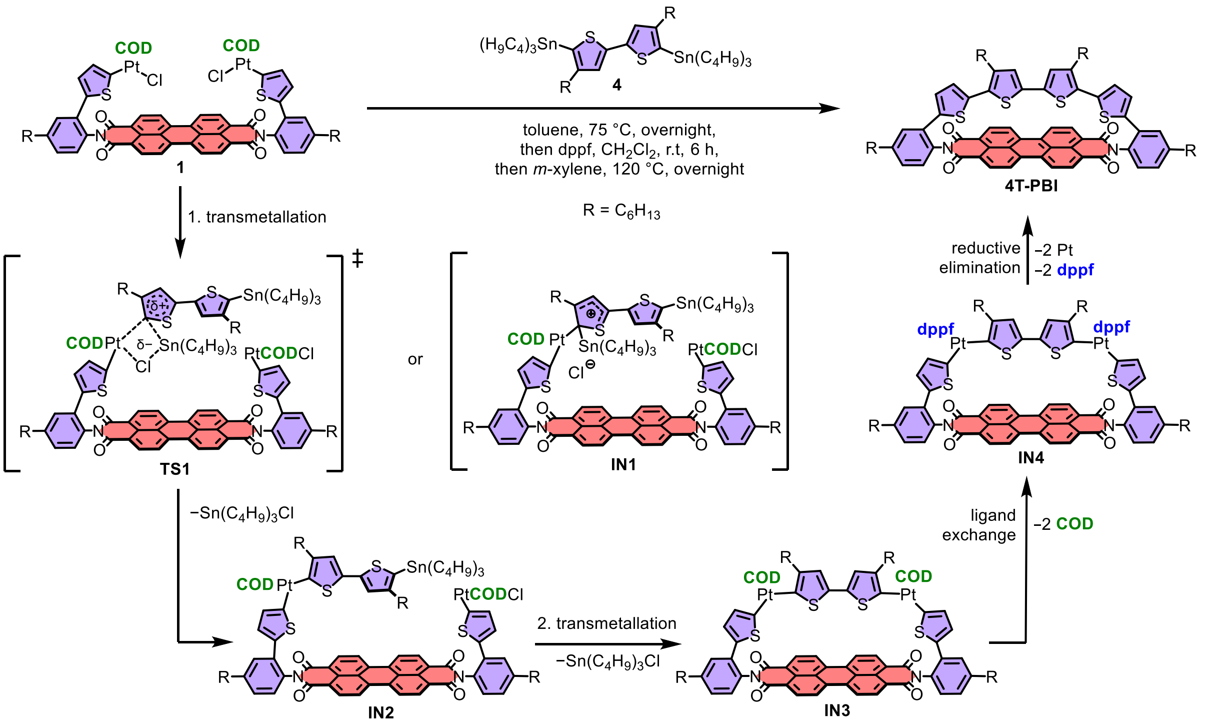

Figure S1. Proposed reaction mechanism for the representative formation of **4T-PBI** starting from Pt-complex **3**.

The addition of stannylated bithiophene **4** to Pt-precursor **3** leads to one *inter*- (**IN2**) and one *intra*molecular (**IN3**) transmetallation. As proposed by Eaborn *et al.*[S12] for similar complex formations of $PtCODCl_2$ with aryl stannanes, this is presumably either achieved *via* a four-centre transition state **TS1** or a Wheland like intermediate **IN1**. This reaction step is conducted under *pseudo* high dilution conditions to favour the desired *intra*molecular Pt-complex formation after the first transmetallation. It is unknown whether both transmetallation steps proceed in a concerted way or stepwise. Concerning the conformation of the oligothiophene chain, most likely a *cis*-orientation is already present at the Pt-complex step (**IN3**) in order to accommodate for the square-planar Pt-complex geometry and sterical demand of the attached hexyl chains R.

Next, a ligand COD-dppf exchange reaction yields **IN4**. The dppf ligand is utilized to

facilitate the subsequent reductive elimination which was demonstrated for *cis*-Pt-(ArAr') complexes.[S13] This last step is conducted at elevated temperatures of 120 °C to initiate the thermally induced reductive elimination.[S14]

Single Crystal X-ray Analysis

Table S1. Crystal data and structure refinement for **4T-PBI** (CCDC2143611).

Empirical formula	$C_{77}H_{74}Br_2N_2O_4S_4$	
Formula weight	1379.44 g/mol	
Temperature	100(2) K	
Wavelength	1.54178 Å	
Crystal system	Triclinic	
Space group	$P\bar{1}$	
Unit cell dimensions	a = 8.0721(3) Å	α = 91.307(2)°
	b = 19.3157(6) Å	β = 97.059(2)°
	c = 21.4211(7) Å	γ = 101.619(2)°
Volume	3242.81(19) Å^3	
Z	2	
Density (calculated)	1.413 g/cm^3	
Absorption coefficient	3.208 mm^{-1}	
F(000)	1432	
Crystal size	0.158 × 0.064 × 0.020 mm	
Theta range for data collection	2.338 to 72.465 °	
Index ranges	$-9 \le h \le 9$, $-23 \le k \le 23$, $-26 \le l \le 26$	
Reflections collected	87488	
Independent reflections	12757 [R_{int} = 0.0531]	
Completeness to theta = 67.679 °	99.9%	
Absorption correction	Semi-empirical from equivalents	
Refinement method	Full-matrix least-squares on F^2	
Data / restraints / parameters	12757 / 296 / 914	
Goodness-of-fit on F^2	1.031	
Final R indices [I > 2sigma(I)]	R_1 = 0.0840, wR_2 = 0.2460	
R indices (all data)	R_1 = 0.0947, wR_2 = 0.2585	
Extinction coefficient	n/a	
Largest diff. peak and hole	1.373 and –1.420 e·Å^{-3}	

Table S2. Crystal data and structure refinement for **7T-PBI** (CCDC2143364).

Empirical formula	$C_{400.63}$ H_{312} $Cl_{3.40}$ N_8 $O_{44.25}$ S_{28}	
Formula weight	6964.24 g/mol	
Temperature	100(2) K	
Wavelength	0.61992 Å	
Crystal system	Monoclinic	
Space group	$P2_1/n$	
Unit cell dimensions	a = 16.717(19) Å	α = 90°
	b = 57.42(5) Å	β = 90.59(4)°
	c = 37.86(4) Å	γ = 90°
Volume	36340(65) Å^3	
Z	4	
Density (calculated)	1.273 g/cm^3	
Absorption coefficient	0.179 mm^{-1}	
F(000)	14526.1	
Crystal size	0.100 × 0.100 × 0.050 mm^3	
Theta range for data collection	0.562 to 16.367°.	
Index ranges	−15≤h≤15, −52≤k≤52, −34≤l≤34	
Reflections collected	200878	
Independent reflections	28320 [R_{int} = 0.1509]	
Completeness to theta = 16.367°	99.0%	
Absorption correction	None	
Refinement method	Full-atrix least-squares on F^2	
Data / restraints / parameters	28320 / 10992 / 5088	
Goodness-of-fit on F^2	1.327	
Final R indices [I > 2sigma(I)]	R_1 = 0.1568, wR_2 = 0.3885	
R indices (all data)	R_1 = 0.2946, wR_2 = 0.4729	
Extinction coefficient	n/a	
Largest diff. peak and hole	0.563 and −0.271 e·Å^{-3}	

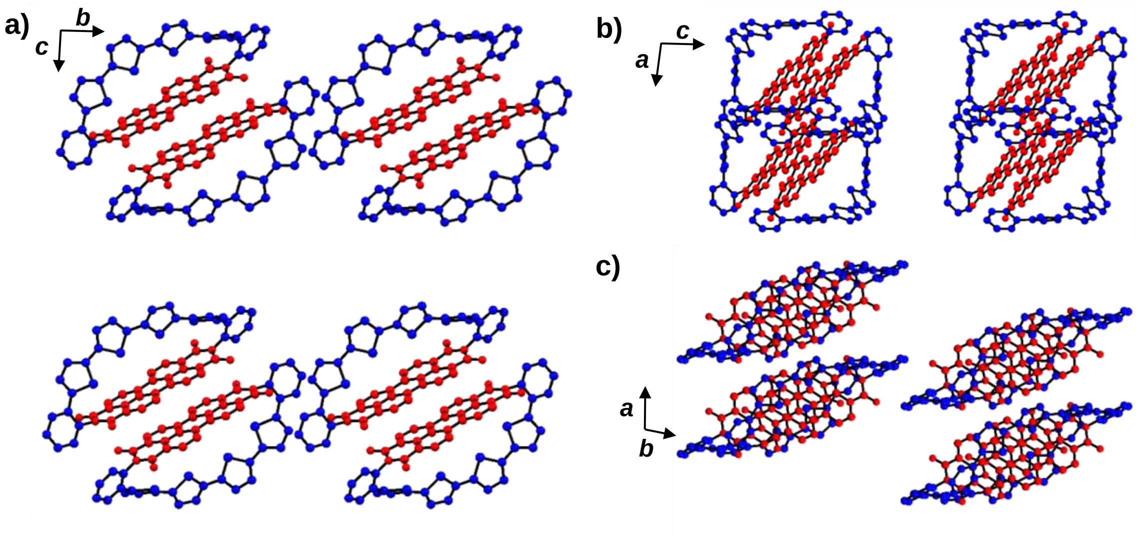

Figure S2. Crystal packing of **4T-PBI** seen along the a) *a*-, b) *b*-, and c) *c*-axes, respectively. PBI chromophores are coloured in red and the oligothiophene bridges in blue. Disordered aliphatic chains as well as solvent molecules were omitted for clarity.

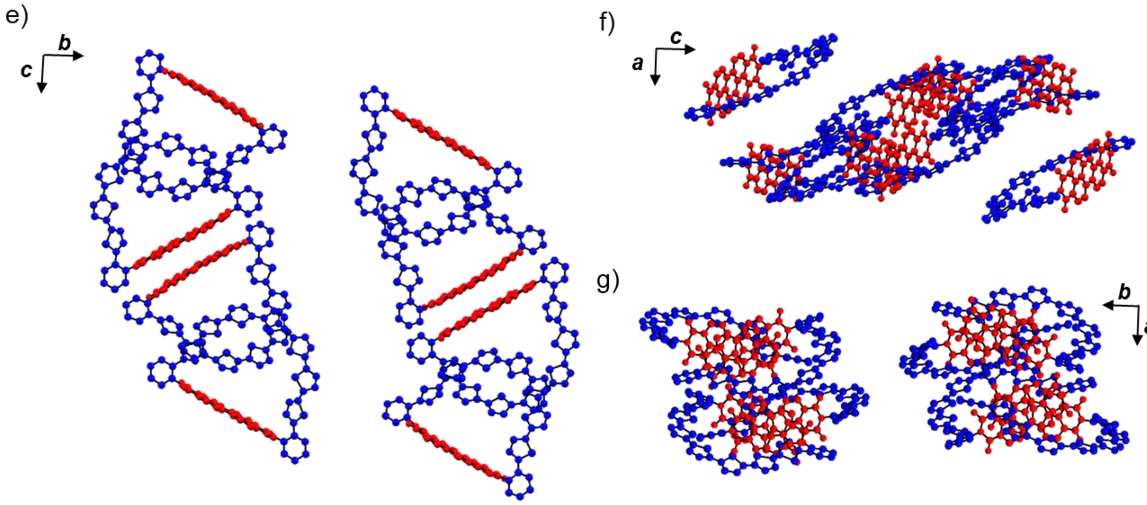

Figure S3. Crystal packing of **7T-PBI** seen along the a) *a*-, b) *b*-, and c) *c*-axes, respectively. PBI chromophores are coloured in red, the oligothiophene bridges in blue. Disordered aliphatic chains as well as solvent molecules were omitted for clarity.

^{1}H NMR Spectra Comparison

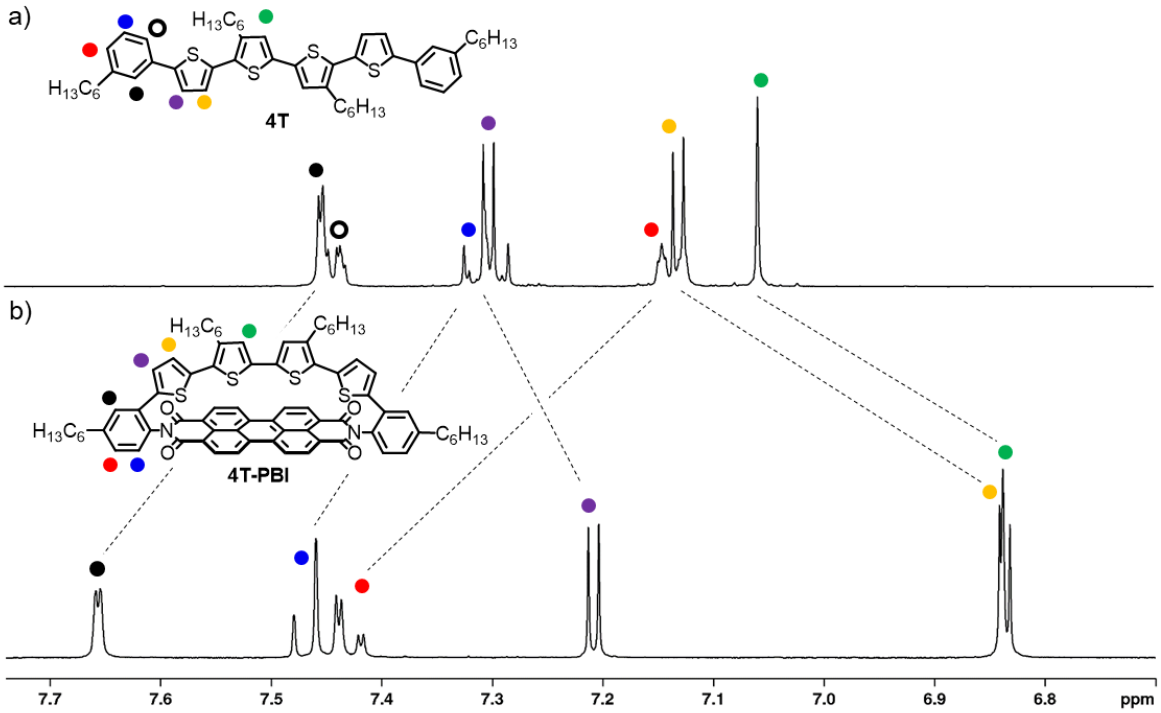

Figure S4. Excerpt of the ^{1}H NMR spectra of a) oligothiophene moiety **4T**[S5] and b) **4T-PBI** in CD_2Cl_2 at room temperature.

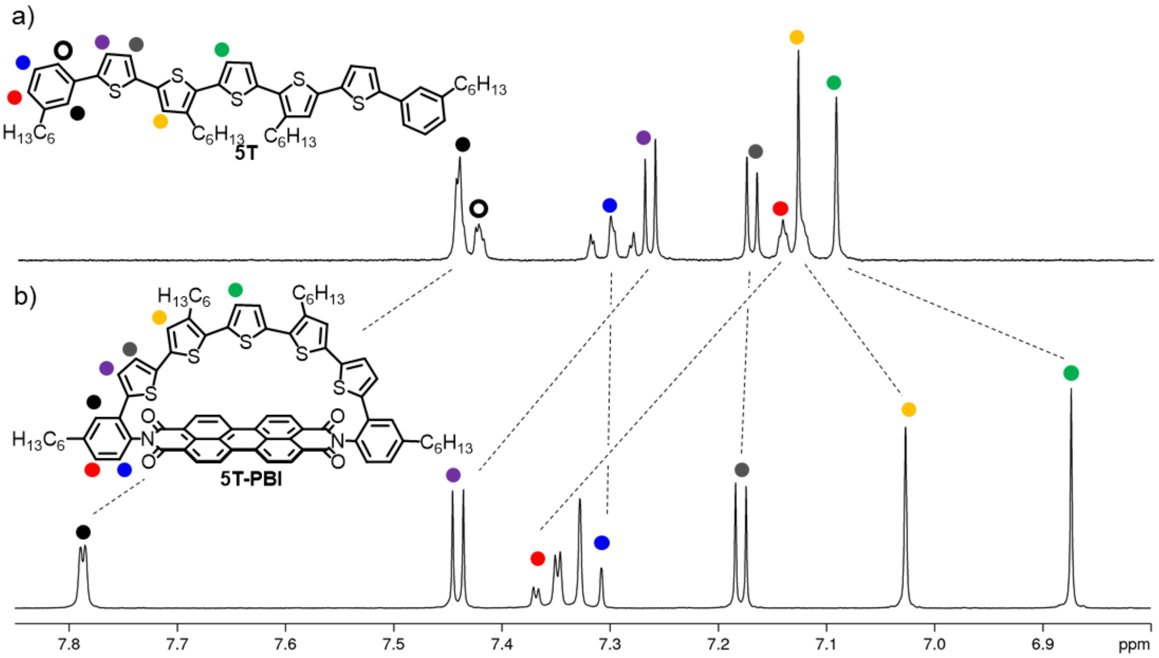

Figure S5. Excerpt of the ^{1}H NMR spectra of a) oligothiophene moiety **5T**[S5] and b) **5T-PBI**[S4] in CD_2Cl_2 at room temperature.

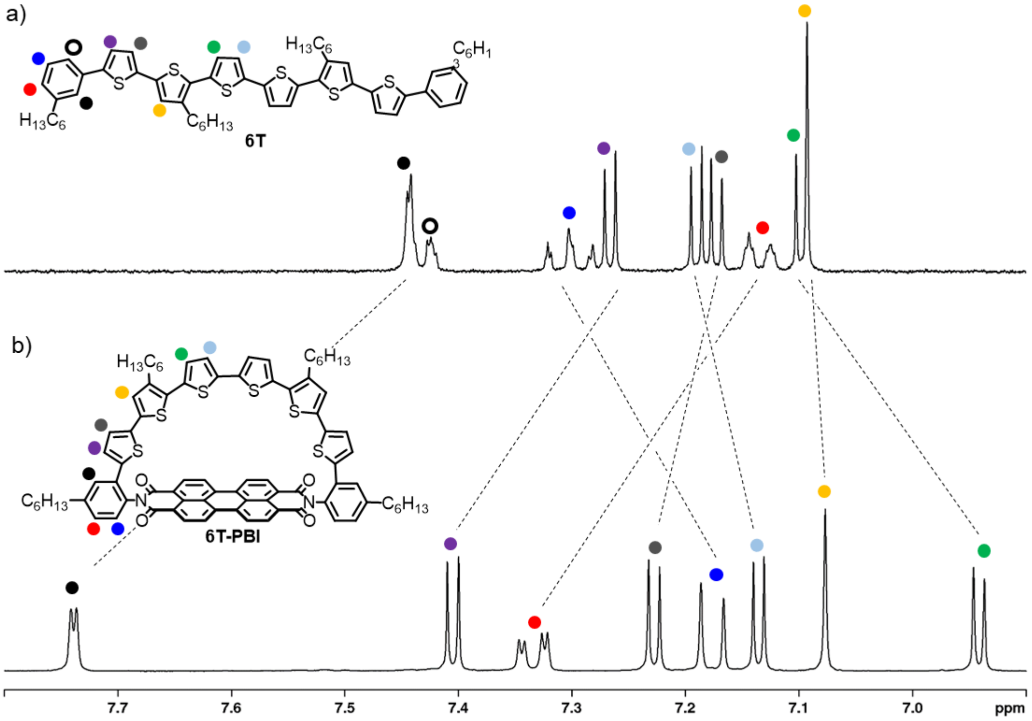

Figure S6. Excerpt of the ^{1}H NMR spectra of a) oligothiophene moiety **6T**[S5] and b) **6T-PBI** in CD_2Cl_2 at room temperature.

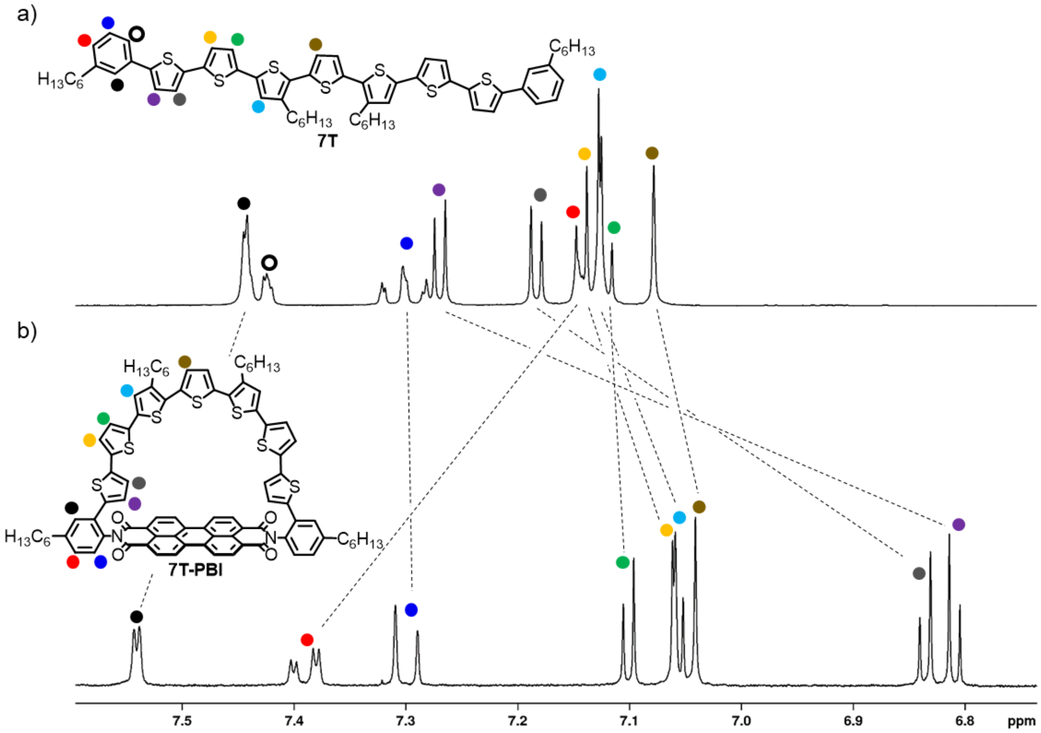

Figure S7. Excerpt of the ^{1}H NMR spectra of a)) oligothiophene moiety **7T**[S5] and b) **7T-PBI** in CD_2Cl_2 at room temperature.

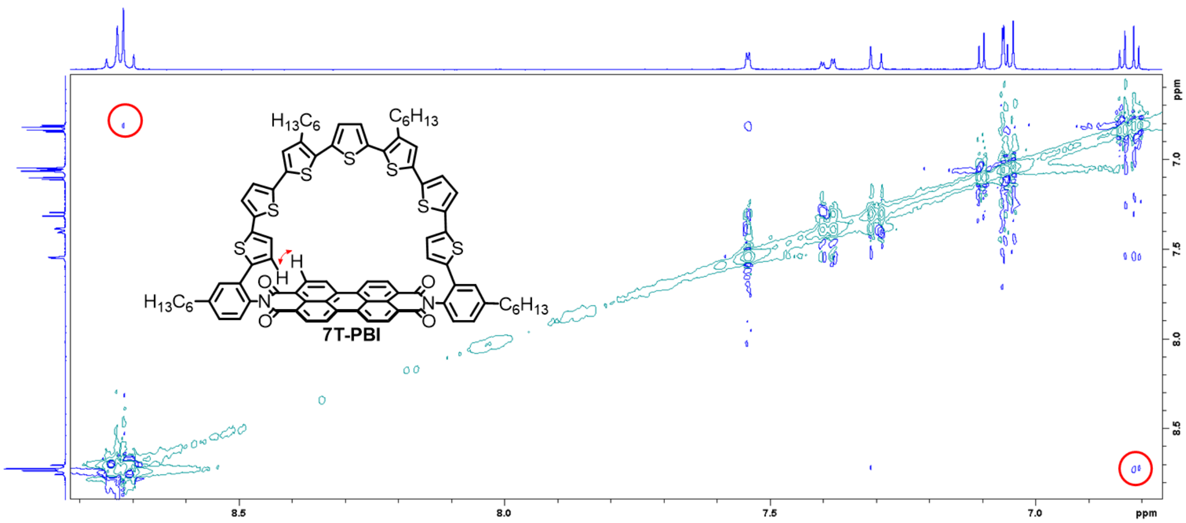

Figure S8. Excerpt of the ROESY spectrum of **7T-PBI** in CD_2Cl_2 at room temperature. The thiophene and PBI proton cross signals are highlighted by red circles.

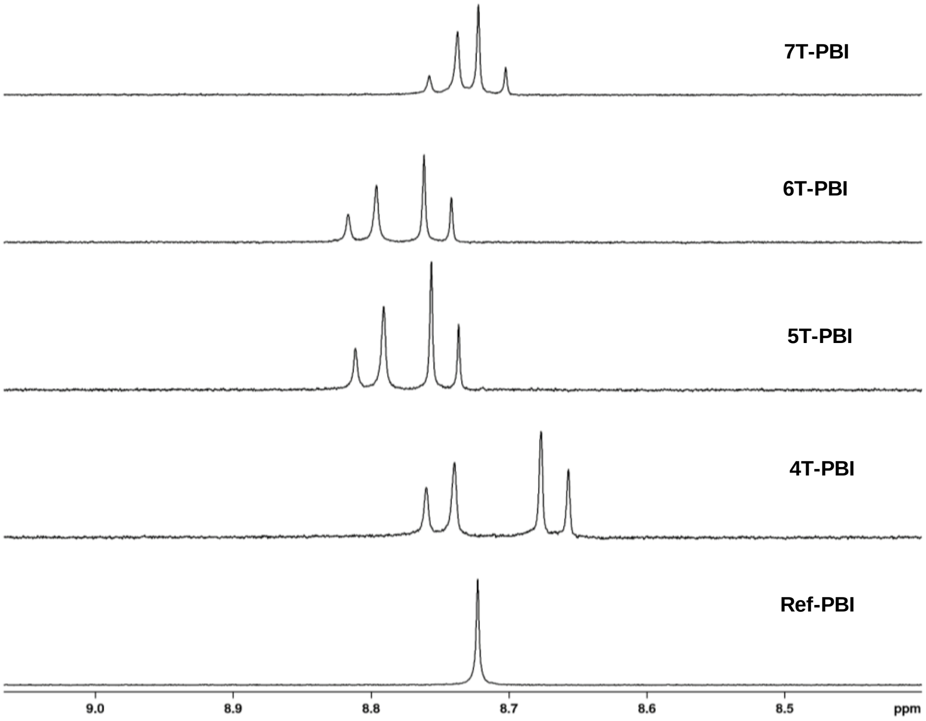

Figure S9. PBI proton signals of **Ref-PBI** in comparison to those of the macrocycles **4T-PBI**, **5T-PBI**[S4], **6T-PBI** and **7T-PBI** (from bottom to top). ^{1}H NMR spectra were recorded in CD_2Cl_2 at room temperature at equal concentrations of $c_0 = 10^{-4}$ M.

Spectroelectrochemistry

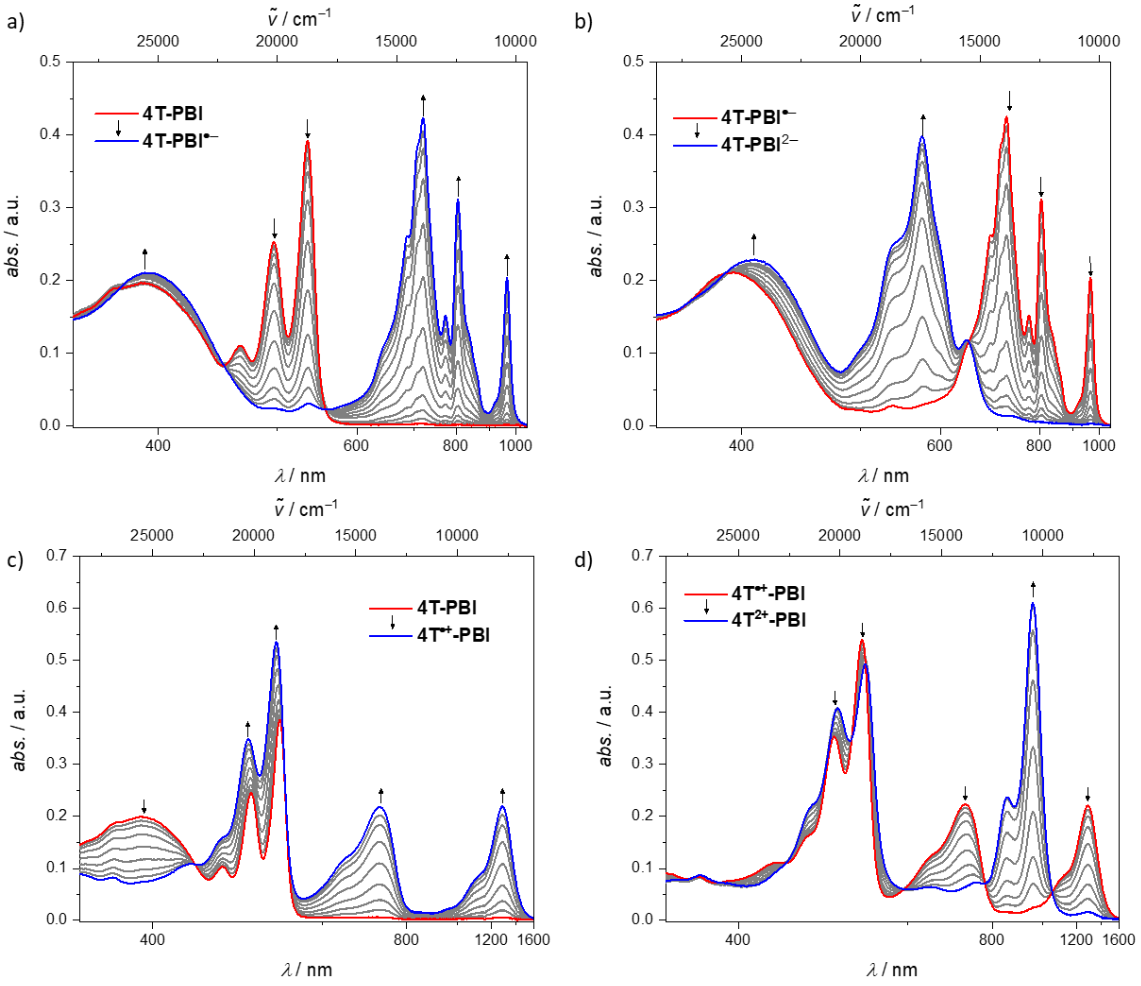

Figure S10. UV/Vis/NIR absorption changes of **4T-PBI** upon electrochemical reduction to a) **4T-PBI•−** and b) **4T-PBI2−** and electrochemical oxidation to c) **4T•+-PBI** and d) **4T2+-PBI** in CH_2Cl_2 solutions of Bu_4NPF_6 at r.t. The arrows in the graphs indicate the spectral changes upon decreasing (a,b) and increasing (c,d) electric potential. Spectra at initial (red) and final (blue) potential are always highlighted in a)-d).

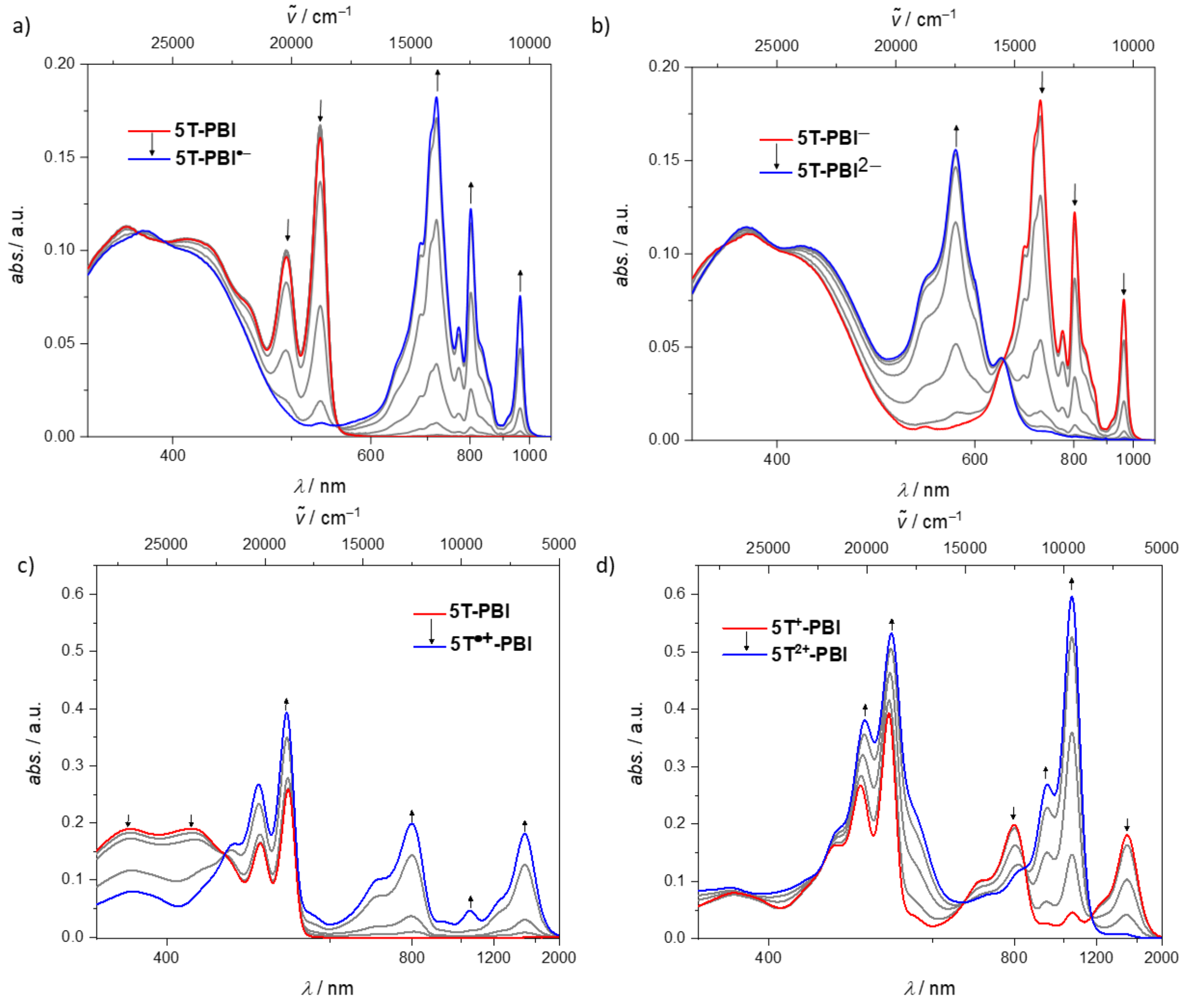

Figure S11. UV/Vis/NIR absorption changes of **5T-PBI**[S4] upon electrochemical reduction to a) **5T-PBI**$^{\bullet-}$ and b) **5T-PBI**$^{2-}$ and electrochemical oxidation to c) **5T**$^{\bullet+}$**-PBI** and d) **5T**$^{2+}$**-PBI** in CH_2Cl_2 solutions of Bu_4NPF_6 at r.t. The arrows in the graphs indicate the spectral changes upon decreasing (a,b) and increasing (c,d) electric potential. Spectra at initial (red) and final (blue) potential are always highlighted in a)-d).

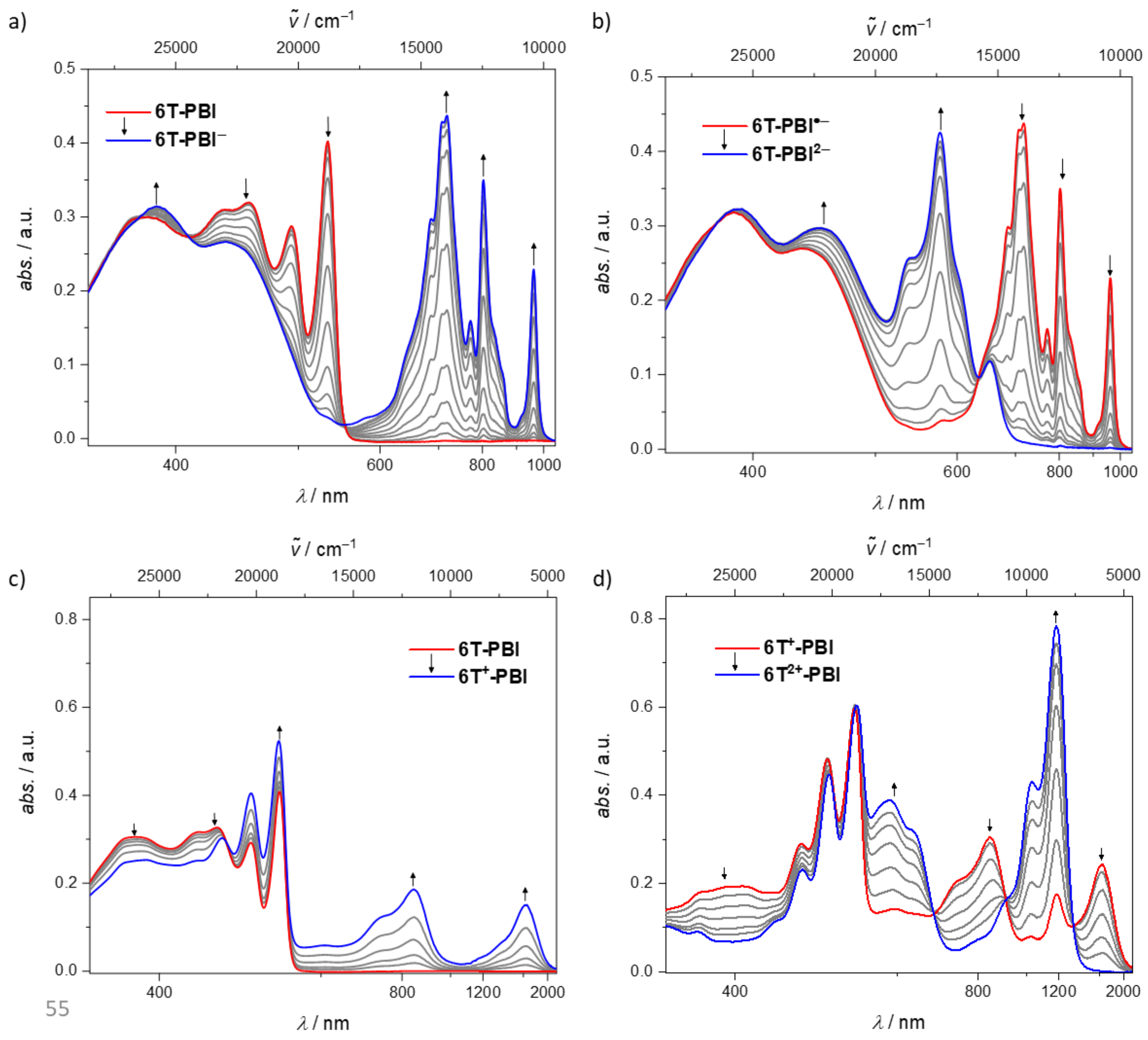

Figure S12. UV/Vis/NIR absorption changes of **6T-PBI** upon electrochemical reduction to a) **6T-PBI**$^{\bullet-}$ and b) **6T-PBI**$^{2-}$ and electrochemical oxidation to c) **6T**$^{\bullet+}$**-PBI** and d) **6T**$^{2+}$**-PBI** in CH_2Cl_2 solutions of Bu_4NPF_6 at r.t. The arrows in the graphs indicate the spectral changes upon decreasing (a,b) and increasing (c,d) electric potential. Spectra at initial (red) and final (blue) potential are always highlighted in a)-d).

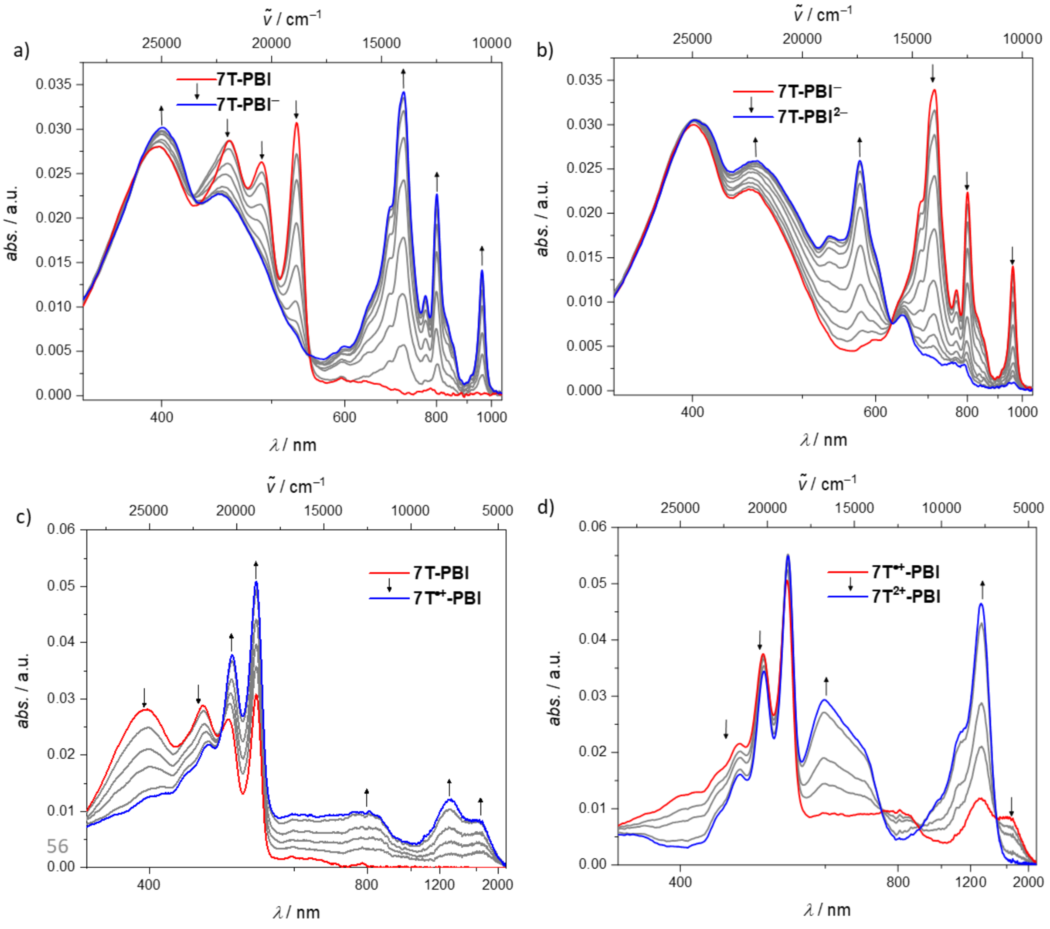

Figure S13. UV/Vis/NIR absorption changes of **7T-PBI** upon electrochemical reduction to a) **7T-PBI$^{\bullet-}$** and b) **7T-PBI^{2-}** and electrochemical oxidation to c) **7T$^{\bullet+}$-PBI** and d) **7T^{2+}-PBI** in CH_2Cl_2 solutions of Bu_4NPF_6 at r.t. The arrows in the graphs indicate the spectral changes upon decreasing (a,b) and increasing (c,d) electric potential. Spectra at initial (red) and final (blue) potential are always highlighted in a)-d).

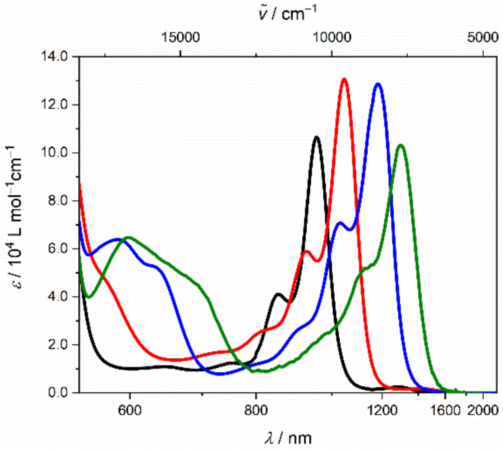

Figure S14. UV/Vis/NIR absorption spectra of the dications of **4T-PBI** (black), **5T-PBI**[S4] (red), **6T-PBI** (blue) and **7T-PBI** (green), respectively. All measurements were carried out in CH_2Cl_2 solutions with Bu_4NPF_6 at room temperature ($c_0 = 10^{-4}$ M).

Optical Properties in Cyclohexane

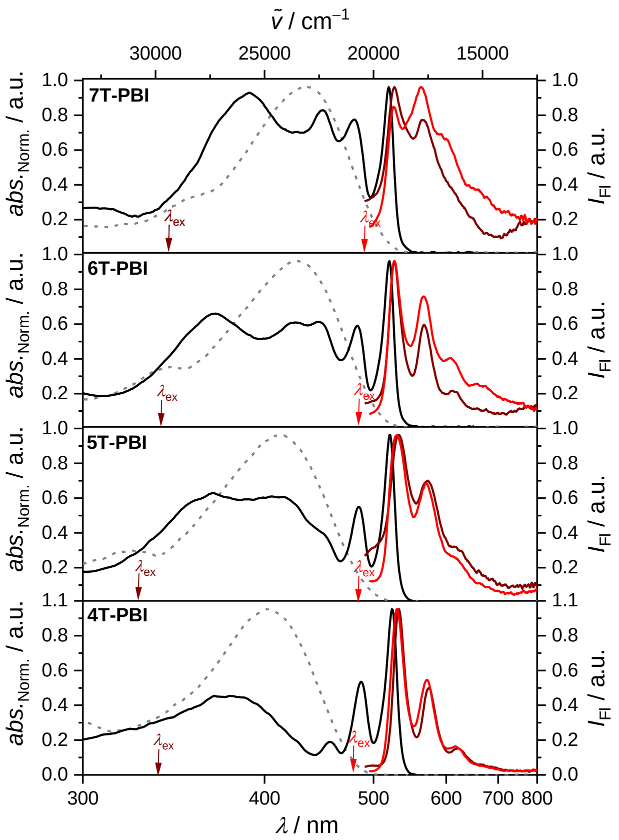

Figure S15. Normalized UV/Vis absorption (black solid, grey dashed) and emission (red, maroon) spectra of **4T-PBI**, **5T-PBI**[S4], **6T-PBI** and **7T-PBI** (black, red, maroon) as well as of the corresponding reference donor structures **4T**-**7T**[S5] (grey) in cyclohexane at room temperature (c_0 = 10^{-7} M). The wavelengths for excitation (λ_{ex}) at 400 nm (maroon) and 480 nm (red) to obtain the fluorescence spectra are highlighted by arrows.

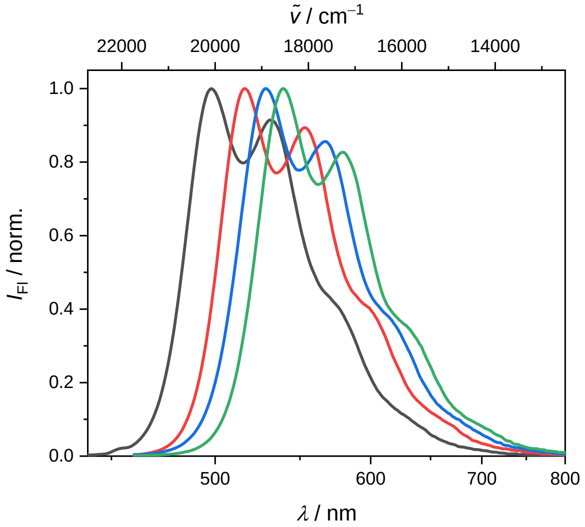

Figure S16. Normalized emission spectra of **4T** (black line), **5T** (red line), **6T** (blue line), and **7T** (green line) in cyclohexane (c_0=10^{-7} M) at room temperature.[S5]

DFT and TDDFT calculations

The calculations of the macrocyclic strain energies (ΔE_{strain}) have been performed as described in ref. [S4].

Table S3. Macrocyclic strain energies of all macrocycles.

	**4T-PBI**	**5T-PBI**	**6T-PBI**	**7T-PBI**	**7T-PBI-T**
ΔE_{strain} / kJ·mol^{-1}	19.6	13.9	17.2	22.5	30.6

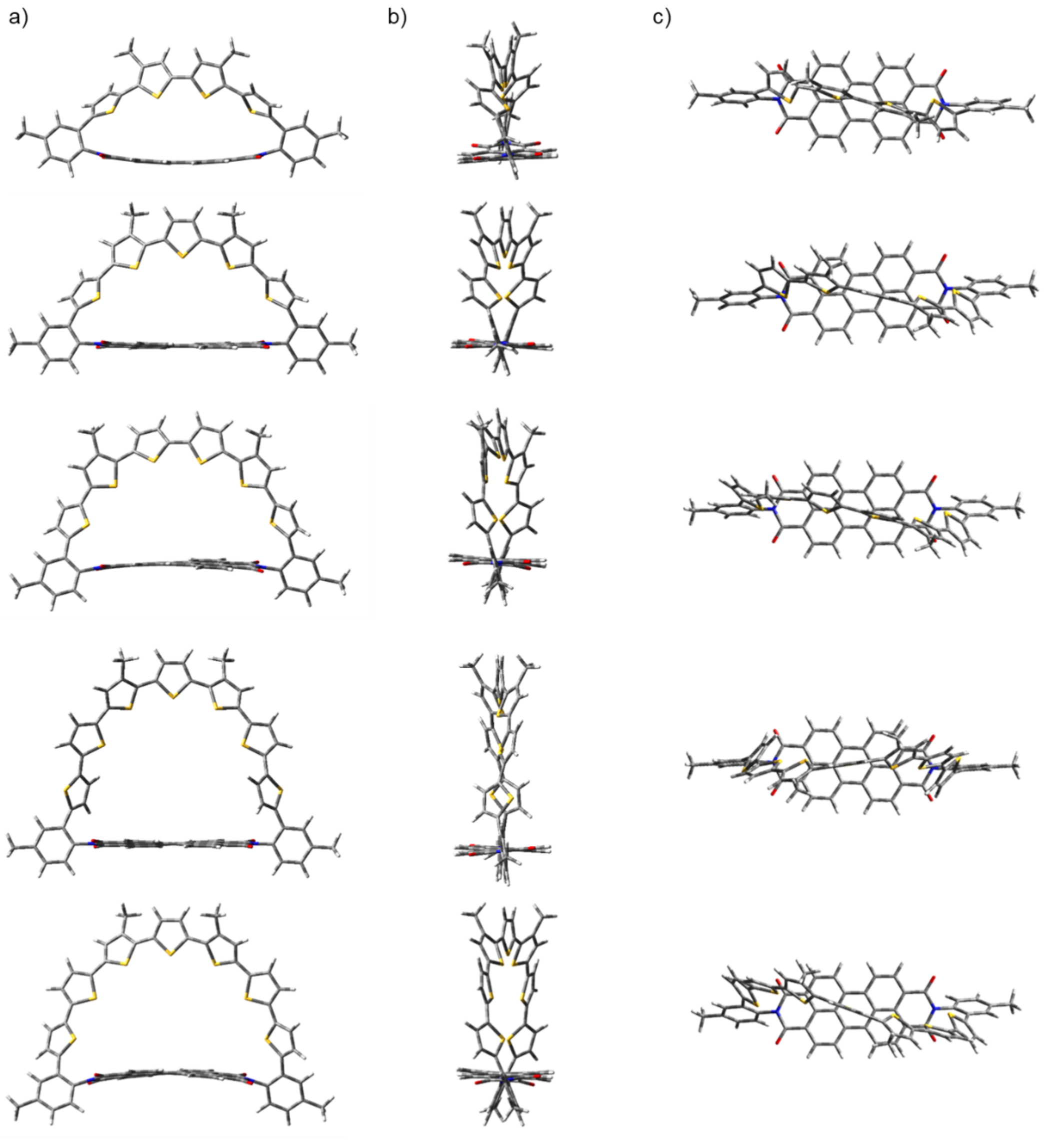

Figure S17. Side view (a), view along the *N,N´*-axis (b) and top (c) view onto the π-surface of **4T-PBI**, **5T-PBI**[S4], **6T-PB**, **7T-PBI** and **7T-PBI-T** (from top to bottom). The quantum mechanics calculations were carried out on the level of B3LYP density functional with the 6-31G(d) basis set as implemented in with Gaussian 16. Aliphatic chains were replaced by methyl groups. Color code: carbon = light grey, hydrogen = white, nitrogen = blue, oxygen = red, sulfur = yellow.

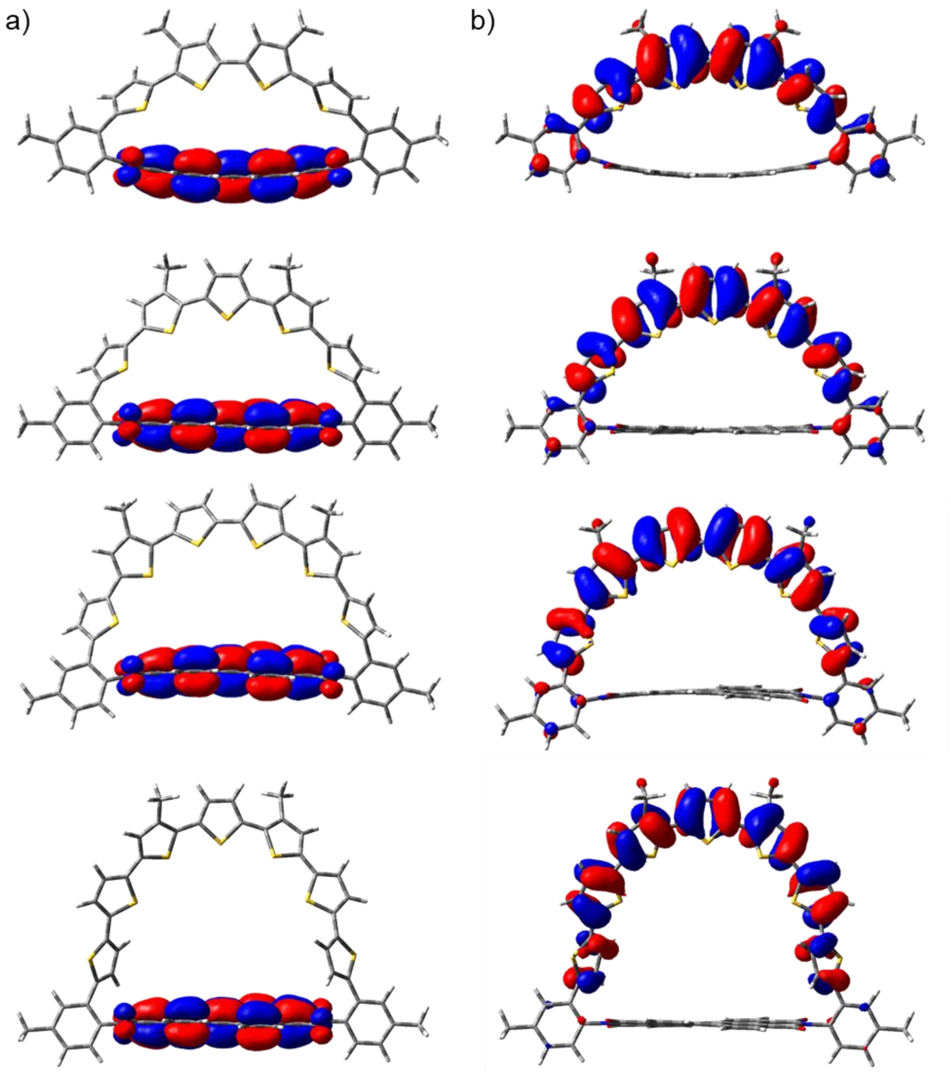

Figure S18. LUMOs (a) and HOMOs (b) of **4T-PBI**, **5T-PBI**[S4], **6T-PB** and **7T-PBI** (from top to bottom) based on geometry optimized structures from DFT calculations. The quantum mechanics calculations were carried out on the level of B3LYP density functional with the 6-31G(d) basis set as implemented in with Gaussian 16. Aliphatic chains were replaced by methyl groups.

Table S4. First excited state (S_1) energy predictions of **4T-PBI**, **5T-PBI**[S4], **6T-PB** and **7T-PBI** with TDDFT at the B3LYP/6-31G(d) level of theory (H = HOMO, L = LUMO).

Compound	Excitation Energy / eV	Wavelength / nm	Osc. Strength	Contribution
**4T-PBI**	1.36	911	0.0000	H → L(100%)
**5T-PBI**	1.18	1051	0.0000	H → L(100%)
**6T-PBI**	1.17	1064	0.0000	H → L(100%)
**7T-PBI**	1.14	1090	0.0002	H → L(100%)

Transient Absorption Spectra

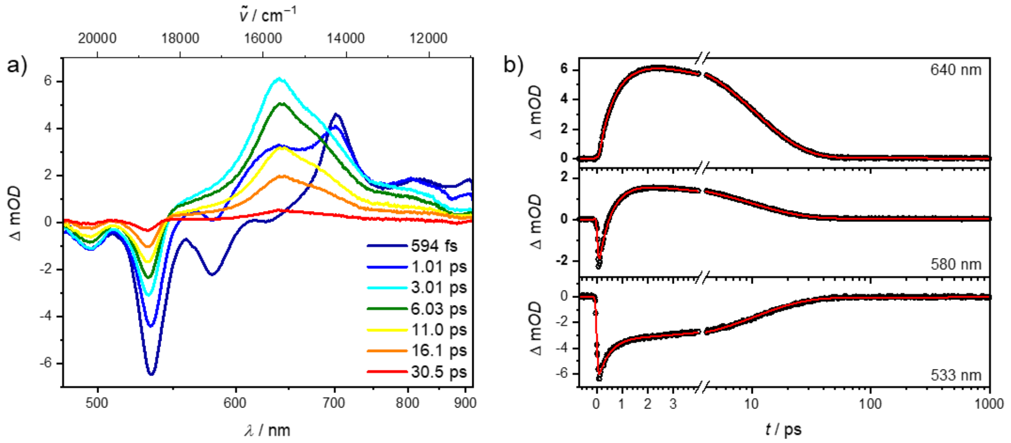

Figure S19. a) Transient absorption spectra of **4T-PBI** in CH_2Cl_2 after excitation at 530 nm and b) time scans and fit (red line) at selected wavelengths.

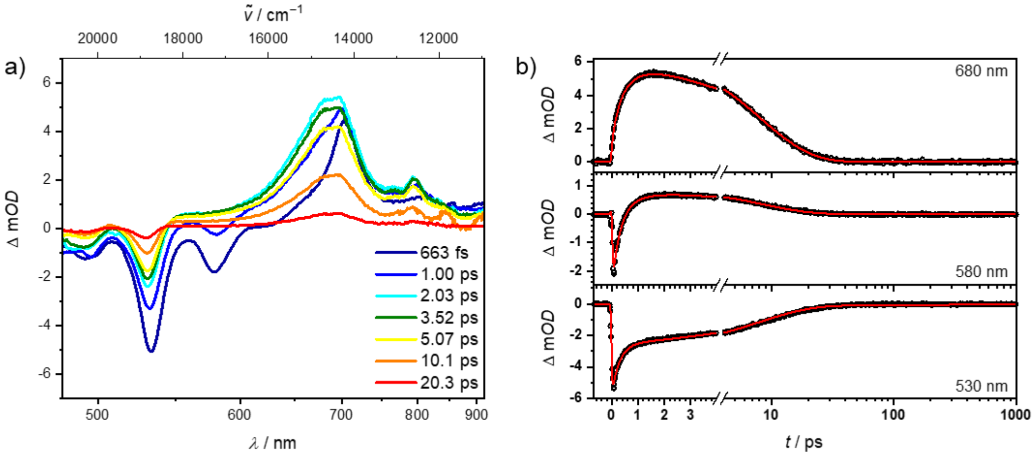

Figure S20. a) Transient absorption spectra of **6T-PBI** in CH_2Cl_2 after excitation at 530 nm and b) time scans and fit (red line) at selected wavelengths.

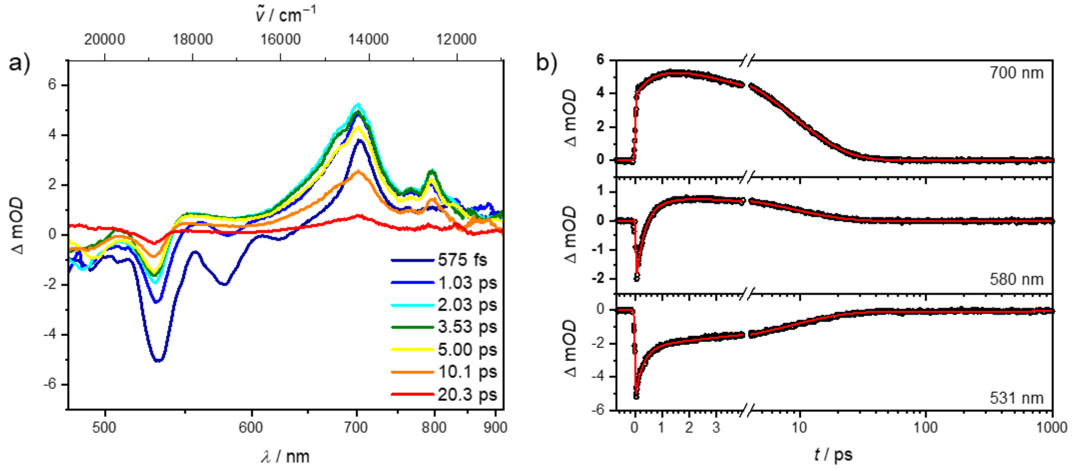

Figure S21. a) Transient absorption spectra of **7T-PBI** in CH_2Cl_2 after excitation at 530 nm and b) time scans and fit (red line) at selected wavelengths.

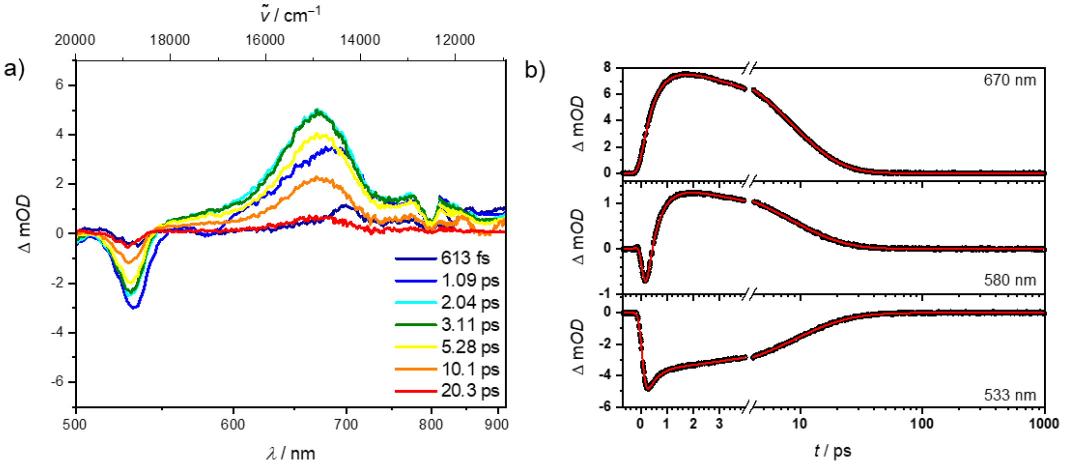

Figure S22. a) UV/Vis region of the transient absorption spectra of **5T-PBI** in CH_2Cl_2 after excitation at 400 nm and b) time scans and fit (red line) at selected wavelengths.

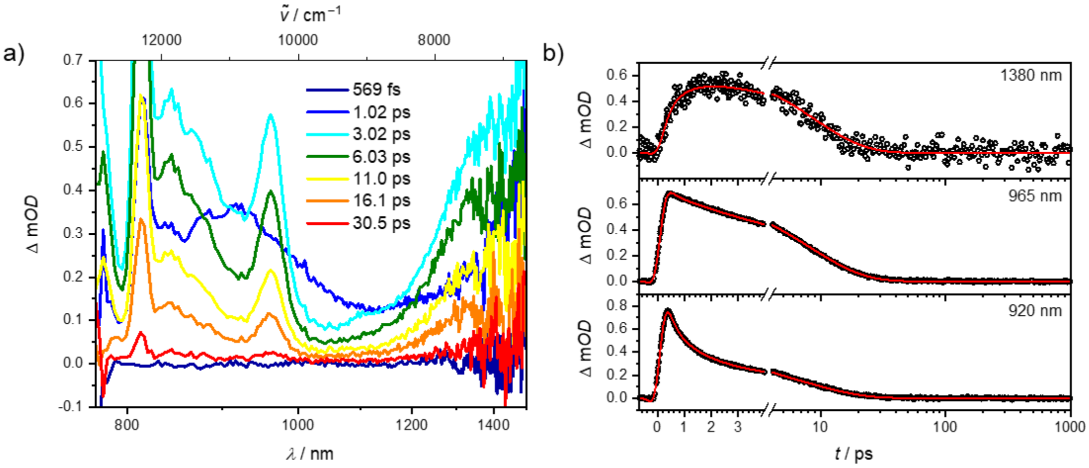

Figure S23. a) NIR region of the transient absorption spectra of **5T-PBI** in CH_2Cl_2 after excitation at 400 nm and b) time scans and fit (red line) at selected wavelengths.

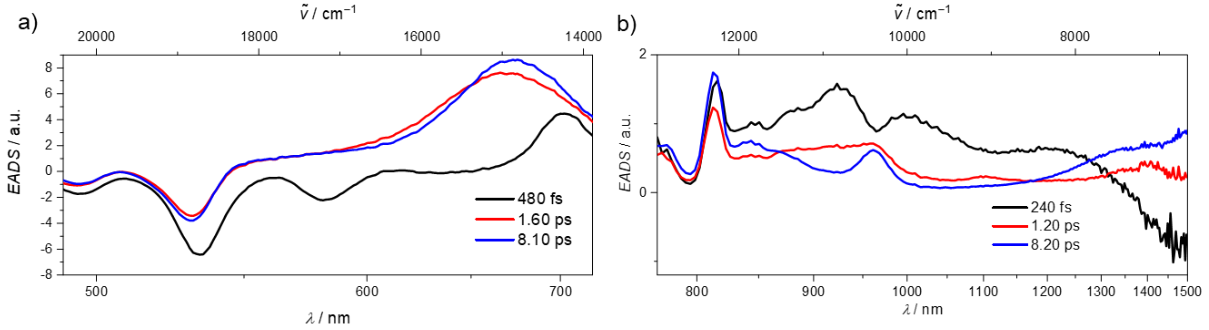

Figure S24. Evolution associated difference spectra (EADS) and lifetimes in the a) UV/Vis and b) NIR region from a global fit analysis of the transient spectra of **5T-PBI** obtained by excitation at 400 nm in CH_2Cl_2 ($c_0 = 10^{-4}$ M) at room temperature.

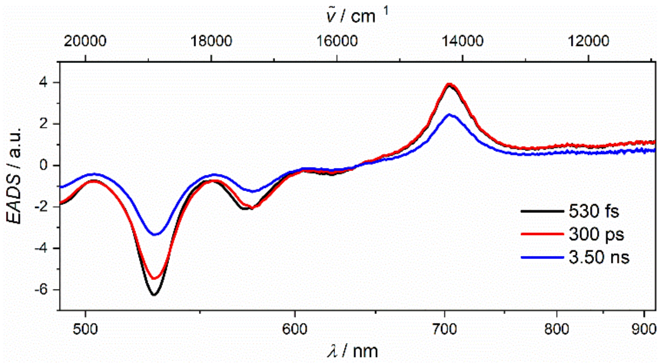

Figure S25. Evolution associated difference spectra (EADS) and lifetimes from a global fit analysis of the transient spectra of a 1:1 mixture comprising **4T** and **Ref-PBI** obtained by excitation at 530 nm in CH_2Cl_2 ($c_0 = 10^{-4}$ M) at room temperature.

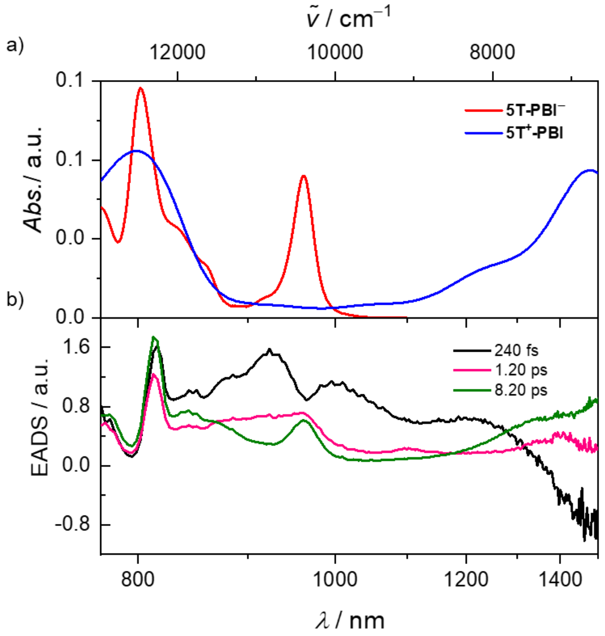

Figure S26. a) Normalized NIR absorption spectra of **5T-PBI** upon electrochemical reduction to **5T-PBI⁻** (red line) and electrochemical oxidation to **5T⁺-PBI** (blue line) in CH_2Cl_2 solutions with Bu_4NPF_6 at room temperature ($c_0 = 10^{-4}$ M).[S4] b) Evolution associated difference spectra (EADS) and lifetimes from a global fit analysis of the transient spectra of **5T-PBI** obtained by excitation at 400 nm in CH_2Cl_2 ($c_0 = 10^{-4}$ M) at room temperature.

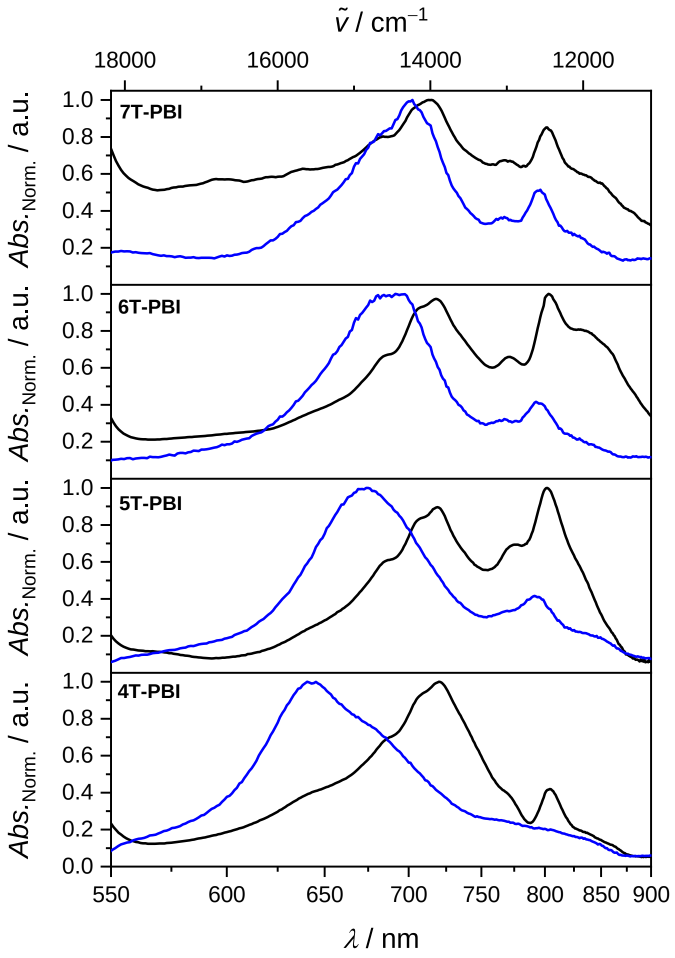

Figure S27. Sum of the normalized anion and cation spectra (black lines) obtained from SEC and the EADS of the normalized "cold" CT/CS state (blue lines) of **4T-PBI**, **5T-PBI**[S4], **6T-PB** and **7T-PBI** in CH_2Cl_2 at room temperature.

NMR Spectra

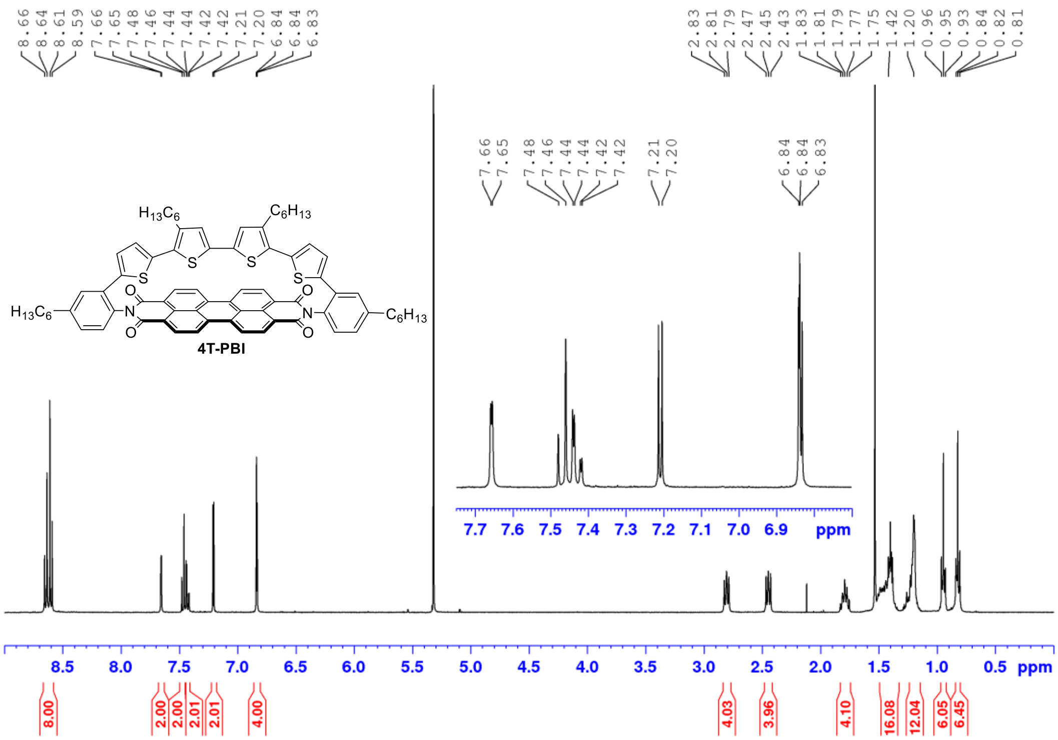

Figure S28. ^{1}H NMR spectrum (400 MHz) of **4T-PBI** in CD_2Cl_2 at 298 K.

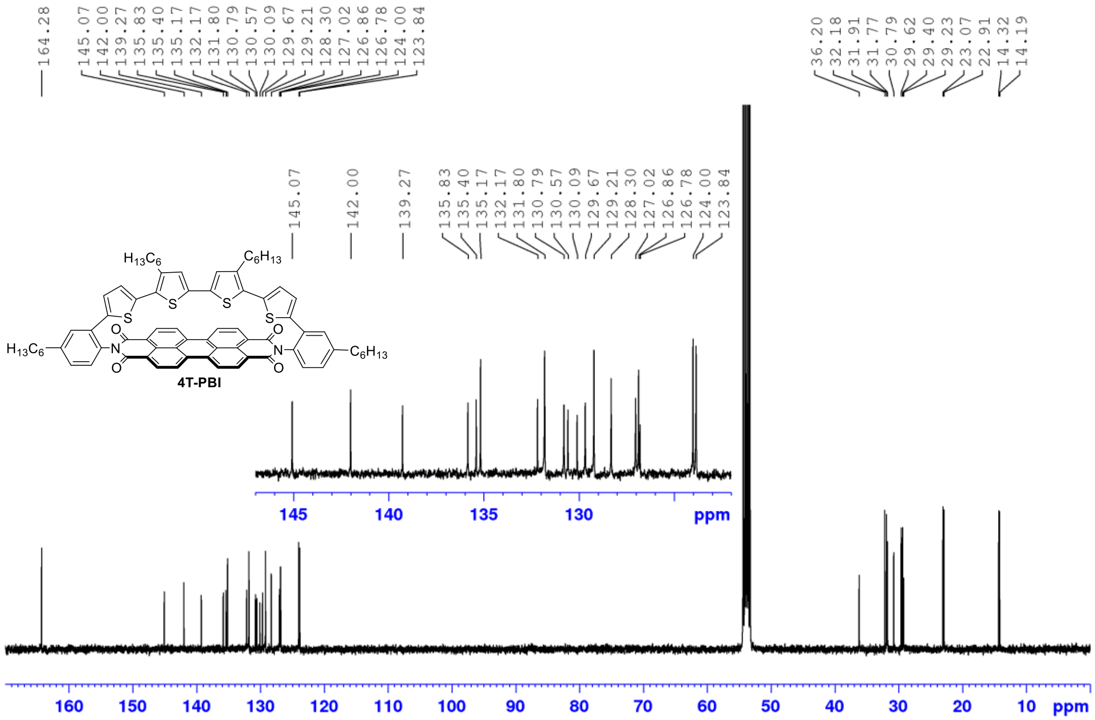

Figure S29. ^{13}C NMR spectrum (101 MHz) of **4T-PBI** in CD_2Cl_2 at 298 K.

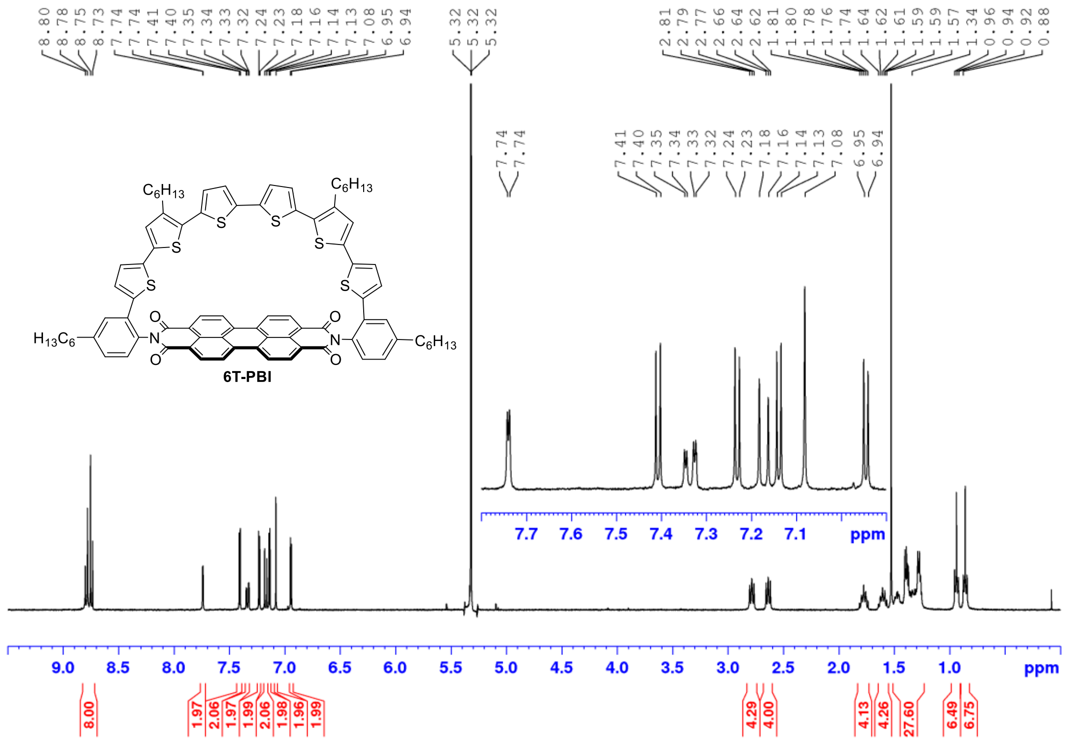

Figure S30. ^{1}H NMR spectrum (400 MHz) of **6T-PBI** in CD_2Cl_2 at 298 K.

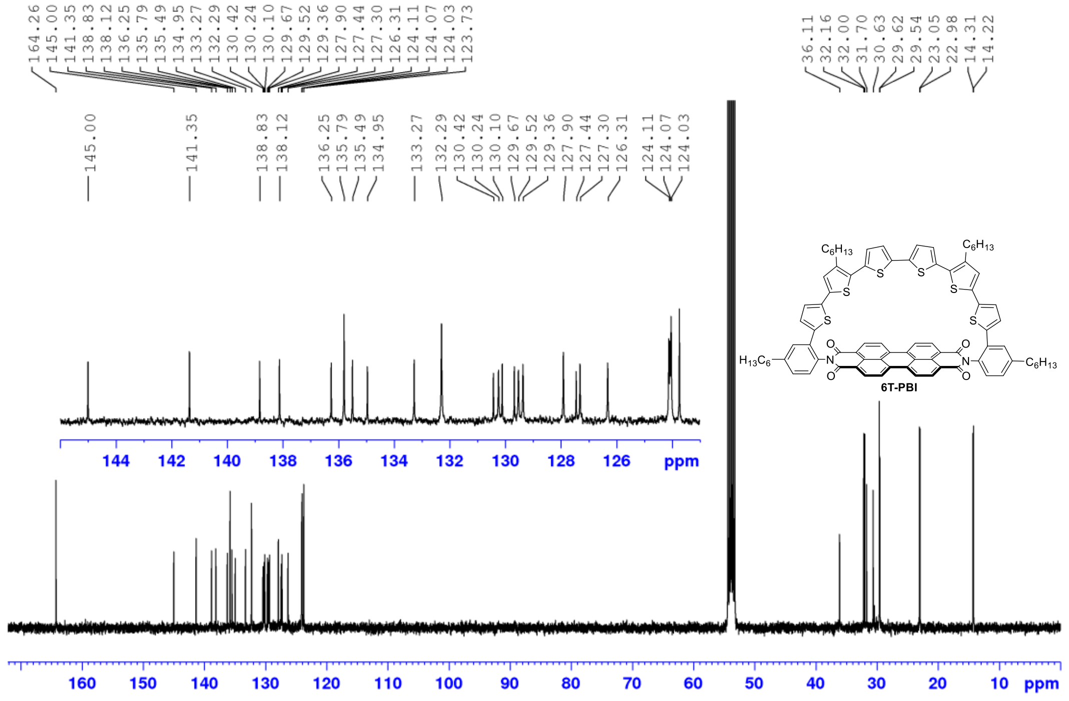

Figure S31. ^{13}C NMR spectrum (101 MHz) of **6T-PBI** in CD_2Cl_2 at 298 K.

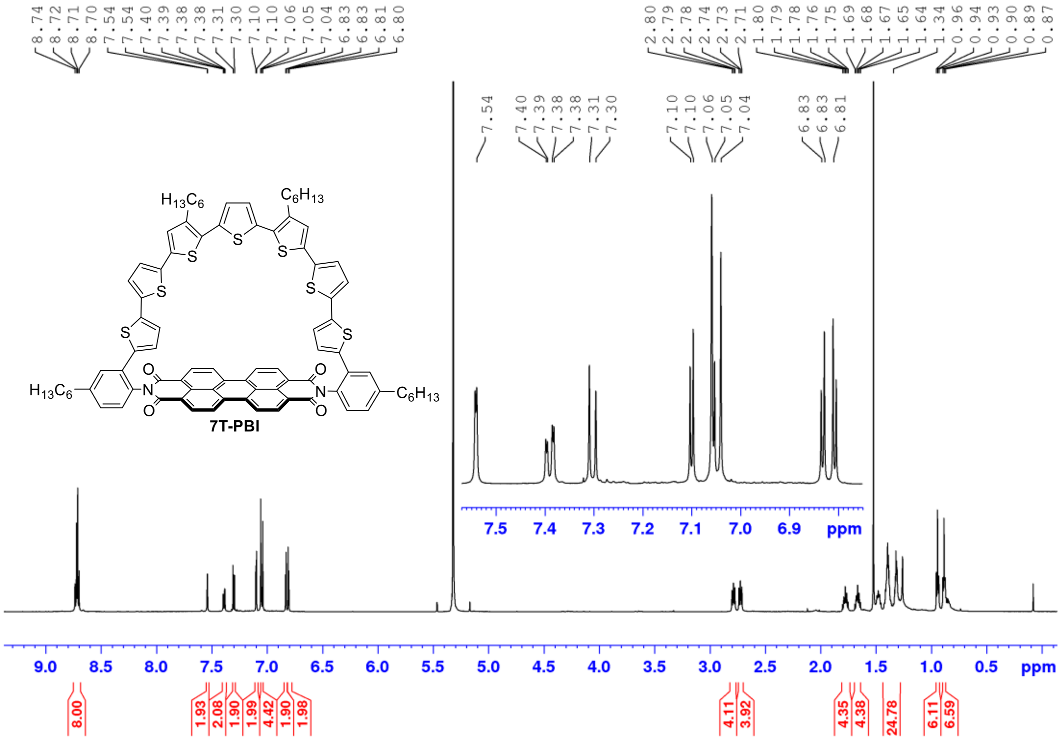

Figure S32. ^{1}H NMR spectrum (600 MHz) of **7T-PBI** in CD_2Cl_2 at 298 K.

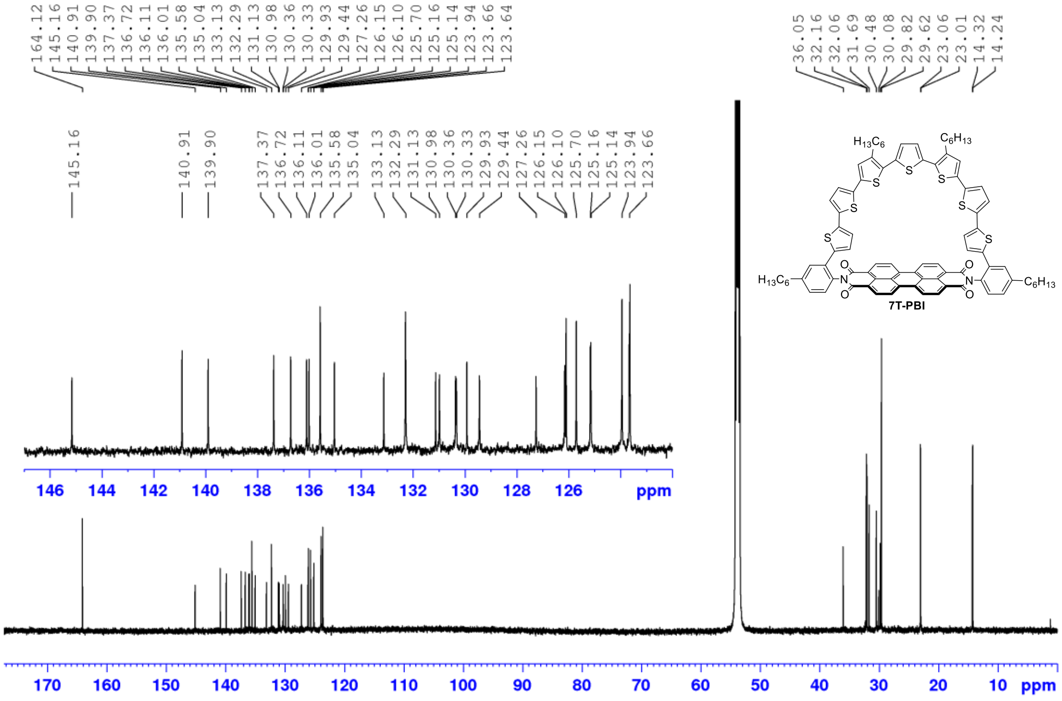

Figure S33. ^{13}C NMR spectrum (150 MHz) of **7T-PBI** in CD_2Cl_2 at 298 K.

Mass Spectra

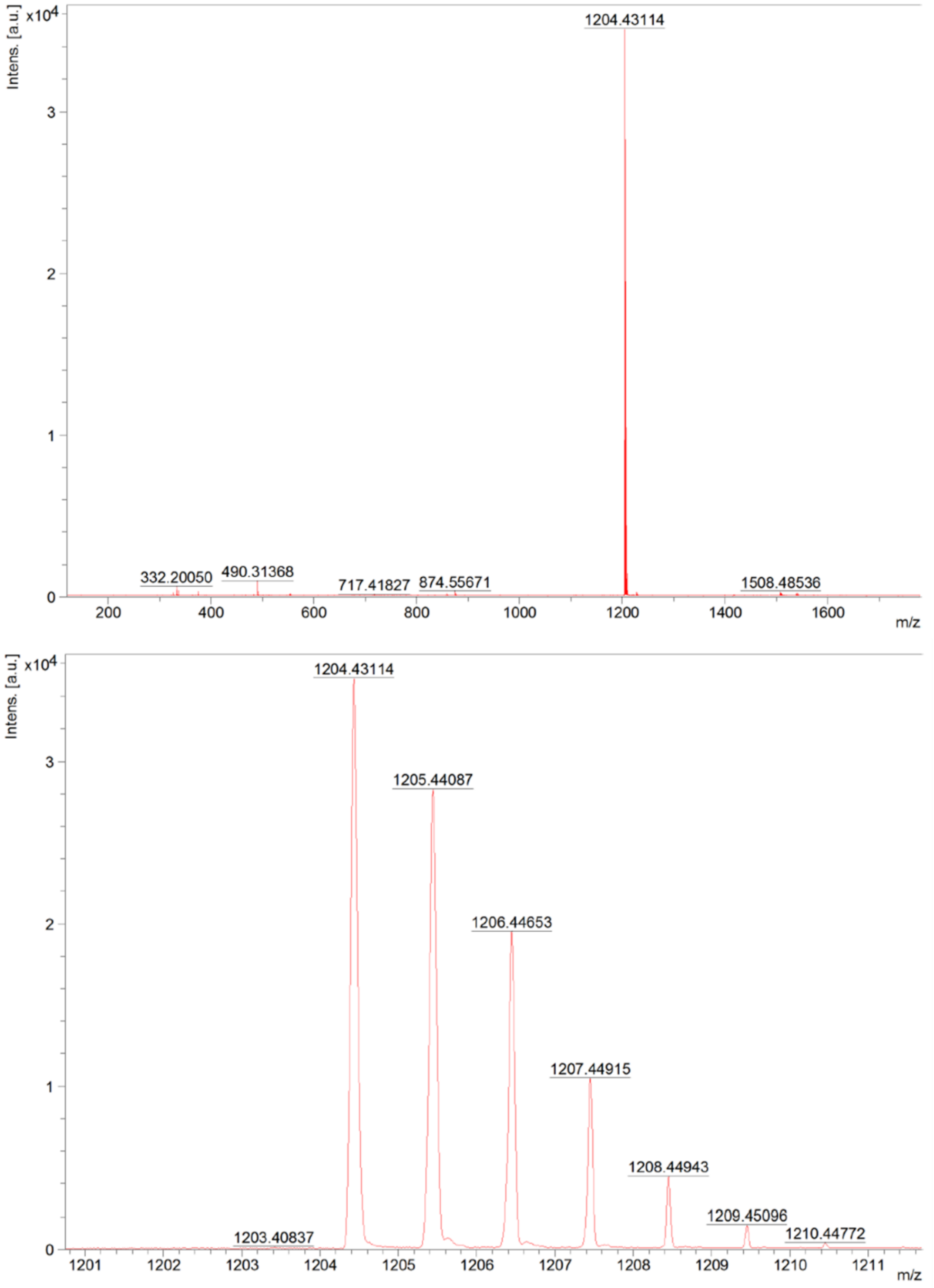

Formula	Mass	Error	mSigma	DblEq	N rule	Electron Configuration
C 76 H 72 N 2 O 4 S 4	1,204.4369	4.8169	28.4696	42.00	ok	odd

Figure S34. HRMS (MALDI-TOF, pos. mode, DCTB in $CHCl_3$) spectra of **4T-PBI**.

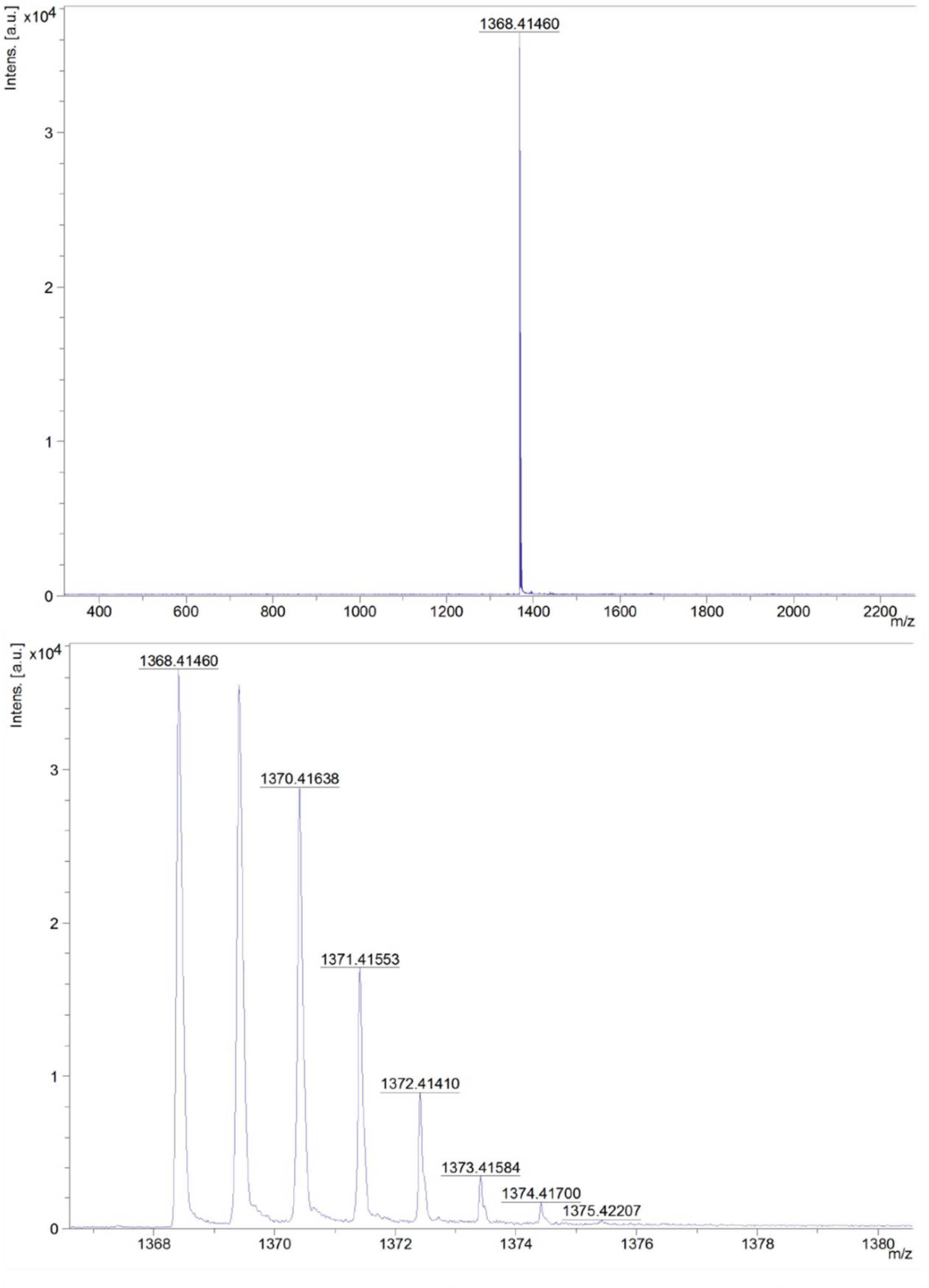

Formula	Mass	Error	mSigma	DblEq	N rule	Electron Configuration
C 84 H 76 N 2 O 4 S 6	1,368.4124	1.6181	30.5827	48.00	ok	odd

Figure S35. HRMS (MALDI-TOF, pos. mode, DCTB in $CHCl_3$) spectra of **6T-PBI**.

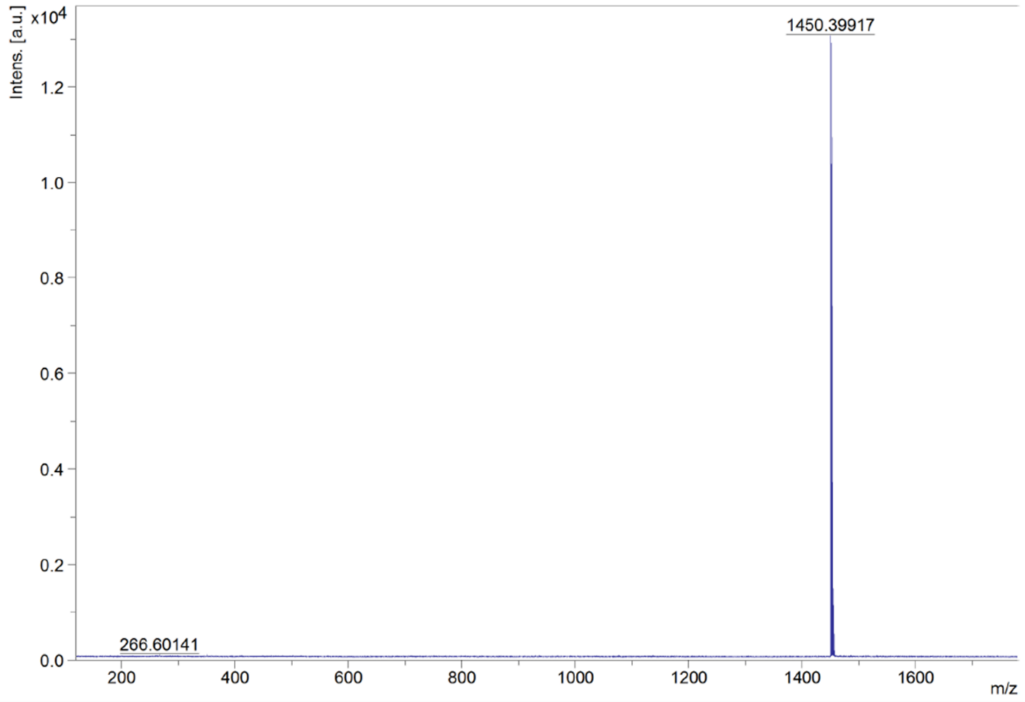

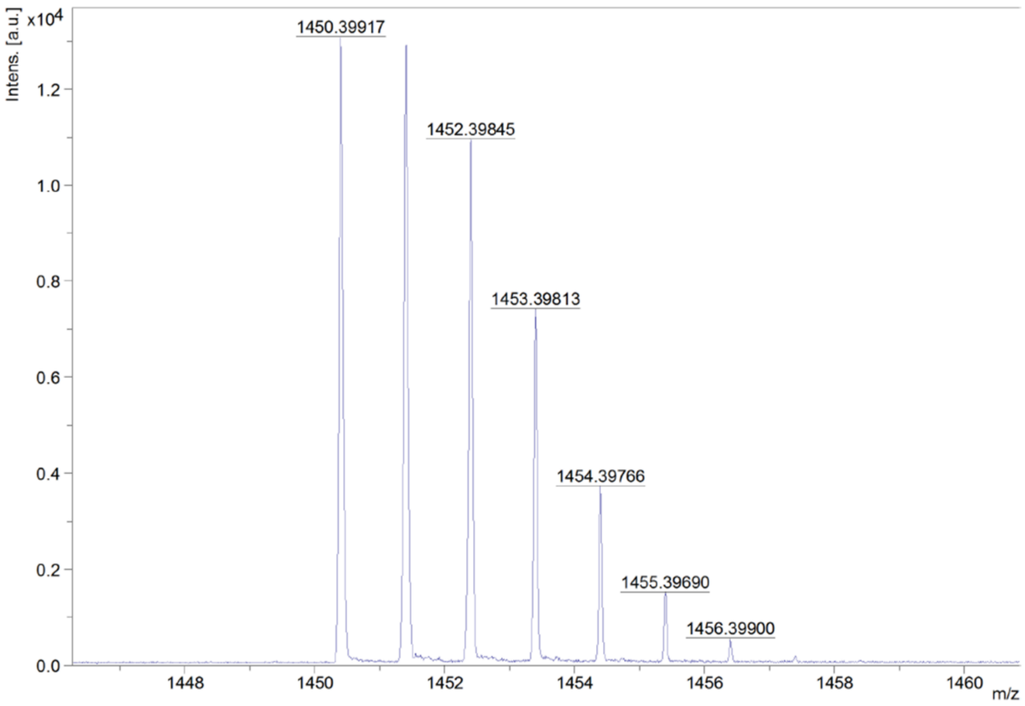

Formula	Mass	Error	mSigma	DblEq	N rule	Electron Configuration
C 88 H 78 N 2 O 4 S 7	1,450.4001	0.6455	30.9377	51.00	ok	odd

Figure S36. HRMS (MALDI-TOF, pos. mode, DCTB in $CHCl_3$) spectra of **7T-PBI**.

Cartesian Coordinates Received from DFT Calculations

Final Geometry:

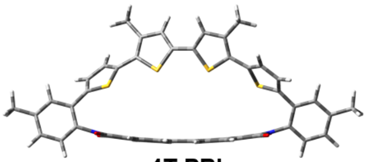

4T-PBI

Total energy: −4156.52965648 Hartrees

C1	−1.38008	−2.4548	2.49154	O25	5.55296	−1.93331	−2.52719	H49	3.57179	−2.353	3.19976
C2	−2.77272	−2.33778	2.54206	C26	−4.9705	−2.00822	1.46299	H50	1.12188	−2.53802	3.34617
C3	−3.51181	−2.25279	1.37498	C27	−5.08095	−2.04637	−1.03646	H51	0.83116	−2.49697	−3.43061
C4	−2.85715	−2.32836	0.12148	O28	−5.56092	−1.91046	2.52724	H52	3.28285	−2.29576	−3.49258
C5	−1.43412	−2.44224	0.06109	N29	−5.65038	−1.83875	0.2388	H53	−8.99081	1.31137	−0.19926
C6	−0.68586	−2.48016	1.2804	O30	−5.76797	−1.99949	−2.04348	H54	−10.11352	−2.67671	0.91918
C7	−3.61717	−2.2719	−1.07194	C31	−7.02679	−1.40547	0.28671	H55	−7.72708	−3.34485	0.85597
C8	−2.98112	−2.37415	−2.29686	C32	7.02092	−1.43428	−0.28672	H56	7.71317	−3.37657	−0.85592
C9	−1.58853	−2.48259	−2.36472	C33	−7.36183	−0.07259	−0.02105	H57	10.10235	−2.71832	−0.91918
C10	−0.79281	−2.48742	−1.21744	C34	−8.72323	0.28037	0.0146	H58	8.99619	1.27442	0.19914
C11	0.78255	−2.4905	1.21756	C35	−9.7282	−0.63263	0.3384	H59	−11.2878	0.86963	0.28823
C12	0.67563	−2.48305	−1.28028	C36	−9.35428	−1.94651	0.65018	H60	−11.68304	−0.5525	1.26766
C13	1.42404	−2.44807	−0.06098	C37	−8.01666	−2.32577	0.62098	H61	−11.72627	−0.65524	−0.49339
C14	2.84752	−2.34	−0.12138	C38	8.00697	−2.35869	−0.62097	H62	11.29133	0.82316	−0.28883
C15	3.60777	−2.28652	1.07203	C39	9.34616	−1.98497	−0.6502	H63	11.72333	−0.70309	0.49369
C16	2.9713	−2.38604	2.29696	C40	9.72552	−0.67264	−0.33846	H64	11.68084	−0.60113	−1.26743
C17	1.57828	−2.48878	2.36483	C41	8.72435	0.24453	−0.01469	C65	6.38627	0.92981	0.41481
C18	1.36994	−2.46061	−2.49142	C42	7.36149	−0.10279	0.02098	C66	6.52603	1.83978	1.43897
C19	2.76304	−2.34927	−2.54196	C43	−11.18084	−0.21759	0.35272	S67	4.87301	1.20986	−0.42572
C20	3.50248	−2.2672	−1.37489	C44	11.17987	−0.26363	−0.35276	C68	5.4269	2.72593	1.58158
C21	5.07246	−2.06701	1.03653	H45	−0.84145	−2.49328	3.43073	C69	7.38044	1.83372	2.10705
C22	4.96216	−2.02862	−1.46293	H46	−3.2923	−2.28207	3.49268	C70	4.42397	2.51903	0.65607
N23	5.64274	−1.86188	−0.23876	H47	−3.58146	−2.33876	−3.19967	H71	5.34936	3.45746	2.37726
O24	5.75967	−2.02287	2.04354	H48	−1.13233	−2.53379	−3.34605	C72	−6.38235	0.95595	−0.41491

H73	−6.51845	1.86663	−1.43894	C82	1.49208	4.84779	0.53316	C91	−3.85198	5.61612	−1.1299
S74	−4.8678	1.22957	0.42543	C83	0.71616	3.77324	0.15343	H92	−3.91439	5.73209	−2.22031
C75	−5.41569	2.74825	−1.58155	H84	1.07982	5.84132	0.67746	H93	−4.85476	5.36876	−0.76907
H76	−7.37296	1.86421	−2.10691	C85	−0.70062	3.77614	−0.1535	H94	−3.56926	6.59077	−0.71783
C77	−4.41352	2.5371	−0.65618	C86	−1.47216	4.85388	−0.5331	C95	3.87498	5.60026	1.13008
H78	−5.33524	3.47958	−2.37712	S87	−1.69569	2.33839	−0.04978	H96	3.9378	5.71589	2.2205
C79	3.15484	3.21169	0.52027	C88	−2.84994	4.56298	−0.73646	H97	4.87675	5.34883	0.76928
C80	2.86866	4.55126	0.73651	H89	−1.05587	5.84574	−0.6773	H98	3.59627	6.57609	0.71807
S81	1.70537	2.33146	0.04957	C90	−3.14157	3.22456	−0.52036				

Final Geometry:

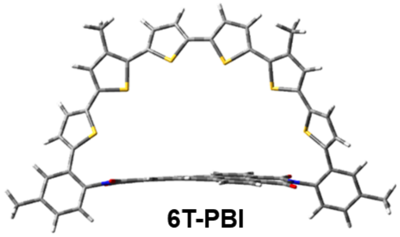

6T-PBI

Total energy: −5260.15960267 Hartrees

C1	−1.24767	−2.70419	2.49259	C18	1.33911	−2.47185	−2.56621	C35	−9.7628	−3.94661	0.78795
C2	−2.64295	−2.72593	2.58879	C19	2.73492	−2.48189	−2.66266	C36	−8.75109	−4.88993	1.01324
C3	−3.42467	−2.77079	1.44706	C20	3.51629	−2.62991	−1.5294	C37	−7.41719	−4.52907	0.87657
C4	−2.80824	−2.75576	0.17238	C21	5.1729	−3.00661	0.80411	C38	7.58513	−4.33556	−1.11811
C5	−1.38337	−2.70511	0.06552	C22	4.99122	−2.68506	−1.67131	C39	8.93911	−4.62523	−1.23494
C6	−0.59373	−2.71697	1.25909	N23	5.73142	−2.89539	−0.48827	C40	9.90158	−3.68376	−0.84609
C7	−3.60933	−2.80042	−0.99503	O24	5.88172	−3.20421	1.777	C41	9.45862	−2.4554	−0.3564
C8	−3.00619	−2.762	−2.2405	O25	5.55371	−2.57148	−2.74899	C42	8.09365	−2.13285	−0.22813
C9	−1.61373	−2.68169	−2.35352	C26	−4.89796	−2.85391	1.58617	C43	−11.21832	−4.32245	0.93751
C10	−0.78218	−2.65515	−1.2321	C27	−5.08416	−2.93797	−0.90959	C44	11.37611	−3.99184	−0.9602
C11	0.87157	−2.7666	1.14645	O28	−5.45736	−2.8565	2.67104	C45	−7.78397	−0.87794	−0.16061
C12	0.68366	−2.59721	−1.33978	N29	−5.64041	−2.95487	0.38872	S46	−6.41231	0.07897	0.36485
C13	1.47298	−2.69604	−0.14993	O30	−5.79482	−3.04867	−1.89457	C47	−6.95266	1.48057	−0.53915
C14	2.89791	−2.73542	−0.25987	C31	−7.05554	−3.23179	0.51191	C48	−8.13687	1.20241	−1.18842
C15	3.69759	−2.8873	0.8991	C32	7.15671	−3.10566	−0.61931	C49	−8.60112	−0.117	−0.97128
C16	3.09408	−2.96752	2.14229	C33	−8.04248	−2.2589	0.27002	C50	7.74613	−0.81842	0.33132
C17	1.70182	−2.89925	2.26121	C34	−9.38733	−2.65373	0.42891	C51	8.37096	−0.18595	1.38402

C52	7.85117	1.09909	1.67575	C73	6.44531	3.92607	1.40989	H94	11.63405	−4.34158	−1.96682
C53	6.82347	1.47976	0.83708	C74	5.46504	4.95208	1.31789	H95	−8.62856	1.91701	−1.83916
S54	6.50604	0.22021	−0.34114	H75	−0.67618	−2.68002	3.41285	H96	−9.49441	−0.51731	−1.43723
C55	−6.21177	2.72497	−0.55765	H76	−3.13419	−2.7226	3.55594	H97	9.15707	−0.66046	1.96102
S56	−4.46353	2.76697	−0.52876	H77	−3.63414	−2.80213	−3.12416	H98	8.19154	1.71232	2.50283
C57	−4.44038	4.52348	−0.60969	H78	−1.18717	−2.65843	−3.34932	H99	−7.78017	4.21004	−0.65249
C58	−5.73095	5.0321	−0.67717	H79	3.72185	−3.08946	3.01851	H100	−3.55712	7.03551	−1.7573
C59	−6.71444	4.00489	−0.6481	H80	1.27427	−2.9706	3.25425	H101	−1.04785	7.68352	−1.49322
C60	−3.1756	5.22602	−0.61999	H81	0.76853	−2.36421	−3.48108	H102	1.21813	7.96381	−0.26919
C61	−2.8473	6.44031	−1.19624	H82	3.22648	−2.38664	−3.62498	H103	3.70718	7.31719	0.16329
C62	−1.48241	6.79011	−1.05815	H83	−10.16034	−1.90479	0.28645	H104	7.41575	4.08276	1.86976
C63	−0.73274	5.85273	−0.37461	H84	−9.00754	−5.90569	1.30358	C105	5.67523	6.32986	1.88741
S64	−1.75575	4.52657	0.14515	H85	−6.63227	−5.25793	1.05354	H106	6.04445	7.03405	1.1296
C65	0.68568	5.86826	−0.09712	H86	6.8406	−5.06924	−1.41115	H107	4.74481	6.7427	2.29087
C66	1.54949	6.9462	−0.09308	H87	9.25022	−5.58858	−1.63139	H108	6.41549	6.30504	2.69365
C67	2.89756	6.59775	0.16587	H88	10.18907	−1.70036	−0.0803	C109	−6.08616	6.49423	−0.72968
C68	3.09521	5.24578	0.38177	H89	−11.86988	−3.44822	0.84456	H110	−5.38134	7.09974	−0.15062
S69	1.56368	4.39141	0.25359	H90	−11.41275	−4.78505	1.91242	H111	−6.08158	6.87991	−1.75825
C70	4.31483	4.52211	0.66941	H91	−11.52147	−5.04695	0.17096	H112	−7.09054	6.66098	−0.32694
S71	4.46354	2.83805	0.18598	H92	11.98756	−3.11058	−0.74359				
C72	6.07184	2.71661	0.86243	H93	11.67143	−4.7826	−0.2588				

Final Geometry:

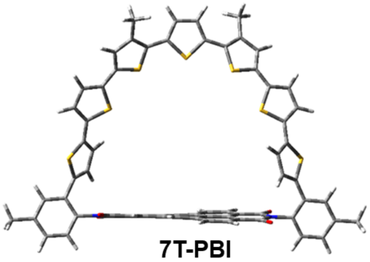

7T-PBI

Total energy: −5811.97392424 Hartrees

C1	1.18375	−2.92766	−2.58157	O25	−5.38053	−3.15916	2.94681	C49	8.88351	−1.36508	1.08153
C2	2.57409	−2.96034	−2.73763	C26	4.86274	−3.24593	−1.84504	C50	−8.0385	−1.82887	−0.09645
C3	3.40055	−3.11638	−1.63753	C27	5.13993	−3.57124	0.62182	C51	−8.8839	−1.3647	−1.08103
C4	2.83737	−3.18241	−0.34025	O28	5.38051	−3.15864	−2.94688	C52	−8.6309	−0.02984	−1.4775
C5	1.42091	−3.10117	−0.16897	N29	5.64135	−3.52257	−0.69834	C53	−7.5901	0.55936	−0.79039
C6	0.58187	−3.03069	−1.32613	O30	5.87676	−3.81632	1.5623	S54	−6.91258	−0.56941	0.37029
C7	3.68254	−3.34419	0.78483	C31	7.01119	−3.93662	−0.91549	C55	7.10725	1.91541	0.94315
C8	3.13327	−3.36602	2.05556	C32	−7.01122	−3.93662	0.91524	S56	5.42877	2.35281	0.68748
C9	1.75112	−3.2371	2.23472	C33	8.11481	−3.14768	−0.54766	C57	5.7377	4.03465	1.08622
C10	0.87607	−3.11318	1.15373	C34	9.39904	−3.67737	−0.78995	C58	7.06822	4.20908	1.41504
C11	−0.87609	−3.11289	−1.1538	C35	9.60804	−4.93225	−1.35881	C59	7.83421	3.02257	1.33515
C12	−0.58189	−3.03096	1.32608	C36	8.48387	−5.69046	−1.71295	C60	4.68731	5.02723	1.07967
C13	−1.42094	−3.10115	0.16891	C37	7.20644	−5.19397	−1.49018	S61	4.65689	6.23582	1.74525
C14	−2.8374	−3.18237	0.34016	C38	−7.20641	−5.1941	1.48967	C62	3.46538	6.98871	1.56169
C15	−3.68257	−3.34386	−0.78495	C39	−8.4838	−5.69069	1.7124	C63	2.55673	6.33931	0.73605
C16	−3.1333	−3.36544	−2.05569	C40	−9.60801	−4.93243	1.35849	S64	3.21259	4.81198	0.16457
C17	−1.75114	−3.23654	−2.23482	C41	−9.39908	−3.67742	0.7899	C65	−2.55656	6.3393	−0.73617
C18	−1.18377	−2.92819	2.58154	C42	−8.11488	−3.14764	0.54763	C66	−3.46521	6.98862	−1.56188
C19	−2.57411	−2.96085	2.73759	C43	11.00375	−5.45961	−1.59495	C67	−4.65676	6.23577	−1.74527
C20	−3.40058	−3.11661	1.63746	C44	−11.00369	−5.45989	1.59457	C68	−4.68723	5.02729	−1.07951
C21	−5.13997	−3.57091	−0.622	C45	8.03833	−1.82903	0.09666	S69	−3.21248	4.81209	−0.16443
C22	−4.86277	−3.24617	1.84495	S46	6.91261	−0.56943	−0.37017	C70	−5.73768	4.03478	−1.08586
N23	−5.64139	−3.5225	0.69818	C47	7.58992	0.55912	0.79086	S71	−5.42878	2.35288	−0.68738
O24	−5.8768	−3.81577	−1.56253	C48	8.63050	−0.03026	1.47814	C72	−7.10736	1.91563	−0.94264

C73	−7.83434	3.02289	−1.33433	H89	−3.02513	−2.89044	3.72166				
C74	−7.06828	4.20934	−1.41431	H90	10.25846	−3.06268	−0.53945	H105	−9.16872	0.47711	−2.27096
C75	1.22076	6.73541	0.34665	H91	8.60846	−6.67048	−2.16681	H106	7.48542	5.17684	1.67086
S76	0.00006	5.51943	0.00042	H92	6.33681	−5.78471	−1.76084	H107	8.90175	2.9814	1.52109
C77	−1.22055	6.7354	−0.34689	H93	−6.33674	−5.78487	1.76016	H108	5.45984	6.56199	2.39843
C78	−0.68275	7.99928	−0.18981	H94	−8.60834	−6.6708	2.16606	H109	−5.45972	6.56187	−2.39848
C79	0.68304	7.99928	0.18924	H95	−10.25854	−3.06271	0.53959	H110	−8.90193	2.98182	−1.51999
C80	3.22423	8.30831	2.24499	H96	11.76202	−4.70733	−1.35728	H111	−7.48548	5.17717	−1.6699
C81	−3.22402	8.3081	−2.2454	H97	11.1448	−5.76097	−2.6398	H112	−1.26176	8.90549	−0.32003
H82	0.57359	−2.82697	−3.47163	H98	11.20431	−6.34276	−0.97518	H113	1.26213	8.90548	0.31922
H83	3.02512	−2.88972	−3.72168	H99	−11.76199	−4.70745	1.3575	H114	3.83973	8.39421	3.14636
H84	3.79389	−3.49823	2.90579	H100	−11.2044	−6.34266	0.97429	H115	3.47871	9.15712	1.59602
H85	1.36507	−3.26807	3.24688	H101	−11.14456	−5.76191	2.63926	H116	2.17473	8.42286	2.53539
H86	−3.79392	−3.49743	−2.90595	H102	9.16816	0.47653	2.27181	H117	−3.83961	8.39392	−3.14672
H87	−1.3651	−3.2673	−3.24698	H103	9.64238	−1.99162	1.53671	H118	−2.17454	8.42252	−2.53594
H88	−0.57361	−2.82772	3.47162	H104	−9.64291	−1.99112	−1.53615	H119	−3.47835	9.15703	−1.5965

Final Geometry:

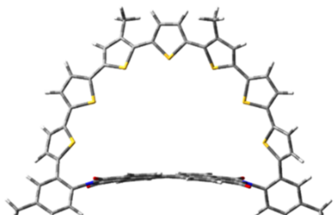

7T-PBI-T

Total energy: −5811.97082142 Hartrees

C1	1.56489	−4.02077	−2.372	C11	−0.6895	−4.18215	−1.27474	C21	−5.00885	−4.25792	−1.4249
C2	2.96246	−3.98392	−2.31094	C12	−0.77935	−4.11429	1.2218	C22	−5.09783	−4.03075	1.06114
C3	3.6142	−4.0559	−1.09167	C13	−1.43141	−4.14793	−0.05153	N23	−5.70481	−4.13525	−0.21084
C4	2.8593	−4.14714	0.10265	C14	−2.85997	−4.14683	−0.10282	O24	−5.61022	−4.35913	−2.4854
C5	1.43074	−4.14808	0.05136	C15	−3.52761	−4.23565	−1.34872	O25	−5.77534	−3.9348	2.0706
C6	0.77868	−4.11437	−1.22197	C16	−2.78953	−4.31019	−2.51778	C26	5.09718	−4.03129	−1.06131
C7	3.52693	−4.23603	1.34855	C17	−1.39114	−4.27536	−2.47834	C27	5.00816	−4.25851	1.42473
C8	2.78883	−4.31048	2.5176	C18	−1.56554	−4.0206	2.37182	O28	5.7747	−3.93548	−2.07077
C9	1.39046	−4.27549	2.47817	C19	−2.96311	−3.9836	2.31077	N29	5.70414	−4.13581	0.21068
C10	0.68882	−4.18222	1.27456	C20	−3.61486	−4.05551	1.09149	O30	5.60952	−4.35984	2.48523

C31	7.15164	−4.17106	0.24036	S61	4.7988	6.78798	−1.16799	H91	9.63099	−6.47522	−0.08191
C32	−7.15231	−4.17031	−0.2405	C62	3.66063	7.64255	−1.11519	H92	7.16262	−6.30013	0.01438
C33	7.91782	−2.9951	0.34173	C63	2.62101	7.08859	−0.38064	H93	−7.16355	−6.2994	−0.01477
C34	9.31657	−3.11739	0.26115	S64	3.09192	5.53394	0.28581	H94	−9.63194	−6.47417	0.08167
C35	9.95616	−4.34818	0.1074	C65	−2.61985	7.08875	0.38063	H95	−9.91649	−2.21373	−0.34482
C36	9.16261	−5.50077	0.03288	C66	−3.6599	7.64348	1.11401	H96	11.92622	−3.4503	0.05903
C37	7.77685	−5.40832	0.09442	C67	−4.79815	6.78903	1.16691	H97	11.78776	−4.94528	−0.87969
C38	−7.77767	−5.4075	−0.09466	C68	−4.65605	5.59256	0.49153	H98	11.86462	−5.01609	0.88167
C39	−9.16344	−5.49977	−0.03304	S69	−3.09039	5.53342	−0.28446	H99	−11.92676	−3.44892	−0.05828
C40	−9.95684	−4.34706	−0.10735	C70	−5.55508	4.46277	0.40639	H100	−11.86547	−5.01421	−0.88187
C41	−9.3171	−3.11634	−0.261	S71	−4.99354	2.93566	−0.25095	H101	−11.78846	−4.94449	0.87952
C42	−7.91835	−2.99423	−0.34168	C72	−6.54499	2.167	0.01365	H102	5.38574	0.60736	2.08204
C43	11.46242	−4.44105	0.03829	C73	−7.41956	3.0537	0.60905	H103	6.03241	−1.92865	2.24987
C44	−11.46311	−4.43975	−0.03817	C74	−6.86753	4.34229	0.82403	H104	−6.03314	−1.92785	−2.25003
C45	7.30929	−1.66721	0.52877	C75	1.2583	7.53524	−0.17647	H105	−5.38585	0.60777	−2.08211
S46	7.75334	−0.31866	−0.50367	S76	6.19E−4	6.32299	3.87E−4	H106	7.4267	5.16497	−1.25596
C47	6.71177	0.7822	0.37806	C77	−1.25711	7.53528	0.17651	H107	8.4439	2.79052	−0.85433
C48	6.06425	0.11181	1.39563	C78	−0.70355	8.79981	0.09539	H108	5.68728	7.03682	−1.73971
C49	6.40235	−1.26194	1.48042	C79	0.70469	8.79978	−0.09604	H109	−5.68707	7.03862	1.73763
C50	−7.30965	−1.66641	−0.52866	C80	3.59133	8.96346	−1.83353	H110	−8.44207	2.79033	0.85857
C51	−6.40281	−1.26118	−1.48043	C81	−3.59103	8.96515	1.83098	H111	−7.42441	5.1645	1.26029
C52	−6.06444	0.1125	−1.39555	H82	1.09814	−3.96379	−3.3483	H112	−1.29485	9.70662	0.14837
C53	−6.71161	0.78288	−0.37772	H83	3.55684	−3.9029	−3.21478	H113	1.29597	9.70657	−0.14957
S54	−7.75322	−0.31792	0.50404	H84	3.3163	−4.38958	3.46228	H114	4.28434	8.983	−2.68093
C55	6.54547	2.16647	−0.01299	H85	0.85475	−4.33103	3.41848	H115	2.58238	9.15738	−2.21278
S56	4.99351	2.93474	0.2495	H86	−3.317	−4.38922	−3.46245	H116	3.85935	9.79977	−1.174
C57	5.55602	4.4624	−0.40576	H87	−0.85544	−4.33098	−3.41865	H117	−4.28506	8.98585	2.67751
C58	6.86914	4.34232	−0.82139	H88	−1.09879	−3.96367	3.34813	H118	−3.85794	9.80084	1.17022
C59	7.42093	3.05362	−0.60644	H89	−3.55748	−3.90252	3.21461	H119	−2.58247	9.15914	2.21126
C60	4.65704	5.59219	−0.49134	H90	9.91607	−2.21487	0.34514				

Final Geometry:

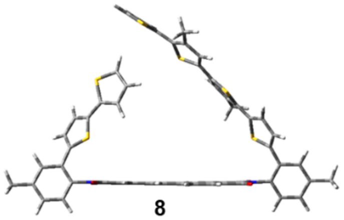

8

Total energy: −5261.35380099

C1	1.77779	−3.56593	−2.29303	O30	6.0657	−2.74727	2.28862	H59	12.07405	−1.29626	−0.09143
C2	3.1715	−3.45118	−2.32148	C31	7.4612	−2.73258	−0.03268	H60	12.23936	−2.9294	−0.75785
C3	3.88461	−3.26425	−1.15002	C32	−6.79659	−3.81695	0.33089	H61	12.17239	−2.69239	0.98958
C4	3.1975	−3.19398	0.08637	C33	8.02992	−1.45133	0.08263	H62	−11.6061	−3.38997	0.20464
C5	1.7723	−3.30329	0.12359	C34	9.43262	−1.35739	0.09741	H63	−11.39856	−4.99804	−0.50246
C6	1.05502	−3.49252	−1.10052	C35	10.26069	−2.47725	−0.0017	H64	−11.43897	−4.80909	1.25154
C7	3.92904	−3.01117	1.28504	C36	9.6585	−3.73553	−0.1325	C65	−7.11586	−1.41118	−0.43865
C8	3.25904	−2.94571	2.49427	C37	8.2732	−3.8588	−0.14296	C66	−7.5668	−0.65981	−1.50014
C9	1.86471	−3.04605	2.53894	C38	−7.35766	−5.04073	0.69086	S67	−5.89946	−0.51645	0.45465
C10	1.0992	−3.21615	1.38369	C39	−8.7368	−5.215	0.69718	C68	−6.93198	0.60285	−1.62472
C11	−0.41153	−3.5965	−1.06285	C40	−9.58655	−4.16142	0.33481	H69	−8.31143	−1.02925	−2.19685
C12	−0.36849	−3.30143	1.4192	C41	−9.00948	−2.93939	−0.01276	H70	−7.13939	1.30295	−2.42712
C13	−1.08573	−3.48996	0.19508	C42	−7.61728	−2.7324	−0.02599	C71	7.23705	−0.21706	0.22203
C14	−2.51287	−3.5711	0.22938	C43	11.76465	−2.33846	0.03378	C72	7.42479	0.78835	1.14307
C15	−3.24448	−3.76291	−0.96786	C44	−11.08604	−4.34549	0.323	S73	5.93509	0.2049	−0.87076
C16	−2.57167	−3.87324	−2.17214	H45	1.26294	−3.70826	−3.23562	C74	6.531	1.87869	0.99456
C17	−1.17597	−3.78917	−2.21525	H46	3.71483	−3.50046	−3.25915	H75	8.17651	0.72816	1.92255
C18	−1.09298	−3.19829	2.60836	H47	3.83652	−2.80867	3.40227	C76	5.64329	1.73272	−0.05386
C19	−2.48953	−3.27366	2.63268	H48	1.38441	−2.98366	3.50804	H77	6.53805	2.75193	1.63823
C20	−3.20262	−3.45837	1.46105	H49	−3.14865	−4.02241	−3.07854	C78	0.59996	6.96125	1.37036
C21	−4.72498	−3.85691	−0.95741	H50	−0.69428	−3.87689	−3.18176	S79	0.27428	5.60046	−0.81987
C22	−4.68276	−3.52529	1.52106	H51	−0.57846	−3.05012	3.55022	C80	1.72088	6.09309	1.2473
C23	−5.35448	−3.71313	0.29719	H52	−3.03566	−3.18602	3.56588	C81	1.72066	5.29982	0.12044
C24	−5.37926	−4.0502	−1.96855	H53	9.87838	−0.36917	0.16951	H82	2.53109	6.08073	1.96986
N25	−5.30443	−3.41862	2.56672	H54	10.27689	−4.62497	−0.22488	C83	2.68977	4.31783	−0.30751
N26	5.35792	−3.11487	−1.21883	H55	7.80792	−4.83561	−0.23091	C84	2.88265	3.78004	−1.56167
O27	5.40592	−2.88193	1.27144	H56	−6.69862	−5.86012	0.96096	S85	3.81483	3.6021	0.82769
O28	5.97526	−3.14502	−2.27244	H57	−9.1558	−6.17582	0.98582	C86	3.92594	2.81705	−1.64812
O29	6.02724	−2.9132	0.00416	H58	−9.65213	−2.10063	−0.26478	H87	2.30274	4.0906	−2.42543

C88	4.55217	2.6122	−0.42702	C97	−1.52564	7.51832	0.00979	C106	−3.94237	2.11369	0.20389
C89	4.28919	2.14208	−2.94587	C98	−2.10684	7.71151	−1.22939	S107	−5.60674	3.56857	−1.12343
H90	5.3713	2.01872	−3.05429	S99	−2.57528	8.16871	1.26389	C108	−3.38942	3.42587	0.17738
H91	3.83952	1.14406	−3.02739	C100	−3.36555	8.37298	−1.17692	H109	−3.45978	1.26638	0.67963
H92	3.92911	2.73147	−3.79525	H101	−1.62814	7.40312	−2.15258	C110	−4.16767	4.31852	−0.50705
C93	0.44834	7.91518	2.5273	C102	−3.74964	8.68577	0.09918	H111	−2.44451	3.69301	0.6376
H94	0.06453	8.8891	2.20685	H103	−3.9521	8.62013	−2.0552	H112	−3.98393	5.36942	−0.68798
H95	−0.24158	7.52824	3.28843	H104	−4.64087	9.20447	0.42594	C113	−0.28306	6.83091	0.30834
H96	1.41415	8.0772	3.0163	C105	−5.14412	2.00745	−0.46278	C114	−5.98759	0.84304	−0.64995

Final Geometry:

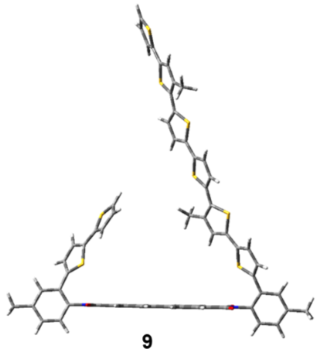

9

Total energy: −6364.98284625

C1	−5.64751	1.28653	2.56757	C17	−5.72784	−1.6738	2.48127	C33	−4.47936	7.74771	0.01882
C2	−5.61086	2.68441	2.59947	C18	−5.98413	−1.52556	−2.37019	C34	−4.55746	9.15234	0.01658
C3	−5.66643	3.41488	1.42516	C19	−6.00445	−2.9238	−2.40298	C35	−5.74193	9.83952	0.28685
C4	−5.76078	2.74125	0.18295	C20	−5.95298	−3.65402	−1.2285	C36	−6.88839	9.0901	0.58254
C5	−5.79479	1.31234	0.14123	C21	−5.85929	−5.21152	1.19861	C37	−6.84164	7.70044	0.58592
C6	−5.73749	0.57728	1.36807	C22	−5.96691	−5.13497	−1.29893	C38	−7.19269	−7.89233	−0.23334
C7	−5.81988	3.49032	−1.01744	N23	−5.91324	−5.82365	−0.07176	C39	−7.2924	−9.27921	−0.21886
C8	−5.91171	2.8331	−2.23194	O24	−5.8423	−5.88245	2.21721	C40	−6.14772	−10.073	−0.06837
C9	−5.94178	1.43557	−2.28184	O25	−6.01446	−5.74414	−2.35656	C41	−4.91247	−9.43266	0.04606
C10	−5.88395	0.65377	−1.12642	C26	−5.615	4.89497	1.49365	C42	−4.78102	−8.0325	0.02856
C11	−5.77276	−0.89235	1.32505	C27	−5.79203	4.97295	−1.00024	C43	−5.78913	11.34932	0.26247
C12	−5.90932	−0.81604	−1.16995	O28	−5.51676	5.50305	2.54838	C44	−6.24983	−11.57983	−0.03035
C13	−5.85373	−1.55094	0.05697	N29	−5.67542	5.58393	0.26669	C45	−3.18762	7.11927	−0.30479
C14	−5.87876	−2.97995	0.01477	O30	−5.8661	5.64536	−2.01534	S46	−2.44948	5.88031	0.6934
C15	−5.82852	−3.72875	1.21565	C31	−5.65367	7.02927	0.30391	C47	−1.03684	5.79033	−0.34431
C16	−5.75619	−3.07139	2.4313	C32	−5.95315	−7.26875	−0.10711	C48	−1.1481	6.69759	−1.3784

C49	−2.35266	7.44483	−1.34952	H76	−6.01147	0.96625	−3.25583	C103	11.2721	1.09985	−2.54357
C50	−3.43485	−7.45319	0.18175	H77	−5.72027	−3.66178	3.34064	H104	10.34637	0.70785	−2.97982
C51	−2.49223	−7.79921	1.1218	H78	−5.66868	−1.20431	3.4559	H105	11.11715	2.17103	−2.36221
C52	−1.25549	−7.11827	0.97529	H79	−6.02537	−0.99731	−3.3152	H106	12.06138	1.00642	−3.29575
S53	−2.77552	−6.25854	−0.92088	H80	−6.05826	−3.45769	−3.34578	C107	1.44305	1.84539	1.71622
C54	0.03344	4.86244	−0.0592	H81	−3.64958	9.71405	−0.18461	H108	1.88244	1.00518	1.16748
S55	1.59242	5.0134	−0.84299	H82	−7.82336	9.59628	0.80977	H109	2.09542	2.04951	2.57492
C56	2.22104	3.58798	−0.01909	H83	−7.73368	7.12046	0.80153	H110	0.47649	1.51821	2.11154
C57	1.26612	3.06014	0.8423	H84	−8.08177	−7.27776	−0.33581	C111	−0.15439	−5.38433	−0.54212
C58	0.04588	3.78737	0.80324	H85	−8.26761	−9.74837	−0.32339	C112	0.01018	−4.76888	−1.76448
C59	3.56769	3.16193	−0.32401	H86	−4.00872	−10.02931	0.13279	S113	1.19051	−4.98965	0.51773
C60	4.34068	3.51736	−1.41625	H87	−4.78532	11.78072	0.20199	C114	1.20284	−3.99645	−1.85799
C61	5.63865	2.96152	−1.41232	H88	−6.27441	11.74829	1.16076	H115	−0.69595	−4.88539	−2.57992
C62	5.90137	2.16087	−0.31674	H89	−6.35978	11.71375	−0.60144	C116	1.9465	−4.02419	−0.71072
S63	4.50068	2.11357	0.74153	H90	−5.26564	−12.0504	−0.11652	H117	1.4977	−3.45026	−2.74743
C64	7.09677	1.4223	0.00778	H91	−6.7001	−11.92259	0.91024	H118	2.89175	−3.53832	−0.50962
C65	7.30623	0.4947	1.01077	H92	−6.87776	−11.95848	−0.84509	C119	14.36182	−1.45312	0.60287
C66	8.60916	−0.04842	1.02419	H93	−0.39601	6.8031	−2.15325	C120	14.63237	−1.91303	1.87514
C67	9.44139	0.44578	0.03391	H94	−2.6228	8.18039	−2.09929	S121	15.73607	−1.75432	−0.45127
S68	8.56534	1.63208	−0.93151	H95	−2.69624	−8.51419	1.91143	C122	15.92466	−2.49361	2.00882
C69	10.82754	0.11311	−0.20144	H96	−0.40323	−7.27604	1.62798	H123	13.9275	−1.82327	2.69503
S70	11.69506	−0.77667	1.04906	H97	−0.82049	3.49965	1.39073	C124	16.63935	−2.47621	0.84273
C71	12.96615	−0.16126	−1.09695	H98	3.96768	4.15025	−2.21426	H125	16.30735	−2.90183	2.93789
C72	11.65931	0.3647	−1.28658	H99	6.35832	3.12564	−2.20746	H126	17.64055	−2.84296	0.66109
H73	−5.60076	0.75808	3.51222	H100	6.53099	0.19773	1.70929	C127	−1.23177	−6.24206	−0.08831
H74	−5.53639	3.21795	3.5411	H101	8.9296	−0.80186	1.7357	C128	13.16733	−0.80996	0.10134
H75	−5.95762	3.42357	−3.14078	H102	13.74856	−0.0514	−1.84156				

Final Geometry:

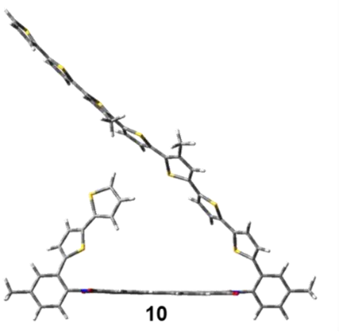

Total energy: −6916.79919220

C1	−4.76574	−3.33627	−2.54625	O28	−2.12305	−6.62247	−2.58872	C55	11.2952	1.82429	0.32048
C2	−3.893	−4.42814	−2.59844	N29	−2.24091	−6.85952	−0.31483	C56	11.73209	1.56278	1.60414
C3	−3.51969	−5.08392	−1.43828	O30	−2.41131	−7.10862	1.95604	C57	13.12616	1.33753	1.6985
C4	−4.0242	−4.64502	−0.18996	C31	−1.35809	−8.00332	−0.37683	C58	9.95625	2.08509	−0.14971
C5	−4.91071	−3.52497	−0.12705	C32	−9.98826	3.38333	0.26725	C59	9.47264	2.08916	−1.44118
C6	−5.28714	−2.86475	−1.33983	C33	0.00582	−7.89373	−0.05293	C60	8.08595	2.37361	−1.56352
C7	−3.6442	−5.32026	0.99541	C34	0.77944	−9.06903	−0.09331	C61	7.48337	2.59282	−0.33063
C8	−4.13606	−4.89196	2.21598	C35	0.24522	−10.31171	−0.43592	S62	8.66648	2.47517	0.96942
C9	−4.99736	−3.79193	2.2872	C36	−1.11526	−10.37901	−0.7647	C63	2.35483	4.1273	−0.20716
C10	−5.39606	−3.09089	1.14743	C37	−1.90465	−9.23529	−0.73123	C64	1.88428	4.98335	−1.1953
C11	−6.19936	−1.71313	−1.2751	C38	−11.36233	3.25306	0.4546	C65	0.48088	5.19058	−1.10429
C12	−6.28637	−1.92223	1.21453	C39	−12.18297	4.37559	0.47613	C66	−0.14241	4.51718	−0.07508
C13	−6.6646	−1.26328	0.00148	C40	−11.64264	5.65651	0.30111	S67	1.03037	3.56038	0.80684
C14	−7.52516	−0.12329	0.06681	C41	−10.2626	5.77092	0.12396	C68	−1.53634	4.50344	0.29794
C15	−7.91904	0.54031	−1.12058	C42	−9.40811	4.65433	0.10452	S69	−2.77789	5.02881	−0.82971
C16	−7.47333	0.07616	−2.34591	C43	1.10822	−11.55161	−0.45466	C70	−4.05772	4.71028	0.33132
C17	−6.62511	−1.03356	−2.41812	C44	−12.53119	6.87834	0.30426	C71	−3.52422	4.20677	1.50086
C18	−6.77405	−1.42437	2.42454	C45	0.66633	−6.64203	0.35224	C72	−2.11423	4.09057	1.48235
C19	−7.60835	−0.30314	2.48044	S46	0.49415	−5.13034	−0.52146	C73	6.10929	2.8766	0.01879
C20	−7.98491	0.3503	1.31982	C47	2.07725	−5.17189	1.51193	S74	4.97023	3.59848	−1.11083
C21	−8.79859	1.73294	−1.07832	C48	1.55732	−6.48616	1.38983	C75	3.69135	3.66432	0.09491
C2	−8.84289	1.55519	1.4169	C49	−7.96856	4.87273	−0.11906	C76	4.1567	3.14918	1.29333
N23	−9.18098	2.18593	0.20297	C50	−7.3932	5.67171	−1.08086	C77	5.49812	2.71052	1.25057
O24	−9.17477	2.31131	−2.0844	C51	−5.9762	5.70998	−1.02609	C78	7.38814	2.39292	−2.8989
O25	−9.23192	2.00358	2.48434	C52	−5.44309	4.9425	−0.01099	C79	2.72746	5.6618	−2.2439
C26	−2.58529	−6.23186	−1.52807	S53	−6.72693	4.1609	0.8939	H80	−5.02687	−2.85295	−3.48007
C27	−2.73108	−6.48876	0.9554	S54	12.65529	1.81769	−0.7929	H81	−3.49296	−4.77643	−3.54472

H82	−3.83672	−5.42356	3.11294	H100	2.76048	−4.86682	2.2975	C118	15.19453	1.24272	0.20424
H83	−5.35232	−3.4908	3.26555	H101	1.79956	−7.29376	2.07205	C119	15.8129	1.01167	−1.00704
H84	−7.78912	0.59468	−3.24488	H102	−7.98092	6.19519	−1.82698	S120	16.39139	1.31193	1.48929
H85	−6.29767	−1.3564	−3.39909	H103	−5.36495	6.28767	−1.71157	C121	17.22677	0.88205	−0.90892
H86	−6.50359	−1.90032	3.35947	H104	11.05948	1.50723	2.45354	H122	15.26407	0.91711	−1.93809
H87	−7.96782	0.07601	3.43115	H105	13.62978	1.09158	2.62738	C123	17.68572	1.01245	0.37303
H88	1.83901	−8.99017	0.13274	H106	10.09666	1.86074	−2.29966	H124	17.8736	0.69108	−1.75828
H89	−1.5589	−11.33014	−1.0485	H107	−0.05386	5.85551	−1.77554	H125	18.70402	0.95242	0.73273
H90	−2.96111	−9.28903	−0.97552	H108	−4.13407	3.94925	2.36034	C126	1.89746	−2.89222	0.37679
H91	−11.78386	2.26035	0.57761	H109	−1.53236	3.73597	2.32655	C127	1.15737	−1.9195	−0.26054
H92	−13.25267	4.25512	0.62871	H110	3.54177	3.09843	2.18549	S128	3.38753	−2.20296	1.00262
H93	−9.81885	6.75674	0.01605	H111	6.01153	2.28171	2.10455	C129	1.76772	−0.6329	−0.23871
H94	2.16898	−11.30306	−0.35237	H112	8.01667	1.92524	−3.66303	H130	0.19134	−2.12014	−0.71183
H95	0.98154	−12.11241	−1.38804	H113	6.43556	1.85302	−2.87105	C131	2.97044	−0.6247	0.41293
H96	0.84521	−12.22981	0.36734	H114	7.17298	3.41577	−3.23401	H132	1.32415	0.2538	−0.67834
H97	−11.94398	7.80082	0.34518	H115	2.17659	6.49224	−2.69634	H133	3.63673	0.2107	0.58367
H98	−13.15127	6.91941	−0.60051	H116	3.00543	4.9748	−3.05364	C134	13.7877	1.42269	0.4904
H99	−13.21319	6.87403	1.16241	H117	3.6567	6.06276	−1.82535	C135	1.59761	−4.2991	0.55912

Final geometry:

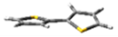

11

Total energy: −1104.81669881 Hartrees

C1	0.00000	3.21057	−0.05036	H7	−2.27318	0.80799	0.4783	C13	0.00000	−3.21057	−0.05036
C2	−1.26892	2.7657	0.19851	C8	0.14867	−0.71017	0.06763	H14	2.11707	−3.42706	0.33872
C3	−1.35485	1.34567	0.26702	C9	1.35485	−1.34567	0.26702	H15	−0.3497	−4.22987	−0.14378
S4	1.11645	1.89317	−0.22313	S10	−1.11645	−1.89317	−0.22313	C16	−0.14867	0.71017	0.06763
H5	0.3497	4.22987	−0.14378	C11	1.26892	−2.7657	0.19851				
H6	−2.11707	3.42706	0.33872	H12	2.27318	−0.80799	0.4783				

Supporting References

[S1] M. Zhang, H. Fan, X. Guo, Y. Yang, S. Wang, Z.-G. Zhang, J. Zhang, X. Zhan, Y. Li, *Polym. Chem.* **2011**, *49*, 2746-2754.

[S2] M.-A. Sato, K. Fukui, *Synth. Met.* **2007**, *157*, 619-626.

[S3] A. L. Jones, M. K. Gish, C. J. Zeman, J. M. Papanikolas, K. S. Schanze, *J. Phys. Chem. A* **2017**, *121*, 9579-9588.

[S4] K. Bold, M. Stolte, K. Shoyama, M. Holzapfel, A. Schmiedel, C. Lambert, F. Würthner, Angew. Chem. Int. Ed. **2022**, *61*, *e202113598*; *Angew. Chem.* **2022**, *134*, *e202113598*.

[S5] K. Bold, M. Stolte, F. Würthner, *Org. Mater.* **2021**, *3*, 119-127.

[S6] N. Auerhammer, A. Schulz, A. Schmiedel, M. Holzapfel, J. Hoche, M. I. S. Röhr, R. Mitric, C. Lambert, *Phys. Chem. Chem. Phys.* **2019**, *21*, 9013-9025.

[S7] G. M. Sheldrick, *Acta Crystallogr. A* **2008**, *64*, 112-122.

[S8] W. Kabsch, *Acta Crystallogr. D* **2010**, *66*, 125-132.

[S9] G. M. Sheldrick, *Acta Crystallogr. A* **2015**, *71*, 3-8.

[S10] G. M. Sheldrick, *Acta Crystallogr. C* **2015**, *71*, 3-8.

[S11] Gaussian 16, Revision B.01, M. J. Frisch, G. W. Trucks, H. B. Schlegel, G. E. Scuseria, M. A. Robb, J. R. Cheeseman, G. Scalmani, V. Barone, G. A. Petersson, H. Nakatsuji, X. Li, M. Caricato, A. V. Marenich, J. Bloino, B. G. Janesko, R. Gomperts, B. Mennucci, H. P. Hratchian, J. V. Ortiz, A. F. Izmaylov, J. L. Sonnenberg, D. Williams-Young, F. Ding, F. Lipparini, F. Egidi, J. Goings, B. Peng, A. Petrone, T. Henderson, D. Ranasinghe, V. G. Zakrzewski, J. Gao, N. Rega, G. Zheng, W. Liang, M. Hada, M. Ehara, K. Toyota, R. Fukuda, J. Hasegawa, M. Ishida, T. Nakajima, Y. Honda, O. Kitao, H. Nakai, T. Vreven, K. Throssell, J. A. Montgomery, Jr., J. E. Peralta, F. Ogliaro, M. J. Bearpark, J. J. Heyd, E. N. Brothers, K. N. Kudin, V. N. Staroverov, T. A. Keith, R. Kobayashi, J. Normand, K. Raghavachari, A. P. Rendell, J. C. Burant, S. S. Iyengar, J. Tomasi, M. Cossi, J. M. Millam, M. Klene, C. Adamo, R. Cammi, J. W. Ochterski, R. L. Martin, K. Morokuma, O. Farkas, J. B. Foresman, and D. J. Fox, Gaussian, Inc., Wallingford CT, **2016**.

[S12] C. Eaborn, K. J. Odell, A. Pidcock, *J. Chem. Soc., Dalton Trans.* **1978**, 357-368.

[S13] S. Shekhar, J. F. Hartwig, *J. Am. Chem. Soc.* **2004**, *126*, 13016-13027.

[S14] J. M. Brown, N. A. Cooley, *Chem. Rev.* **1988**, *88*, 1031-1046.
